# Multi-locus phylogenetic analyses and morphology reveal new species, new records, and taxonomic updates of saprobic *Dothideomycetes* and *Sordariomycetes* from freshwater habitats in China

**DOI:** 10.3897/imafungus.17.188546

**Published:** 2026-06-25

**Authors:** Li Lu, Si-Ping Zheng, Zhi-Jun Zhai, Yin-Ru Xiong, Samantha C. Karunarathna, Saowaluck Tibpromma, Dian-Ming Hu, Hai-Yan Song

**Affiliations:** 1 Bioengineering and Technological Research Center for Edible and Medicinal Fungi, Jiangxi Agricultural University, Nanchang 330045, China Bioengineering and Technological Research Center for Edible and Medicinal Fungi, Jiangxi Agricultural University Nanchang China; 2 Wetland Ecological Resources Research Center, Jiangxi Academy of Forestry, National Ecosystem Research Station of Jiangxi Wugong Mountain Meadow, Jiangxi Nanchang 330013, China College of Biology and Food Engineering, Qujing Normal University Qujing China; 3 Center for Yunnan Plateau Biological Resources Protection and Utilization & Yunnan International Joint Laboratory of Fungal Sustainable Utilization in South and Southeast Asia, College of Biology and Food Engineering, Qujing Normal University, Qujing 655099, China Wetland Ecological Resources Research Center, Jiangxi Academy of Forestry, National Ecosystem Research Station of Jiangxi Wugong Mountain Meadow Nanchang China

**Keywords:** 10 novel taxa, lignicolous freshwater fungi, phylogeny, taxonomy

## Abstract

Lignicolous freshwater fungi are important for decomposing lignocellulose in woody litter, releasing nutrients, and softening wood, making it more palatable to detritus feeders in water. In this study, 13 lignicolous freshwater fungi were collected and isolated from Jiangxi and Zhejiang provinces, China. Combining morphological characteristics with multi-locus phylogenetic analyses (ITS, LSU, SSU, *TEF*1-α, *TUB*2, and *RPB*2), these samples were identified as 10 new species, two new records, and a new collection belonging to 10 genera, eight families, and seven orders. Furthermore, phylogenetic analyses for each genus were updated, and the current research status and existing taxonomic challenges of these genera are discussed. Additionally, four synonymies are proposed within the genus *Distoseptispora*, further clarifying the ambiguous species boundaries in phylogenetic analyses. This study contributes to filling a gap in the research on fungal diversity in East China and improves our understanding of their ecological roles in freshwater ecosystems.

## Introduction

Freshwater fungi are defined as “fungi that for the whole or part of their life cycle rely on freshwater or that use any resource of a predominantly aquatic or semi-aquatic nature as a substratum” (Thomas 1996). This definition has been complemented from a phylogenetic perspective, recognizing that freshwater fungi are often hypothesized to belong to exclusive freshwater or aquatic phylogenetic lineages as freshwater endemics and may be related to terrestrial lineages as freshwater immigrants (El-Elimat et al. 2021; Calabon et al. 2023). As a result, freshwater fungi are a taxonomically and ecologically diverse group distributed worldwide and occur in a variety of freshwater habitats, including ponds, lakes, rivers, streams, swamps, water in tree holes, and artificial habitats such as pools, reservoirs, dams, drainage ditches, and cooling towers (Thomas 1996; Shearer et al. 2004; Hyde et al. 2016a; Grossart et al. 2019; Yang et al. 2023). The most prominent ecological function of freshwater fungi is their contribution to the decomposition and mineralization of organic matter in freshwater ecosystems (Wong et al. 1998; Wurzbacher et al. 2011, 2014; Yang et al. 2023; Shen et al. 2025).

Taxonomically, freshwater fungi are distributed across 13 fungal phyla (Calabon et al. 2020; Shen et al. 2025), with *Ascomycota* being the most speciose and with many reports in *Dothideomycetes* and *Sordariomycetes* (Jones et al. 2014; Luo et al. 2019; Réblová et al. 2020; Calabon et al. 2022). Molecular phylogenetic studies confirm that most aquatic fungi, including aquatic hyphomycetes, evolved from terrestrial ancestors, and their terrestrial occurrence may be more prevalent than previously suspected (Vijaykrishna et al. 2006; Chauvet et al. 2016). Aquatic hyphomycetes, a phylogenetically heterogeneous and polyphyletic group of anamorph-reproducing fungi, constitute the dominant component of freshwater fungi (Hu et al. 2013; Su et al. 2016; Shen et al. 2025; Xu et al. 2025). Some freshwater fungi are adapted to running waters via specialized conidial morphologies that facilitate dispersal and adherence to plant substrata (Chauvet et al. 2016). Freshwater fungi comprise diverse groups of saprobes, parasites, pathogens, endophytes, and mutualists on dead, decaying plant litter or in association with aquatic macrophytes, algae, and fish (Calabon et al. 2023). As important decomposers, they exhibit rapid growth, copious spore production, and specialized propagules for aquatic dispersal (Gulis et al. 2005; Hyde et al. 2016a) and possess the capacity to degrade cellulose, hemicellulose, lignocellulose, and other organic macromolecules (Bucher et al. 2004; Lin et al. 2025). Notably, these fungi play a pivotal role in nutrient and carbon cycling within freshwater ecosystems by decomposing woody litter, softening woody substrates, and releasing bioavailable nutrients (Luo et al. 2019); they can also tolerate environmental stresses, such as pollution and river intermittency, although higher diversity is observed in pristine streams (Chauvet et al. 2016).

While global studies have extensively elucidated the evolutionary origins and ecological roles of freshwater fungi, intensive investigations across diverse geographical regions are essential to fully uncover their global biodiversity. In China, aquatic fungi have been investigated since the 1920s; Hong Kong and Yunnan Province have received much attention for surveys of freshwater fungi (Cai et al. 2002; Hu et al. 2013). In particular, the diversity of lignicolous freshwater fungi in Yunnan has been well investigated in recent years, and it has become one of the major hotspots for lignicolous freshwater fungal research (Hu et al. 2013; Su et al. 2016; Luo et al. 2019; Shen et al. 2022a; Shen et al. 2025). However, the distribution of aquatic fungi remains largely uninvestigated across most regions of China, with numerous provinces yet to be systematically surveyed and their fungal taxa formally described. In East China, characterized by a warm, humid climate and extensive forested nature reserves (e.g., Jiangxi and Zhejiang provinces), substantial inputs of plant litter sustain its perennial fluvial systems. Such an abundant, consistent supply of organic detritus, in conjunction with favorable aquatic habitats, facilitates the colonization and proliferation of freshwater fungi. Consequently, these regions harbor immense yet largely untapped potential for freshwater fungal diversity. Indeed, compared to extensively surveyed hotspots such as Yunnan, the aquatic environments in East China have been severely undersampled. Wang and Cai (2023) reported that a substantial 2,136 fungal species have been discovered in Yunnan; in contrast, only 169 and 141 were reported in Jiangxi and Zhejiang, respectively. More importantly, the vast majority of the taxa described from East China were isolated from terrestrial habitats. This underscores that the seemingly low fungal diversity in these areas is an artifact of insufficient taxonomic investigation in their regional fluvial systems, leaving a substantial taxonomic blind spot.

In this study, a taxonomy and diversity survey of freshwater fungi in Jiangxi and Zhejiang provinces, China, was conducted. Thirteen fungal taxa (12 anamorphs and one teleomorph) were collected. Based on multi-locus phylogenetic analyses and morphological evidence, the present study introduces 10 new species, two new records, and a new collection from new habitats. The results not only expand the global phylogenetic framework of these fungal groups but also emphasize East China as a highly promising, undeveloped, new hotspot of freshwater fungal biodiversity.

## Materials and methods

### Sample collection, morphological observation, and isolation

Submerged decaying wood pieces were collected from freshwater habitats in Jiangxi and Zhejiang provinces, China, from 2023 to 2025. After recording the collection information of the samples (country, province, city, latitude, longitude, altitude, habitat, host name, and date), the samples were placed in zip-lock polythene bags and taken back to the laboratory (Rathnayaka et al. 2025). Following Senanayake et al. (2020), a stereomicroscope (AXIOSKOP 2 PLUS Series, Göttingen, Germany) was used to observe the morphological characteristics (color, shape) of the colonies growing on the sample surface; images of microscopic morphology (conidia/ascospores, conidiogenous cells, conidiophores, and asci) were captured using an Axioskop 2 Plus (Göttingen, Germany) compound microscope equipped with a Canon Axiocam 506 color digital camera (Hanover, Germany). Single-spore isolation was used to obtain pure cultures (Senanayake et al. 2020), and two pure cultures were obtained from distinct single-spore isolations derived from a single specimen (specimen singleton) (Cazabonne et al. 2024); DNA extraction and PCR amplification were performed independently for each pure culture obtained from these isolates. Germinating spores were transferred aseptically to potato dextrose agar (PDA) medium, incubated at 25 °C for 2–4 weeks, and the morphological characteristics of cultures were recorded. The fungal cultures were deposited at the Jiangxi Agricultural University Culture Collection (JAUCC), China, and the herbarium specimens were deposited in the Herbarium of Fungi Jiangxi Agricultural University (HFJAU), China. MycoBank (MB) numbers for the new taxa were obtained as explained in https://www.mycobank.org/.

### DNA extraction, PCR amplification, and sequencing

Genomic DNA was extracted from fresh fungal mycelium grown on PDA for two weeks using a modified cetyltrimethylammonium bromide (CTAB) method: tissue processing followed Gardes and Bruns (1993), while the buffer was optimized for secondary metabolite removal according to Allen et al. (2006). The internal transcribed spacer (ITS) region, large subunit nuclear ribosomal DNA (LSU), small subunit nuclear ribosomal DNA (SSU), translation elongation factor (*TEF*1-α), beta-tubulin 2 (*TUB*2), and RNA polymerase II subunit 2 (*RPB*2) were amplified using the primer pairs ITS1/ITS4 (White et al. 1990), LR0R/LR7 (Hopple and Vilgalys 1999), NS1/NS4 (White et al. 1990), 983F/2218R (Rehner and Buckley 2005), Bt2a/Bt2b (O’Donnell and Cigelnik 1997), and fRPB2-5F/fRPB2-7cR (Liu et al. 1999), respectively. The polymerase chain reaction (PCR) mixture contained a total volume of 25 μL, consisting of 12.5 μL 2 × FastTaq Premix (mixture of FastTaq™ DNA Polymerase, buffer, dNTP Mixture, and stabilizer) (Beijing Qingke Biological Technology Co., Ltd., Beijing, PR China), 1 μL each of forward and reverse primers, 9.5 μL ddH2O, and 1 μL DNA. PCR was performed in a C1000 Touch™ thermal cycler (Lu et al. 2025). PCR products were sequenced by QingKe Biotechnology Co. (Changsha, China).

### Phylogenetic analyses

To clarify the taxonomy of potential new taxa in this study, ITS sequences were initially identified using BLASTn searches against GenBank. Phylogenetic analyses for different fungal groups were conducted using a multi-locus approach. The relevant sequence data were combined with newly generated sequences, and automated alignment and editing were performed with OFPT (Zeng et al. 2023). The sequence alignments were first concatenated into FASTA files and then visually inspected and manually refined where necessary in AliView (Larsson 2014). Maximum likelihood (ML) and Bayesian inference (BI) analyses were used to clarify phylogenetic relationships via the CIPRES Science Gateway portal (https://www.phylo.org/). ML analyses were conducted using RAxML-HPC v.8 with a GTRGAMMA model, followed by rapid bootstrap analysis with 1,000 replicates. MrBayes 3.2.7a was used for BI analyses, and the best nucleotide substitution model for each data partition was selected using MrModeltest 2.2 (Nylander 2004). Bayesian posterior probabilities (BYPP) were estimated using a Markov Chain Monte Carlo (MCMC) approach (Rannala and Yang 1996). Six simultaneous Markov chains were run for 2 million generations, with trees sampled every 1,000^th^ generation. The analyses were considered to have converged when the average standard deviation of split frequencies fell below 0.01, with the first 20% of sampled trees discarded as burn-in; the remaining trees were used to construct a majority-rule consensus tree and to calculate BYPP values (Ronquist and Huelsenbeck 2003). The phylogenetic trees were visualized using FigTree (v. 1.4.2) (Rambaut 2014) and edited using Microsoft Office PowerPoint 2020 and Adobe Photoshop CC 2018 (Adobe Systems Inc., USA). The bootstrap support values for ML ≥ 70% and BYPP ≥ 0.90 are shown above the nodes. Information on all sequences used for phylogenetic analyses is available in the supplementary materials (see Suppl. material 2: tables S1–S8). All obtained alignments and phylogenetic trees were deposited in Figshare (https://doi.org/10.6084/m9.figshare.31292347).

## Results

### Taxonomy

***Ascomycota* Caval. Sm**.

***Dothideomycetes* O.E. Erikss. & Winka, Myconet 1: 5 (1997)**

***Pleosporales* Luttr. ex M.E. Barr, Prodromus to class *Loculoascomycetes* : 67 (1987)**

#### Dictyosporiaceae

Taxon classificationAnimaliaPleosporalesDictyosporiaceae

Boonmee & K.D. Hyde, Fungal Diversity 80: 462 (2016)

3F543F63-2A4E-5227-9F69-5565B59EA094

##### Notes.

*Dictyosporiaceae* was established by Boonmee and D’souza (2016) to accommodate a holomorphic group characterized by cheiroid, digitate, palmate, or dictyosporous, pale brown to dark brown conidia, with *Dictyosporium* designated as the type genus, and is classified within *Pleosporales*, *Dothideomycetes* (Kodsueb et al. 2007; Boonmee and D’souza 2016; Phookamsak et al. 2024). Most genera in *Dictyosporiaceae* are hyphomycetous, although a few possess coelomycetous morphs (Barr 1987a, 1987b; Tanaka et al. 2015; Lu et al. 2025), and the teleomorphs have so far been confirmed only in five genera: *Dictyosporium*, *Gregarithecium*, *Immotthia*, *Pseudocoleophoma*, and *Verrucoccum* (Barr 1987a, 1987b; Tanaka et al. 2015; Lu et al. 2025). Members of *Dictyosporiaceae* are widely distributed worldwide and occur mainly on decaying plant litter and woody substrates in both terrestrial and aquatic habitats; their pathogenicity has not yet been confirmed (Hongsanan et al. 2020; Boonmee et al. 2021; Shen et al. 2022b). Over the past two decades, extensive sampling combined with molecular analyses has led to a rapid increase in the number of genera and species recognized within *Dictyosporiaceae* (Boonmee and D’souza 2016; Phookamsak et al. 2019; Shen et al. 2022b). Currently, the family comprises 23 accepted genera and approximately 178 species (Lu et al. 2025; Wang et al. 2025a).

#### Dendryphiella

Taxon classificationAnimaliaPleosporalesTorulaceae

Bubák & Ranoj., Ann. Mycol. 12 (4): 417 (1914)

0A613F68-27EE-5121-A3FA-741E8CD02C64

##### Notes.

*Dendryphiella*, a dematiaceous hyphomycete genus, was proposed by Ranojevic (1914) and typified by *D. interseminata*, which is currently considered a synonym of *D. vinosa* based on morphology (Reisinger 1968). The anamorph is characterized by macronematous, septate, and pigmented conidiophores with terminal or intercalary polytretic conidiogenous cells that are enlarged at the apex, with dark scarring on the nodose swellings, producing acropleurogenous, solitary or catenate conidia, which are commonly multi-septate and cylindrical with rounded ends (Ellis 1971; Du et al. 2025). Currently, no *Dendryphiella* species have been linked with their teleomorph (Liu et al. 2017). In this study, a new species of *Dendryphiella*, collected from the submerged, decaying stem of *Nelumbo nucifera* in freshwater in Jiangxi Province, is introduced.

#### Dendryphiella loti

Taxon classificationAnimaliaPleosporalesTorulaceae

L. Lu, D.M. Hu & H.Y. Song sp. nov.

568E4DCA-C6AE-53CA-8C3A-6494C0C64AA0

903572

Fig. 2

##### Etymology.

Lotus, from Latin, refers to the host, *Nelumbo nucifera*.

##### Diagnosis.

Differs from other *Dendryphiella* species by the conidiophore swollen at the nodules, 1–3-septate and smooth-walled conidia with thickened and darkened scars.

##### Holotype.

HFJAU10951.

##### Description.

***Saprobic*** on a decaying stem of ***Nelumbo nucifera*** in freshwater habitats. **Teleomorph**: Undetermined. **Anamorph**: Hyphomycetous. ***Colonies*** on natural substrate superficial, dark brown to black, effuse, velvety. ***Mycelium*** partly immersed in the substrate, partly superficial, composed of smooth, septate hyphae. ***Conidiophore*** 150−250 × 6.5−8.5 μm (x̄ = 190 × 7.6 μm, *n* = 30), macronematous, mononematous, branched, erect, occasionally fasciculate, straight to flexuous, septate, brown, paler towards the apex, swollen at the nodules, thick-walled. ***Conidiogenous cells*** 18−20 × 4.5−5.5 μm (x̄ = 19.6 × 5 μm, *n* = 30), holoblastic, polytretic, terminal, integrated, cylindrical, yellow to pale brown. ***Conidia*** 13−25 × 5.5−7.5 μm (x̄ = 18.7 × 6.4 μm, *n* = 30), catenate, oblong to subcylindrical, with rounded ends, hyaline to pale brown and aseptate when young, brown and 1–3-septate when mature, slightly constricted at the medial septum, smooth-walled, conidial scars often thickened and darkened.

##### Culture characteristics.

Conidia germinating on PDA within 24 h. Colonies grew on PDA, reaching 40 mm in 30 days at 25 °C, circular, surface white to pale grey, centrally floccose with dense aerial mycelium, outer zone compact, with entire margin, flat. Reverse yellowish to light grey. Not producing pigmentation on PDA medium.

##### Material examined.

CHINA • Jiangxi Province: Yingtan City, Guixi County, Liukou Town, Guitang Road, 28°1'N, 117°13'E, 57 m, on submerged stem of *Nelumbo nucifera* in freshwater, 25 Oct. 2025, Li Lu, LHWYT1-10 (HFJAU10951, holotype); ex-type living culture JAUCC 8231, other living culture JAUCC 8232.

##### Notes.

**Figure 1.**
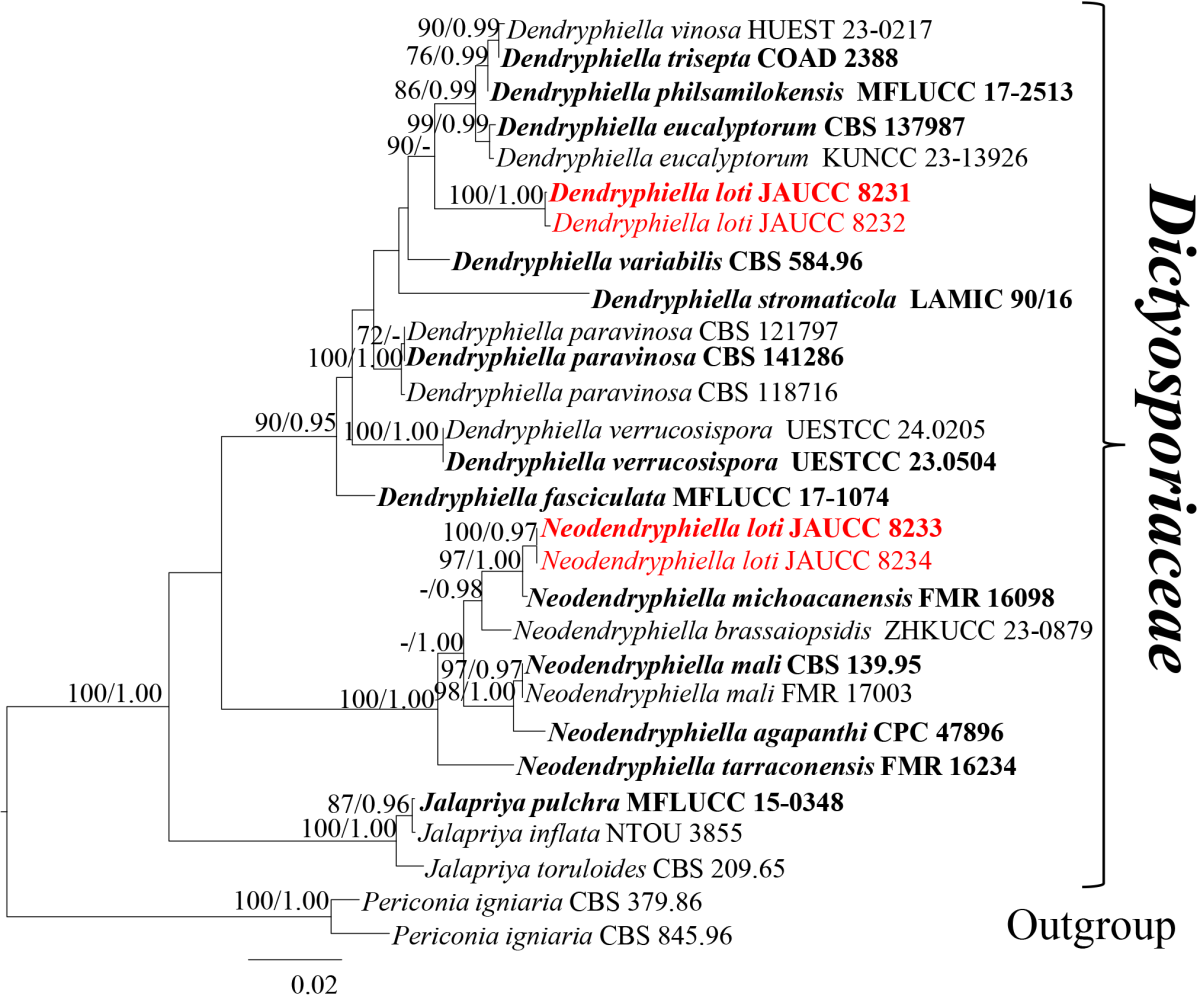
Phylogram generated from maximum likelihood analysis based on combined ITS, LSU, *RPB*2, and *TEF*1-α sequence data of 28 taxa, which comprised 3,093 characters. The best-scoring RAxML tree with a final likelihood value of –8314.538900 is presented. The matrix contained 436 distinct alignment patterns, with 43.52% of characters undetermined (gaps). Estimated base frequencies were as follows: A = 0.237646, C = 0.244160, G = 0.265263, T = 0.252930; substitution rates: AC = 1.741921, AG = 3.074104, AT = 2.518123, CG = 0.679886, CT = 7.196374, GT = 1.0; gamma distribution shape parameter α = 0.078855. Bootstrap support values for ML of at least 70% and BYPP of at least 0.90 are indicated at the nodes as ML/BYPP. Type specimens are in bold, and the new species from the current study are indicated in bold red. The tree is rooted to *Periconia igniaria* (CBS 379.86 and CBS 845.96).

**Figure 2.**
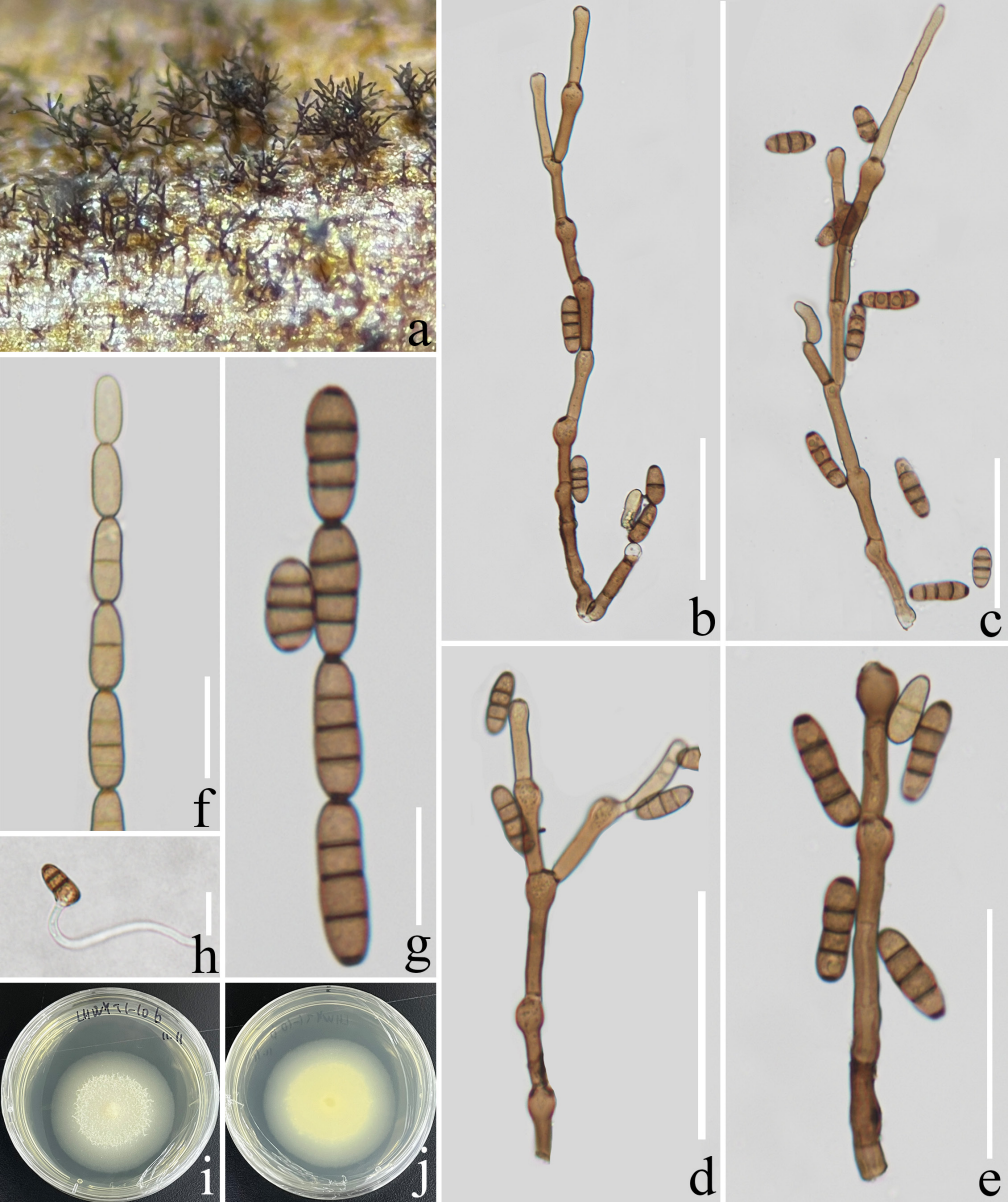
*Dendryphiella loti* (holotype, HFJAU10951) **a** Colonies on a submerged stem. **b–e** Conidiophores with attached conidiogenous cells and conidia. **e** Conidiogenous cell and conidium. **f, g** Conidia. **h** Germinated conidium. **i, j** Culture on PDA from above and below. Scale bars: 50 μm (**b–e**); 20 μm (**g–h**).

In the combined multi-locus phylogenetic tree, *Dendryphiella loti* forms a separate lineage within *Dendryphiella*, with 90% ML statistical support (Fig. 1). The sequence similarity between the two strains (JAUCC 8231 and JAUCC 8232) is above 99% for the analyzed genes, confirming their conspecificity. The BLASTn searches of ITS, LSU, and *TEF*1-α sequences showed 89%, 99%, and 97% similarity to *D. eucalyptorum* (PP925612, MN999929, and PP926518, respectively), and the SSU sequence shows 99% similarity to *D. phitsanulokensis* (NG_065729). Morphologically, *D. loti* aligns with the general descriptions of the genus *Dendryphiella* by having polytretic and integrated conidiogenous cells with septate, catenate conidia (Hyde et al. 2018; Du et al. 2025). *Dendryphiella loti* (HFJAU10951, holotype) can be distinguished from other *Dendryphiella* species by conidiophores swollen at the nodules and 1–3-septate and smooth-walled conidia with thickened and darkened scars (Fig. 2), while other species exhibit polytretic conidiophores, and the conidia have a verrucose surface texture (Hyde et al. 2018; Du et al. 2025). Therefore, *D. loti* is introduced as a new species, the second species of this genus reported from aquatic habitats.

#### Neodendryphiella

Taxon classificationAnimaliaPleosporalesDictyosporiaceae

Iturrieta-Gonz., Dania García & Gené, MycoKeys 37: 25 (2018)

E17BA6C5-70DB-5037-92EA-8227D93AA098

##### Notes.

*Neodendryphiella* was proposed to accommodate three species, *N. mali*, *N. michoacanensis*, and *N. tarraconensis* (type) (Iturrieta-González et al. 2018). Among them, *N. mali* was isolated from herbivore dung and plant leaves of *Malus domestica* in Spain and Italy, respectively, while the other two were isolated from soil in Mexico and Spain (Iturrieta-González et al. 2018). Later, *Neodendryphiella tarraconensis* was re-isolated from decaying wood from China (Guizhou Province) (Hyde et al. 2020c). Subsequently, *N. agapanthi* (isolated from stalks of *Agapanthus praecox*, Brazil-Minas Gerais) and *N. brassaiopsidis* (recovered from dead stems of *Brassaiopsis hainla*, China, Yunnan Province) were introduced as new species based on molecular phylogeny and morphological characters (Dong et al. 2023; Crous et al. 2025). To date, only five species of *Neodendryphiella* have been listed in Index Fungorum (2026). *Neodendryphiella* is characterized by polytretic, integrated conidiogenous cells that produce acropetal, branched chains of conidia (Iturrieta-González et al. 2018). In this study, a new species of *Neodendryphiella*, collected from the submerged, decaying stem of *Nelumbo nucifera* in freshwater in Jiangxi Province, is introduced.

#### Neodendryphiella loti

Taxon classificationAnimaliaPleosporalesDictyosporiaceae

L. Lu, D.M. Hu & H.Y. Song sp. nov.

9969FD28-B7D6-54E0-9713-5A44102FEED7

903573

Fig. 3

##### Etymology.

Lotus, from Latin, refers to the host, *Nelumbo nucifera*.

##### Diagnosis.

Differs from *N. michoacanensis* by the smaller conidiogenous cells and conidia, and the conidia of *N. loti* with thickened and darkened scars, 1-septate with dense central contents.

##### Holotype.

HFJAU10952.

##### Description.

***Saprobic*** on a decaying stem of ***Nelumbo nucifera*** in freshwater habitats. **Teleomorph**: Undetermined. **Anamorph**: Hyphomycetous. ***Colonies*** on natural substrate superficial, effuse, pale brown, velvety. ***Mycelium*** mostly immersed in the substrate, composed of septate, branched, hyaline to pale brown hyphae. ***Conidiophores*** 200−450 × 4−7 μm (x̄ = 364 × 5.6 μm, *n* = 30), macronematous, mononematous, erect or slightly flexuous, unbranched, septate, cylindrical, reddish-brown at base, slightly paler towards the apex, smooth to finely verrucose, thin-walled. ***Conidiogenous cells*** 18−22 × 4−5 μm (x̄ = 20.1 × 4.5 μm, *n* = 30), polyblastic, terminal and intercalary, cylindrical, forming conidia in acropetal branched chains. ***Ramoconidia*** 15−19 × 5−7 μm (x̄ = 17 × 5.9 μm, *n* = 30), 0–1-septate, hyaline to pale brown, surface smooth, subcylindrical. ***Conidia*** 10−14 × 4−6 μm (x̄ = 12.8 × 5.4 μm, *n* = 30), catenate, ellipsoidal or subcylindrical with both rounded ends, 0–2-septate, some slightly constricted at the septum, light brown, smooth-walled, with dense central contents.

##### Culture characteristics.

Conidia germinating on PDA within 24 h. Colonies grew on PDA, reaching 20 mm in 30 days at 25 °C, circular, umbonate in the middle, entire to slightly undulate margin, with a dense, cottony to floccose central tuft, surrounded by a paler, felty to velvety zone, surface with aerial mycelium, pale grey to greyish brown. Reverse pale brown to greyish brown, without diffusible pigments. Not producing pigmentation on PDA medium.

##### Material examined.

CHINA • Jiangxi Province: Yingtan City, Guixi County, Liukou Town, Guitang Road, 28°1'N, 117°13'E, 57 m, on submerged stem of *Nelumbo nucifera* in freshwater, 25 Oct. 2025, Li Lu, LHWYT1-5 (HFJAU10952, holotype); ex-type living culture JAUCC 8233, other living culture JAUCC 8234.

##### Notes.

**Figure 3.**
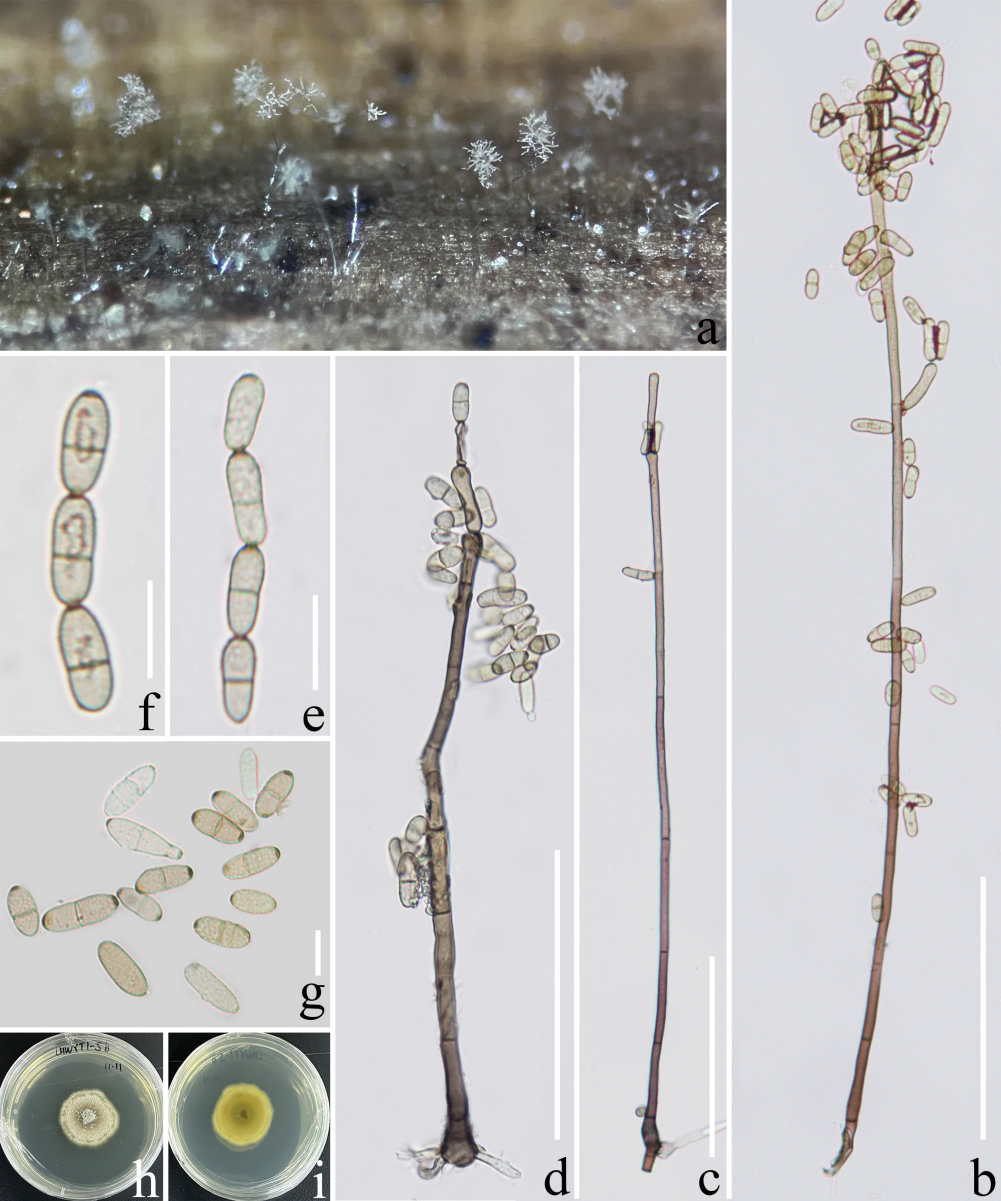
*Neodendryphiella loti* (holotype, HFJAU10952) **a** Colonies on a submerged stem. **b–d** Conidiophores with attached conidiogenous cells and conidia. **e–g** Conidia. **h, i** Culture on PDA from above and below. Scale bars: 100 μm (**b–d**); 10 μm (**e–g**).

In the phylogenetic tree, *Neodendryphiella loti* forms a sister lineage with *N. michoacanensis* (FMR 16098, ex-type) (97% ML, 1.00 BYPP; Fig. 1). The sequence similarity between the two strains (JAUCC 8233 and JAUCC 8234) is above 99% for the analyzed genes, confirming their conspecificity. The BLASTn search of the ITS sequence shows 99% similarity with *N. michoacanensis* (LT906660), and the LSU sequence shows 98% similarity with *N. tarraconensis* (PQ671127). Members of *Neodendryphiella* only have the ITS and LSU genes available in GenBank. Morphologically, *N. loti* aligns with the general descriptions of *Neodendryphiella*, such as polytretic conidiogenous cells that form conidia in acropetal, branched chains (Iturrieta-González et al. 2018). However, *N. loti* (HFJAU10952, holotype) can be distinguished from other *Neodendryphiella* species by the light brown and smooth-walled conidia, with dense central contents, while other species have verruculose to verrucose conidia, with scars thickened and darkened (Iturrieta-González et al. 2018; Dong et al. 2023; Crous et al. 2025; Fig. 3). *Neodendryphiella loti* can be morphologically distinguished from *N. michoacanensis* by the smaller conidiogenous cells (18–22 × 4–5 μm vs. 11–62 × 3–5 μm) and conidia (10−14 × 4−6 μm vs. 16–18 × 3–6 μm); the conidia of *N. loti* have thickened and darkened scars, are 1-septate, and are smooth-walled with dense central contents, whereas *N. michoacanensis* conidia are verruculose to verrucose and 1(–2)-septate (Iturrieta-González et al. 2018; Fig. 3). Therefore, *N. loti* is introduced as a new species in *Neodendryphiella*. Furthermore, this is the first time that species of this genus have been reported from aquatic habitats.

### *Sordariomycetes* O.E. Erikss. & Winka, Myconet 1(1): 10 (1997)

***Chaetosphaeriales* Huhndorf, A.N. Mill. & F.A. Fernández, Mycol. Res. 108 (12): 378 (2004)**

#### Helminthosphaeriaceae

Taxon classificationAnimaliaMonhysteridaHelminthosphaeriaceae

Samuels, Cand. & Magni, Mycologia 89: 144 (1997)

D95DF54F-1F7C-58CA-9953-4F2B5BCD517E

##### Notes.

*Helminthosphaeriaceae* was established for the type genus *Helminthosphaeria* (Samuels et al. 1997). Most taxa in this family are rare, occurring as small clusters or isolated individuals on diverse substrates, such as decaying wood, sphagnum moss, or old basidiomata. In addition, many species do not grow in culture, which has severely limited the availability of molecular data (Miller et al. 2014). This has hindered the phylogenetic resolution of the group, leaving many genus-level classifications unsupported or poorly defined, which has resulted in the prevalence of polyphyletic or paraphyletic groupings within the family. LSU and *TUB*2 markers provide good suprageneric resolution (Miller et al. 2014; Yang et al. 2023; Lu et al. 2025). Morphologically, most taxa are characterized by ascomata with distinct thick-walled setae, a key diagnostic feature for identification (Miller et al. 2014). Notably, the placement of *Selenosporella curvispora* in this family has been confirmed (Réblová et al. 2021), and the *Selenosporella* phenotype is widespread among its members. The family encompasses diverse modes of conidiogenesis, including tretic (*Diplococcium*), holoblastic (*Endophragmiella*), and holoblastic-denticulate (*Selenosporella* and *Selenosporella*-like). For instance, *Helminthosphaeria* has a *Diplococcium* anamorph, some forming *Selenosporella*-like synanamorphs, while *Endophragmiella dimorphospora*, *Echinosphaeria canescens*, *Hilberina punctata*, and *Ruzenia spermoides* are also linked to *Selenosporella*-like synanamorphs (Miller et al. 2014; Réblová et al. 2021). To date, the family comprises six genera: *Echinosphaeria*, *Helminthosphaeria*, *Hilberina*, *Kramasamuha*, *Ruzenia*, and *Selenosporella* (Hyde et al. 2024).

#### Hilberina

Taxon classificationAnimaliaMonhysteridaHelminthosphaeriaceae

Huhndorf & A.N. Mill., Mycol. Res. 108 (1): 31 (2004)

B73D852A-5271-536A-8F0B-F881D5AF0B34

##### Notes.

Based on nuclear LSU sequences, *Hilberina* was proposed to accommodate *Leptospora caudata* by Miller and Huhndorf (2004), with *H. caudata* as the type species. *Hilberina* species can be distinguished by the presence of setose ascomatal vestitures and cylindrical ascospores that taper to a distinct point at one end (Miller and Huhndorf 2004; Miller et al. 2014). In this study, a new species of *Hilberina* collected from submerged wood in freshwater in Jiangxi Province is described.

#### Hilberina jiangxiensis

Taxon classificationAnimaliaMonhysteridaHelminthosphaeriaceae

S.P. Zheng, D.M. Hu & H.Y. Song sp. nov.

7845406E-16F1-5A41-9F17-F68B41DD58BE

903574

Fig. 5

##### Etymology.

The species epithet refers to the type locality “Jiangxi,” China.

##### Diagnosis.

Differs from *H. hongheensis* by the fusiform conidia with distinctly granular.

##### Holotype.

HFJAU10550.

##### Description.

***Saprobic*** on decaying submerged wood in freshwater habitats. **Teleomorph: *Ascomata*** 100–150 × 120–200 µm (x̄ = 117 × 142 µm, *n* = 15), superficial, ovoid to obpyriform, scattered to loosely clustered, carbonaceous, dark brown to black, setiferous on the surface of the substrate, with a long black papilla visible at the surface, with a developed ostiole. ***Setae*** up to 150 µm long, pale brown, sub-hyaline at the tip, septate lumen, straight, smooth, thick-walled with a narrow acute apex. ***Peridium wall*** 20–35 µm wide (x̄ = 28 µm, *n* = 30), composed of several layers, thick-walled, comprising hyaline to brown cells of ***textura angularis***. ***Hamathecium*** 2–3.5 µm wide (x̄ = 2.9 µm, *n* = 30), hyaline, numerous, filamentous, septate, paraphyses. ***Asci*** 90–140 × 12–18 µm (x̄ = 114 × 15 µm, *n* = 30), unitunicate, 8-spored, cylindrical to subcylindric-clavate, hyaline, short pedicellate, with J-, apical ring. ***Ascospores*** 19–25 × 4.5–7 µm (x̄ = 22.7 × 5.6 µm, *n* = 30), uni-seriate to overlapping, fusiform, hyaline, thin and smooth-walled, aseptate, occasionally 1-septate, granular, without a gelatinous sheath and appendages. **Anamorph**: Undetermined.

##### Culture characteristics.

Ascospores germinating on PDA within 24 h. Colonies grew on PDA, reaching 30 mm in 30 days at 25 °C, irregular, flat, sparse, and irregular margin, dark green from forward, dark brown from reverse.

##### Material examined.

CHINA • Jiangxi Province: Nanchang City, Xinjian County, Meiling Village, 28°76'27"N, 115°67'89"E, 495 m, on submerged unidentified wood in freshwater, 24 Sep. 2024, S.P. Zheng, zsp 66 (HFJAU10550, holotype); ex-type living culture JAUCC 7185, other living culture JAUCC 7186.

##### Notes.

**Figure 4.**
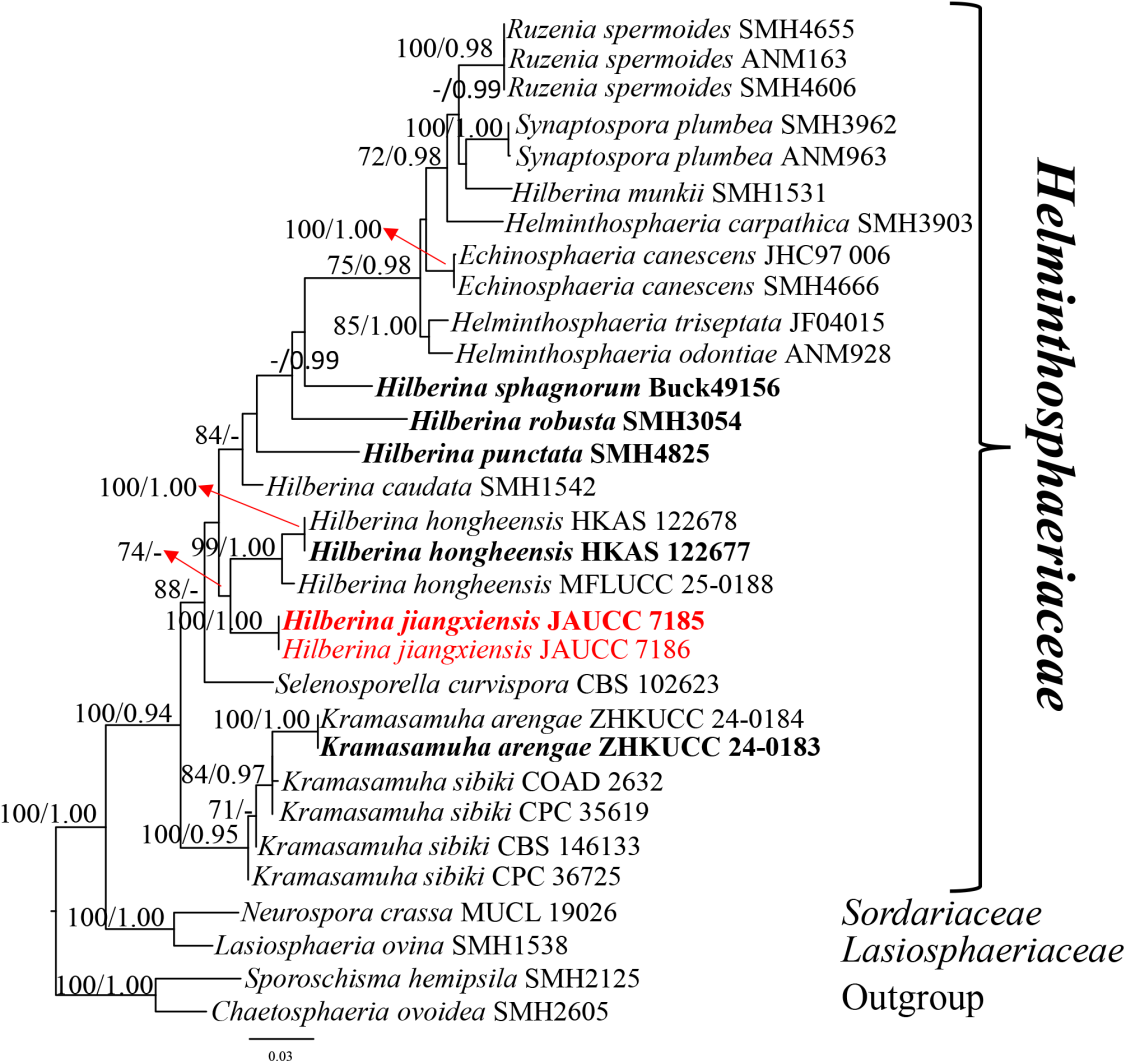
Phylogram generated from maximum likelihood analysis based on combined LSU and *TUB*2 sequence data of 31 taxa, which comprised 1,620 characters. The best-scoring RAxML tree with a final likelihood value of –6474.538992 is presented. The matrix contained 469 distinct alignment patterns, with 25.69% of characters undetermined (gaps). Estimated base frequencies were as follows: A = 0.218209, C = 0.281346, G = 0.320767, T = 0.179678; substitution rates: AC = 0.554249, AG = 1.937404, AT = 0.667956, CG = 0.995905, CT = 4.747433, GT = 1.0; gamma distribution shape parameter α = 0.227681. Bootstrap support values for ML of at least 70% and BYPP of at least 0.90 are indicated at the nodes as ML/BYPP. Type specimens are in bold, and the new species from the current study are indicated in bold red. The tree is rooted to *Chaetosphaeria ovoidea* (SMH2605) and *Sporoschisma hemipsila* (SMH2125).

**Figure 5.**
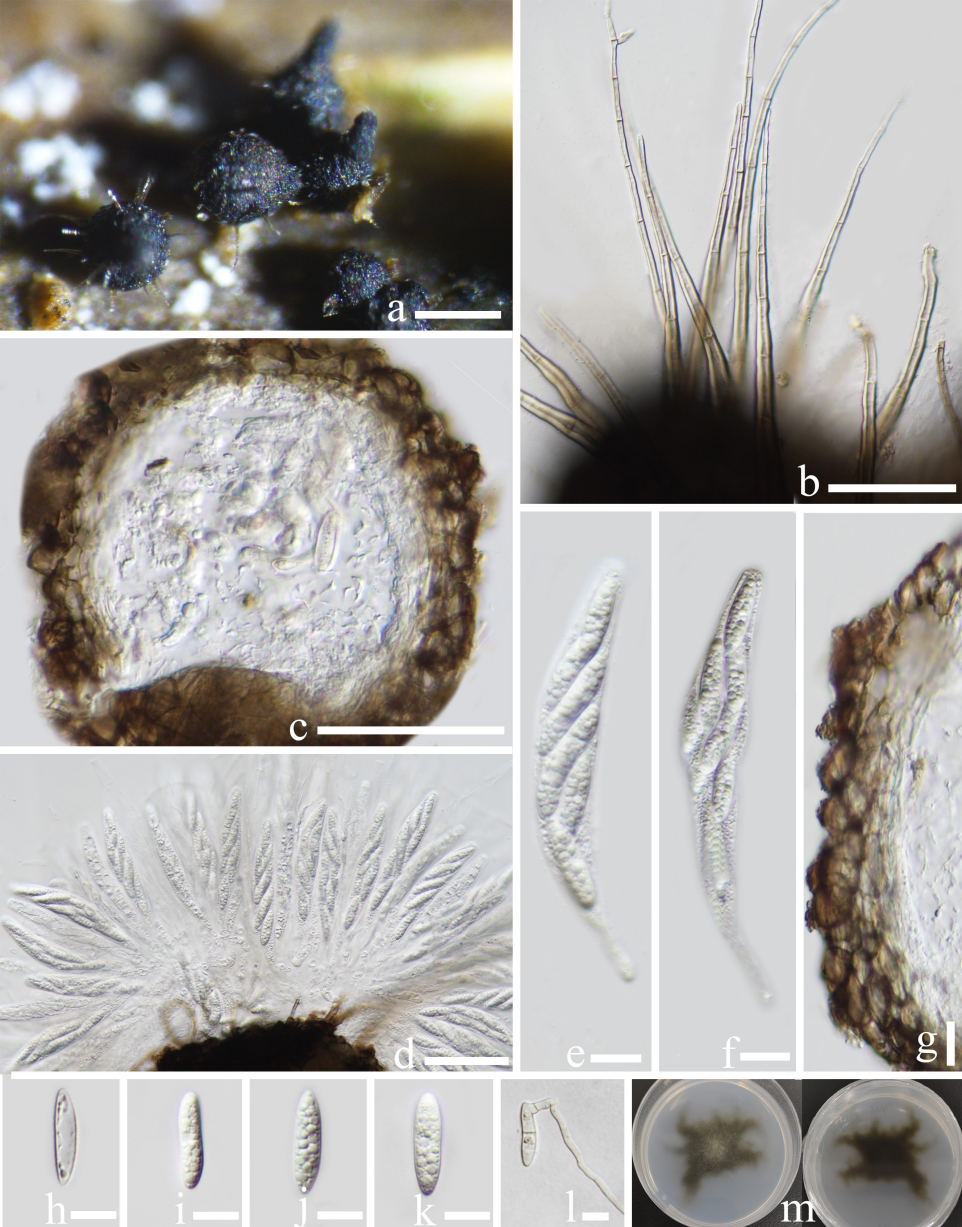
*Hilberina jiangxiensis* (holotype, HFJAU10550) **a** Ascomata on substrate. **b** Setae. **c** Vertical section of ascoma. **d–f** Asci. **g** Paraphyses. **h–k** Ascospores. **l** Germinated ascospore. **m** Culture on PDA from above and below. Scale bars: 100 μm (**a**); 50 μm (**b–d**); 10 μm (**e–l**).

In the phylogenetic tree, *Hilberina jiangxiensis* forms a sister lineage with *H. hongheensis*, with 74% ML statistical support (Fig. 4). The sequence similarity between the two strains (JAUCC 7185 and JAUCC 7186) is above 99% for the analyzed genes, confirming their conspecificity. Based on pairwise nucleotide comparisons, *H. jiangxiensis* (JAUCC 7185, ex-type) diverges from *H. hongheensis* (HKAS 122677, ex-type) by 30/834 bp (3.6%, without gaps) for LSU. Morphologically, *H. jiangxiensis* aligns with the general descriptions of the genus *Hilberina*, such as brown to dark brown and superficial ascomata and septate-lumen setae (Miller et al. 2014; Yang et al. 2023). *Hilberina jiangxiensis* exhibits fusiform conidia (19–25 × 4.5–7 µm), distinct from the oval and smaller conidia observed in *H. hongheensis* (13–16 × 6–9 μm) (Yang et al. 2023; Fig. 5). In addition, morphological comparisons were made with species for which molecular data were unavailable (viz., *H. breviseta*, *H. elegans*, *H. foliicola*, *H. moseri*, *H. meznaensis*, and *H. rhynchospora*). *Hilberina jiangxiensis* can be distinguished by fusiform ascospores with distinct granules, while other species have cylindrical ascospores, with a basal end that is attenuate and acerose (Miller et al. 2014). Therefore, *H. jiangxiensis* is introduced as a new species in *Hilberina*. Furthermore, this is the first time that species of this genus have been reported from aquatic habitats.

### *Distoseptisporales* Z.L. Luo, H.Y. Su & K.D. Hyde, Fungal Diversity 99: 482 (2019)

#### Distoseptisporaceae

Taxon classificationAnimaliaDistoseptisporalesDistoseptisporaceae

K.D. Hyde & McKenzie, Fungal Diversity 80: 402 (2016)

03B174AA-48B1-56FD-8703-10D8D5760D4A

##### Notes.

*Distoseptisporaceae* was proposed for some *Sporidesmium*-like taxa that cluster in a distinct lineage within *Sordariomycetes* (Su et al. 2016), with *Distoseptispora* as the type genus. Then, Luo et al. (2019) placed *Distoseptisporaceae* in the new order *Distoseptisporales* based on phylogenetic analysis of combined LSU, SSU, *RPB*2, and *TEF*1-α sequences. The family *Distoseptisporaceae* can be distinguished from *Sporidesmiaceae* with strong molecular support.

#### Distoseptispora

Taxon classificationAnimaliaDistoseptisporalesDistoseptisporaceae

K.D. Hyde, McKenzie & Maharachch., Fungal Diversity 80: 402 (2016)

7AFDDF50-6E7E-503F-A608-C0BAA53BD861

##### Notes.

*Distoseptispora* was introduced by Su et al. (2016) and typified by *D. fuminicola*. The anamorph of *Distoseptispora* has been emended by Yang et al. (2018) and Delgado et al. (2025). Only two species have been reported in the teleomorph (*D. hyalina* and *D. licualae*) (Yang et al. 2021; Konta et al. 2023). In this study, the *Distoseptispora* phylogenetic tree was updated to include all 117 known species; one new species and one new record were introduced; and four species mergers within ambiguous clades were proposed based on morphology and nucleotide differences.

#### Distoseptispora aquatica

Taxon classificationAnimaliaDistoseptisporalesDistoseptisporaceae

Z.L. Luo, H.Y. Su & K.D. Hyde, Fungal Diversity 80: 404 (2016)

891832D0-6DEA-5837-AF83-0EDE40DB6442

551834

##### Synonym.

*Distoseptispora nanchangensis* Y.F. Hu & Jian Ma, Microbiol. Spectrum 11 (6): e0246823, 12 (2023); *Distoseptispora longispora* H.Y. Song & D.M. Hu, Mycotaxon 135 (3): 516 (2020); *Distoseptispora zhejiangensis* M.G. Liao & Jian Ma, J. Fungi 11 (7, no. 494): 15 (2025).

##### Description.

See Su et al. 2016.

##### Notes.

**Figure 6.**
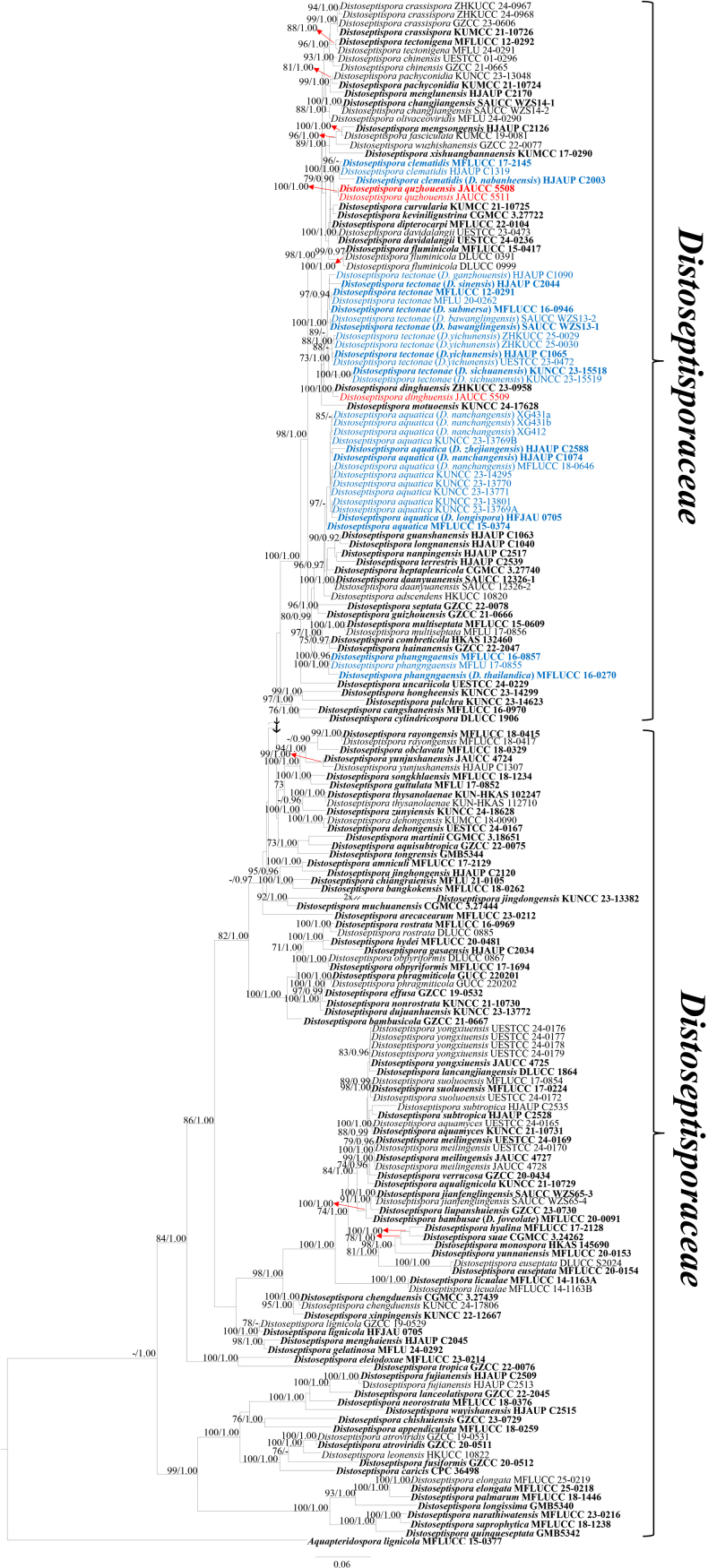
Phylogram generated from maximum likelihood analysis based on combined ITS, LSU, *RPB*2, and *TEF*1-α sequence data of 177 taxa, which comprised 3,529 characters. The best-scoring RAxML tree with a final likelihood value of –49751.839807 is presented. The matrix contained 2,097 distinct alignment patterns, with 26.04% of characters undetermined (gaps). Estimated base frequencies were as follows: A = 0.239507, C = 0.266440, G = 0.280282, T = 0.213772; substitution rates: AC = 1.416462, AG = 3.818017, AT = 1.460533, CG = 0.985947, CT = 7.354741, GT = 1.000000; gamma distribution shape parameter α = 0.318524. Bootstrap support values for ML of at least 70% and BYPP of at least 0.90 are indicated at the nodes as ML/BYPP. Type specimens are in bold, and the new species from the current study are indicated in bold red, and synonyms are indicated in blue. The tree is rooted to *Aquapteridospora lignicola* (MFLUCC 15–0377).

*Distoseptispora nanchangensis* was synonymized with *D. aquatica* by Shen et al. (2025) based on morphological and phylogenetic evidence. *Distoseptispora longispora* and *D. zhejiangensis* were introduced as new species solely on the basis of conidiophore and conidial size, despite minimal nucleotide divergence (Song et al. 2020; Liao et al. 2025a). However, in the phylogenetic tree (Fig. 6), *D. longispora* was grouped with *D. aquatica* strains, consistent with the results of Liao et al. (2025b), whereas *D. zhejiangensis* was grouped with *D. nanchangensis*, consistent with Liao et al. (2025a). *Distoseptispora longispora* and *D. zhejiangensis* show conidiophore and conidial morphologies similar to those of *D. aquatica*, with short, cylindrical conidiophores and obclavate, brown to yellowish-brown conidia. While they differ only in conidial size and septation, they display minor molecular differences (Song et al. 2020; Liao et al. 2025a; see Table 1 for details). Therefore, *D. longispora* and *D. zhejiangensis* are treated as synonyms of *D. aquatica*.

**Table 1.** Comparison of morphological characteristics and base-pair differences of ex-type strains between *Distoseptispora aquatica* (MFLUCC 15-0374, ex-type/KUNCC 23-14295, supplemental protein gene sequence) and other related type species. Sequence lengths are counted without gaps.

Species	Conidiophores	Conidia	ITS (bp)	LSU (bp)	TEF1-α (bp)	*RPB2* (bp)
*D. aquatica* (MFLUCC 15-0374)	Cylindrical, dark brown, 12–28 µm × 8–9.5 µm, 2–3-septate	Obclavate, elongated, dark brown with bluish green to malachite green tinge, 110–157 × 14–17, 15–28-distoseptate	–	–	–	–
*D. longispora* (HFJAU 0705)	Cylindrical, brown to dark brown, 17–37 x 6–10 um, 2–4-septate	Obclavate, elongated, brown to yellowish brown, 189–297 × 16–23 µm, 31–56-distoseptate	5/503 (0.9%)	6/834 (0.7%)	NA	NA
*D. nanchangensis* (HJAUP C1074)	Cylindrical, brown to dark brown, 18–76 × 6–8 µm, 2–5-septate	Obclavate, brown to dark brown, 149–293 × 11–18 µm, (17–)21–43-distoseptate	2/582 (0.3%)	0/524 (0%)	4/877 (0.4%)	6/753 (0.7%)
*D. zhejiangensis* (HJAUP C2588)	Cylindrical, brown, 16–56 × 13.5–20 µm, 1–3-septate	Obclavate, brown, 128–262 × 18.7–26.7 µm, 13–20-distoseptate	2/587 (0.3%)	1/543 (0.1%)	4/877 (0.4%)	NA

“NA” indicates the sequence unavailability.

#### Distoseptispora clematidis

Taxon classificationAnimaliaDistoseptisporalesDistoseptisporaceae

Phukhams., M.V. de Bult & K.D. Hyde, Fungal Diversity 102: 168 (2020)

626D2AAE-9023-509B-B172-C6DC30E96EBC

557301

##### Synonym.

*Distoseptispora nabanheensis* Jing W. Liu, X.G. Zhang & Jian Ma, J. Fungi 9 (4, no. 470): 7 (2023).

##### Description.

See Phukhamsakda et al. 2020.

##### Notes.

The phylogenetic analysis of combined ITS, LSU, *TEF*1-α, and *RPB*2 sequences showed that *Distoseptispora nabanheensis* clustered with *D. clematidis* with 100% ML and 1.00 BYPP statistical support (Fig. 6). Based on nucleotide comparisons, *D. nabanheensis* (HJAUP C2003, ex-type) differs from *D. clematidis* (MFLUCC 17–2145, ex-type) by 2/553 bp (0.3%, without gaps) in ITS, 2/465 bp (0.4%, without gaps) in LSU, and 3/931 bp (0.3%, without gaps) in *TEF*1-α. Morphologically, *D. nabanheensis* (HJAUP M2003, holotype) is similar to *D. clematidis* (MFLU 17–1501, holotype) in having obclavate conidia, and the conidial size and number of distosepta are also similar (120–210 × 12–20 μm vs. 102–214.5 × (7–)11–14.5 µm) and (18–31 vs. 28–35), respectively (Phukhamsakda et al. 2020; Liu et al. 2023). Based on the highly similar conidial morphology and molecular sequence data, *D. nabanheensis* is treated as a synonym of *D. clematidis*.

#### Distoseptispora phangngaensis

Taxon classificationAnimaliaDistoseptisporalesDistoseptisporaceae

Jing Yang, Maharachch. & K.D. Hyde, Mycol. Progr. 17 (5): 609 (2017)

E51C6208-3AF8-56BB-B78C-0EAE5BD0D074

821272

##### Synonym.

*Distoseptispora thailandica* Tibpromma & K.D. Hyde, Fungal Diversity 93: 79 (2018).

##### Description.

See Yang et al. 2017.

##### Notes.

The phylogenetic analysis of combined ITS, LSU, *TEF*1-α, and *RPB*2 sequences showed that *Distoseptispora thailandica* clustered with *D. phangngaensis* with 100% ML and 1.00 BYPP statistical support (Fig. 6), and the results are consistent with those of Du et al. (2025), Dong et al. (2025), Liao et al. (2025b), Liu et al. (2025a), and Shen et al. (2025). Based on nucleotide comparisons, *D. thailandica* (MFLUCC 16-0270, ex-type) differs from *D. phangngaensis* (MFLUCC 16–0857, ex-type) by 0/507 bp (0%, without gaps) in ITS, 3/799 bp (0.3%, without gaps) in LSU, and 1/929 bp (0.1%, without gaps) in *TEF*1-α. *Distoseptispora thailandica* and *D. phangngaensis* share similar conidiophore morphology (brown and cylindrical) and dimensions (15–26 × 3–6 μm vs. 18–30(–40) × 4–6 μm). Both species also possess obclavate, dark brown conidia, although they differ in conidial size (130–230 × 13.5–17 μm vs. 165–350 × 14–19 μm) (Yang et al. 2017; Tibpromma et al. 2018). Both species were collected in Thailand: *D. thailandica* was isolated from terrestrial habitats (Tibpromma et al. 2018), whereas *D. phangngaensis* was isolated from freshwater habitats (Yang et al. 2017). Based on highly similar morphology and molecular sequence data, *D. thailandica* is treated as a synonym of *D. phangngaensis*.

#### Distoseptispora tectonae

Taxon classificationAnimaliaDistoseptisporalesDistoseptisporaceae

Doilom & K.D. Hyde, Fungal Diversity 80: 222 (2016)

A3C27D82-90C6-5904-9833-BF1438F1ABC3

552223

##### Synonym.

*Distoseptispora bawanglingensis* W.W. Liu, C.Z. Yin, X.G. Zhang & Shi Wang, J. Fungi 11 (9, no. 667): 7 (2025); *Distoseptispora ganzhouensis* M.G. Liao & Jian Ma, MycoKeys 113: 42 (2025); *Distoseptispora sichuanensis* L.S. Dissan., K.D. Hyde & J.C. Kang, Mycosphere 15 (1): 1687 (2024); *Distoseptispora sinensis* Jing W. Liu, X.G. Zhang & Jian Ma, J. Fungi 9 (4, no. 470): 8 (2023); *Distoseptispora submersa* Doilom & K.D. Hyde, Fungal Diversity 80: 222 (2016); *Distoseptispora yichunensis* Y.F. Hu & Jian Ma, Microbiol. Spectrum 11 (6): e0246823, 13 (2023).

##### Description.

See Hyde et al. 2016b.

##### Notes.

*Distoseptispora submersa* was synonymized with *D. tectonae* by Dong et al. (2021) based on phylogenetic analysis. In addition, they found that the conidiophore dimensions can vary in *D. tectonae* (Dong et al. 2021). In the phylogenetic tree, *D. bawanglingensis*, *D. ganzhouensis*, *D. sichuanensis*, *D. sinensis*, *D. submersa*, and *D. yichunensis* formed a sister clade with *D. tectonae* (Fig. 6), and the results are consistent with Du et al. (2025), Dong et al. (2025), Liao et al. (2025a), Liu et al. (2025a), and Shen et al. (2025). Morphologically, all these species share cylindrical, pale brown to dark brown conidiophores and obclavate, brown, distoseptate conidia, differing only in conidial size. However, several studies have indicated that conidial size alone may not be a reliable taxonomic criterion at the species level (Yang et al. 2018, 2021; Shen et al. 2024, 2025). In addition, a nucleotide comparison of *D. bawanglingensis*, *D. ganzhouensis*, *D. sichuanensis*, *D. sinensis*, *D. submersa*, and *D. yichunensis* with the ex-type *D. tectonae* (for details, please see Table 2) shows minimal nucleotide divergence among them, which suggests that they are conspecific. Therefore, *D. bawanglingensis*, *D. ganzhouensis*, *D. sichuanensis*, *D. sinensis*, and *D. yichunensis* are treated as synonyms of *D. tectonae*.

**Table 2.** Comparison of morphological characteristics and base-pair differences among ex-type strains of *Distoseptispora tectonae* (MFLUCC 12-0291) and related species; sequence lengths are counted without gaps.

Species	Conidiophores	Conidia	ITS (bp)	LSU (bp)	TEF1-α (bp)	*RPB2* (bp)
*D. tectonae* (MFLUCC 12-0291)	Cylindrical, pale brown to dark brown, up to 40 µm long, 4–6 µm wide, 2–4-septate	Cylindric-obclavate, elongate, brown, (90–)130–140(–170) × (11–)13–14(–16) µm, 20–28-distoseptate	–	–	–	–
*D. bawanglingensis* (SAUCC WZS13‐1)	Cylindrical, dark brown, 14–40 × 3.5–6.7 μm, 2–4-septate	Obclavate, brown to pale brown, 20–207 × 8–14 µm, 3–45-distoseptate	4/552 (0.7%)	3/826 (0.3%)	3/888 (0.2%)	2/898 (0.2%)
*D. ganzhouensis* (HJAUP C1090)	Cylindrical, brown to dark brown, 54–93 × 4.5–7 µm, 5–10-septate	Obclavate, pale brown to brown, 59–139 × 12–167 µm, 10–23-distoseptate	4/516 (0.7%)	4/559 (0.7%)	3/935 (0.3%)	NA
*D. sichuanensis* (KUNCC 23-15518)	Cylindrical, yellowish brown to dark brown, 15–25 × 4–6 μm, 1–4- septate	obclavate, elongated, yellowish brown to brown, 80–145 × 6–17 µm, 12–20-distoseptate	3/515 (0.5%)	2/745 (0.2%)	7/846 (0.8%)	NA
*D. sinensis* (HJAUP C2044)	Cylindrical, brown to dark brown, 24–57 × 4–7 μm, 2–5-septate	obclavate, brown to dark brown, 40–107(–137) × 10–12 µm, 10–25-distoseptate	5/558 (0.8%)	2/559 (0.3%)	4/930 (0.4%)	NA
*D. submersa* (MFLUCC 16‐0946)	Cylindrical, brown to dark brown, 55–73 μm × 7–9 μm, 4–5-septate	obclavate, lanceolate or obpyriform, brown, 95–123 × 15–19 μm, 17–23(28)-distoseptate	2/492 (0.4%)	2/821 (0.2%)	2/866 (0.2%)	3/763 (0.3%)
*D. yichunensis* (HJAUP C1065)	Cylindrical, brown to dark brown, 18–53 × 5–7 µm, 3–6-septate	obclavate, pale brown to brown, 114–273 × 12–17 µm, (14–)22–35-distoseptate	5/570 (0.8%)	2/559 (0.4%)	3/927 (0.3%)	7/830 (0.8%)

“NA” indicates the sequence unavailability.

#### Distoseptispora dinghuensis

Taxon classificationAnimaliaDistoseptisporalesDistoseptisporaceae

Yun H. Yang, K.D. Hyde & Manawas., Fungal Diversity 134: 301 (2025)

B867B87A-4AC8-5829-95D7-88E695063470

902258

Fig. 7

##### Description.

***Saprobic*** on decaying submerged wood in freshwater habitats. **Teleomorph**: Undetermined. **Anamorph**: Hyphomycetous. Colonies effuse, black, hairy. ***Mycelium*** partly superficial, composed of hyaline to pale brown, septate, branched hyphae. ***Conidiophores*** 5–34.5 × 4.5–7 µm (x̄ = 14.5 × 5.5 µm, *n* = 20), 0–3-septate, macronematous, mononematous, cylindrical, dark brown, rounded at the apex. ***Conidiogenous cells*** 2.5–8.5 × 3.5–6 µm (x̄ = 5.5 × 5 µm, *n* = 20), macronematous, mononematous, integrated, terminal, monoblastic, brown, smooth-walled, unbranched. ***Conidia*** 43.5–321 × 7.5–14.5 µm (x̄ = 228.5 × 11 µm, *n* = 50), acrogenous, solitary, dry, long cylindrical or obclavate, oblong or elongated, straight or curved, 10–48-distoseptate, rounded at the apex, truncate at the base, brown to dark brown, slightly paler toward the apex, smooth, thick-walled.

##### Cultural characteristics.

Conidia germinating on PDA within 24 h and germ tubes produced from the conidial base. Colonies on PDA reaching 17 mm diam in 16 days at 25 °C, in natural light, with fluffy, dense, thin olivaceous mycelium in the centre, becoming sparse and paler at the entire margin; reverse dark brown, pale brown at the smooth margin.

##### Material examined.

CHINA • Jiangxi Province: Ji’an City, Wanan County, Shunfeng Village, 26.19°N, 115.01°E, 241 m, on submerged unidentified wood in freshwater, 30 June 2022, WM He, hwm023 (HFJAU10090); living culture JAUCC 5509.

##### Known hosts.

On a dead trunk of *Litchi chinensis* (Dong et al. 2025), on submerged decaying unidentified wood (this study).

##### Known distribution.

China, Guangdong Province (Dong et al. 2025), Jiangxi Province (this study).

##### Notes.

**Figure 7.**
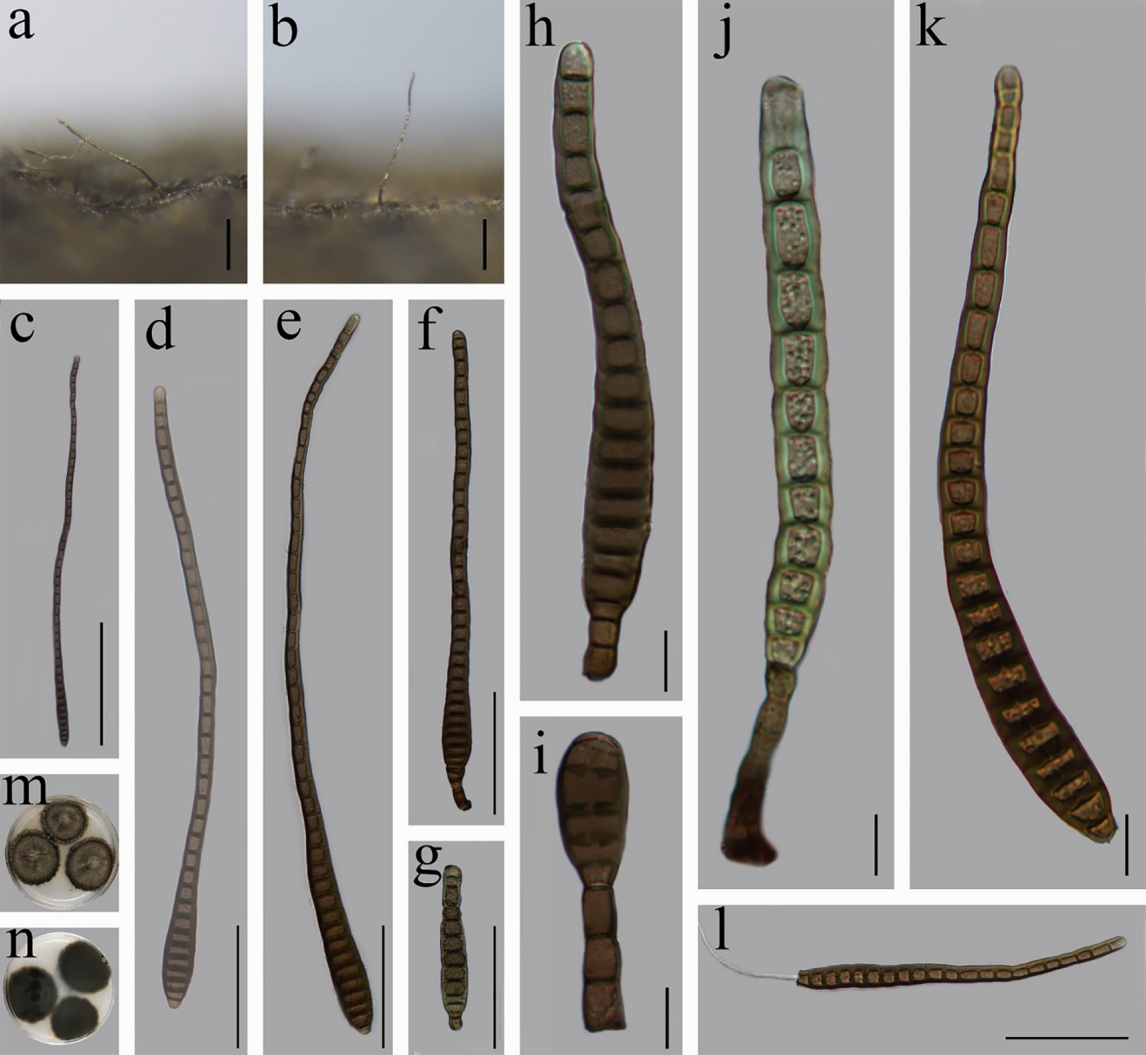
*Distoseptispora dinghuensis* (HFJAU10090, new host record). **a, b** Colonies on natural substrate. **c–e, k** Conidia. **f–j** Conidiophores with conidia. **l** Germinated conidium. **m, n** Culture on PDA from above and reverse. Scale bars: 100 µm (**a–c**); 50 µm (**d–g, l**); 10 µm (**h–k**).

*Distoseptispora dinghuensis* was introduced by Dong et al. (2025) from terrestrial habitats in Guangdong, China. The collection was obtained from freshwater habitats in Jiangxi, China, and shares a sister branch with *D. dinghuensis* (ZHKUCC 23-0958, ex-type) with 100% ML and 1.00 BYPP statistical support (Fig. 6). Morphologically, the collection (HFJAU10090) also aligns well with the description of the holotype *D. dinghuensis* (MHZU 23-0234, holotype) in its short and hairy conidiophores and long cylindrical or obclavate conidia (Dong et al. 2025; Fig. 7). The nucleotide comparison between the isolate (JAUCC 5509) and the type strain of *D. dinghuensis* (ZHKUCC 23-0958) shows that they have identical ITS, LSU, and *TEF*1-α sequences and only a few nucleotide differences of 0.8% (7/880 bp, without gaps) within the *RPB*2 sequence. Therefore, the new isolate is identified as *D. dinghuensis* and reported for the first time from freshwater habitats.

#### Distoseptispora quzhouensis

Taxon classificationAnimaliaDistoseptisporalesDistoseptisporaceae

Z. J. Zhai, Z. H. Jin & D. M. Hu sp. nov.

B357BBAE-AA95-5DF5-9A61-61C01DB3ECC7

848589

Fig. 8

##### Holotype.

HFJAU10089.

##### Etymology.

The species epithet refers to the type location “Quzhou,” China.

##### Diagnosis.

Differs from *D. clematidis* by the dark olivaveous to dark brown conidia, 11–64(–98) distoseptate.

##### Description.

***Saprobic*** on decaying submerged wood in freshwater habitats. **Teleomorph**: Undetermined. **Anamorph**: Hyphomycetous. ***Colonies*** effuse, black, hairy. ***Mycelium*** partly superficial on the substrate, composed of hyaline to pale brown, septate, branched hyphae. ***Conidiophores*** (13–)24–57.5 × 5.5–9 µm (x̄ = 39.5 × 7 µm, *n* = 20), (1–)2–5-septate, macronematous, mononematous, cylindrical, dark brown, rounded at the apex. ***Conidiogenous cells*** 5–10 × 5–7 µm (x̄ = 8 × 6 µm, *n* = 15), integrated, terminal, monoblastic, brown, smooth. ***Conidia*** 54–356(–541) × 12–20.5 µm (x̄ = 179 × 16 µm, *n* = 50), acrogenous, solitary, dry, obclavate, rarely oblong, elongated, mostly curved, dark olivaceous to dark brown, 11–64(–98)-distoseptate, rounded at the apex, truncate at the base, smooth, thick-walled, with granulate.

##### Cultural characteristics.

Conidia germinating on PDA within 24 h and germ tubes produced from the conidial apices. Colonies on PDA reaching 17 mm diam in 16 days at 25 °C, in natural light, with fluffy, dense, thin olivaceous mycelium in the center, becoming sparse and paler at the entire margin; reverse dark brown, pale brown at the smooth margin.

##### Material examined.

CHINA • Zhejiang Province: Jiangshan City, Quzhou, 28.310°N, 118.560°E, 559 m, on submerged unidentified wood in freshwater, 17 October 2021, ZH Jin, JS007 (HFJAU10089, holotype); ex-type living culture JAUCC 5508, other living culture JAUCC 5511.

##### Notes.

**Figure 8.**
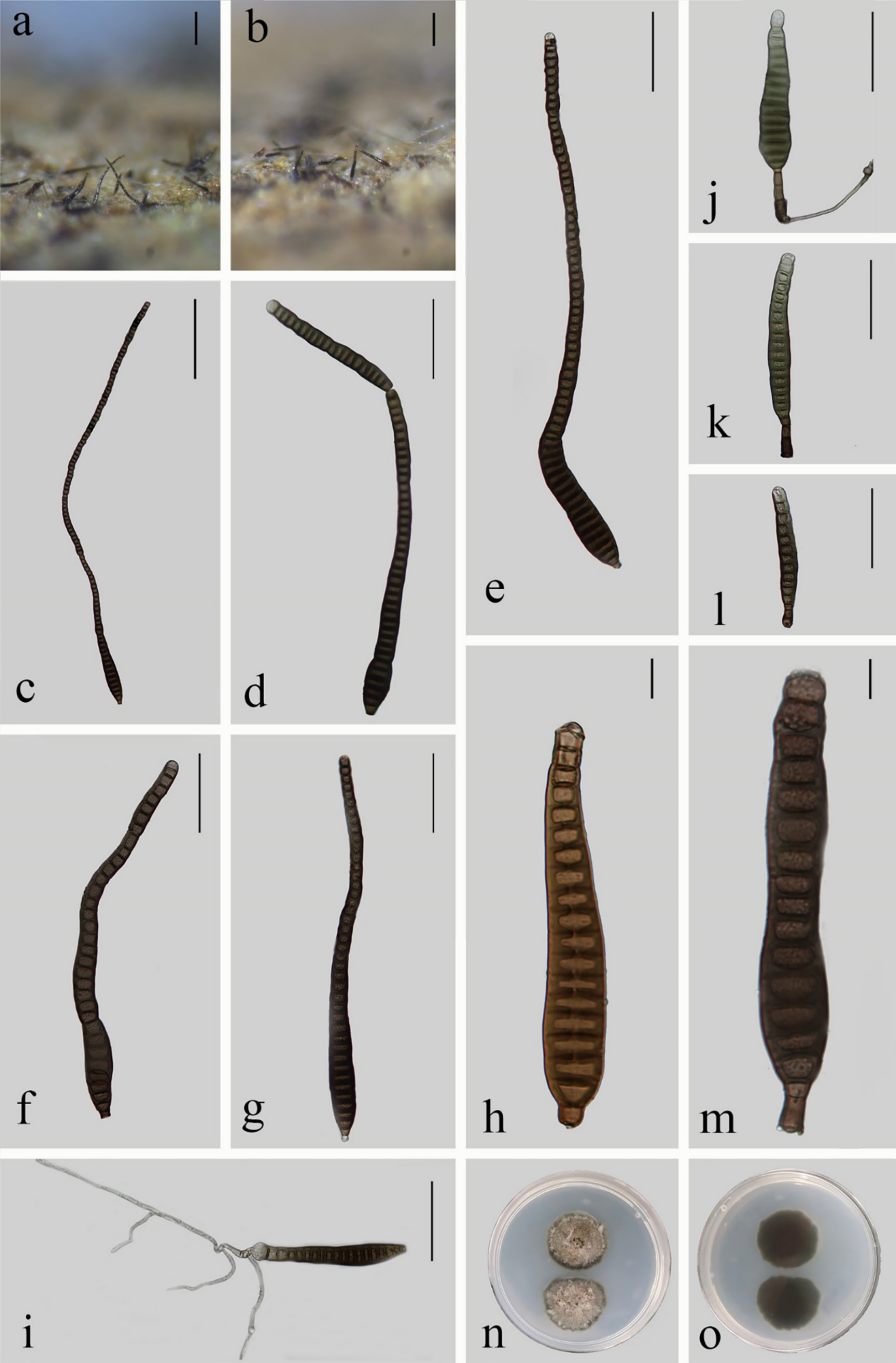
*Distoseptispora quzhouensis* (HFJAU10089, holotype). **a, b** Colonies on natural substrate. **c–h** Conidia. **j–m** Conidiophores with conidia. **i** Germinated conidium. **n, o** Culture on PDA from above and reverse. Scale bars: 100 µm (**a–c**); 50 µm (**d–g, i–l**); 10 µm (**h, m**).

Based on phylogenetic analyses, *Distoseptispora quzhouensis* (JAUCC 5508, ex-type, JAUCC 5511) formed an independent branch under *D. clematidis* (HJAUP C1319, HJAUP C2003, and MFLUCC 17–2145, ex-type) with 79% ML and 0.90 BYPP statistical support (Fig. 6). The sequence similarity between the two strains (JAUCC 5508 and JAUCC 5511) is above 99% for the analyzed genes, confirming their conspecificity. Morphologically, *D. quzhouensis* (HFJAU10089, holotype) is similar to *D. clematidis* (MFLU 17-1501, holotype) in having acrogenous, solitary, obclavate, elongated, and distoseptate conidia (Phukhamsakda et al. 2020). However, *D. quzhouensis* can be distinguished from the holotype of *D. clematidis* by the dark olivaceous to dark brown conidia and 11–64(–98)-distoseptation, while *D. clematidis* has brown conidia with a green tinge and 28–35-distoseptation (Phukhamsakda et al. 2020; Fig. 8). In addition, pairwise nucleotide comparisons of *D. quzhouensis* (JAUCC 5508, ex-type) and *D. clematidis* (MFLUCC 17–2145, ex-type) showed 3% nucleotide differences in ITS (15/472 bp, without gaps), 0.4% nucleotide differences in LSU (3/726 bp, without gaps), and 0.5% nucleotide differences in *TEF*1-α (4/699 bp, without gaps). Therefore, *D. quzhouensis* is introduced here as a new species.

### *Diaporthomycetidae* families *incertae sedis*

#### Junewangiaceae

Taxon classificationAnimaliaDistoseptisporalesJunewangiaceae

J.W. Xia & X.G. Zhang, Scientific Reports 7 (no. 7888): 12 (2017)

399316B5-18AD-5A5C-AA6C-4360633CDA1D

##### Notes.

*Junewangiaceae* was introduced by Xia et al. (2017) to accommodate the *Acrodictys*-like genus and *Junewangia*, known as the type. Subsequently, phylogenetic analysis showed that *Dictyosporella*, *Junewangia*, and *Sporidesmiella* clustered within *Junewangiaceae* (Dong et al. 2021; Hyde et al. 2023). Additionally, *Jennwenomyces* was proposed to replace *Belemnospora navicularis* with *Jennwenomyces navicularis*, which was described and illustrated based on two specimens collected from decaying culms submerged in freshwater streams (Goh and Kuo 2020). Although phylogenetic analysis placed *Jennwenomyces* within *Junewangiaceae*, Goh and Kuo (2020) classified it as a genus in *Sordariomycetes*, *incertae sedis*. There are currently four accepted genera in *Junewangiaceae* that occur primarily as saprobes on a variety of plant debris in terrestrial and freshwater habitats (Hyde et al. 2024). However, the phylogenetic boundaries between *Dictyosporella*, *Junewangia*, *Jennwenomyces*, and *Sporidesmiella* remain unclear (Goh and Kuo 2020; Hyde et al. 2023), but they exhibit distinct morphological differences (Wang et al. 2025a). Therefore, relevant research should continue to be conducted on this family to clarify their phylogenetic relationships and establish stable generic boundaries.

#### Sporidesmiella

Taxon classificationAnimaliaDistoseptisporalesMelanommataceae

P.M. Kirk, Trans. Br. mycol. Soc. 79(3): 479 (1982)

15BF7191-EBC9-54E3-ABCC-22402F7C7745

##### Notes.

Kirk (1982) established *Sporidesmiella* to accommodate two species and other species previously referred to *Sporidesmium* and *Endophragmia* (Kirk 1982). The taxonomic status was confirmed by Luo et al. (2019), who placed it in *Junewangiaceae* based on multi-locus phylogenetic analysis. The genus is characterized by distoseptate, cuneate to obovoid conidia with a truncated base and schizotypic production from terminally annellate conidiophores (Kirk 1982; Yanna et al. 2001; Ma et al. 2015; Xu et al. 2025; Shen et al. 2025). *Sporidesmiella* has a wide geographical distribution and is reported from terrestrial and freshwater habitats, primarily as saprobic on a variety of plants (Wu 2005; Luo et al. 2019; Crous et al. 2021b; Dong et al. 2021; Shen et al. 2025). In this study, a new species and a new record of *Sporidesmiella*, collected from submerged wood in freshwater in Jiangxi Province, are introduced.

#### Sporidesmiella saprophytica

Taxon classificationAnimaliaDistoseptisporalesMelanommataceae

S.P. Zheng, D.M. Hu & H.Y. Song sp. nov

9C6E2032-C567-5731-A41F-9C691E6A0F95

903575

Fig. 10

##### Etymology.

The epithet “saprophytica” refers to the saprobic life mode of the fungus.

##### Diagnosis.

Differs from *S. obovoidispora* by larger conidiogenous cells (26–38 × 2.5–4 μm vs. 3.5–8.5 × 2.5–4 µm), and the different color of conidia (obovoid, hyaline to light brown vs. clavate, pale brown to reddish brown).

##### Holotype.

HFJAU10499.

##### Description.

***Saprobic*** on the decaying wood submerged in freshwater habitats. **Teleomorph**: Undetermined. **Anamorph**: Hyphomycetous. ***Colonies*** grow on the surface of the natural substrate, effuse, hairy, and gregarious, with pale brown masses on the apex of conidiophores. ***Mycelium*** partly immersed in the substrate, composed of unbranched, septate, smooth, and pale brown hyphae. ***Conidiophores*** 121–188 μm long and 3.5–5 μm wide, macronematous, mononematous, solitary, erect, cylindrical, straight or slightly flexuous, brown, gradually paler and becoming pale brown towards the apex, unbranched, septate, smooth. ***Conidiogenous cells*** 26–38 μm long and 2.5–4 μm wide, holoblastic, polyblastic, integrated, terminal, pale brown to subhyaline, cylindrical, smooth. ***Conidia*** 23–29 × 11–14 μm, (x̄ = 26 × 12 μm, *n* = 30), acrogenous, solitary, obovoid, (3)–4-distoseptate, hyaline to light brown, obtusely conic or rounded at the apex, with a truncate base, thin-walled, smooth, with oil guttules.

##### Culture characteristics.

Conidia germinating on PDA within 24 h. Colonies grew on PDA, reaching 12 mm in 40 days at 25 °C, circular, sparse, with dense and soft mycelium, white in the middle, white to light pink in the outer ring, reddish-brown from below, entire at the edge.

##### Material examined.

CHINA • Jiangxi Province: Nanchang City, Anyi County, Zhuoluo Village, 28°92'23"N, 115°51'79"E, 49.8 m, 13 Oct 2024, on submerged unidentified wood in freshwater, S.P. Zheng, zsp 82 (HFJAU10678, holotype); ex-type living culture JAUCC 7440, other living culture JAUCC 7441.

##### Notes.

**Figure 9.**
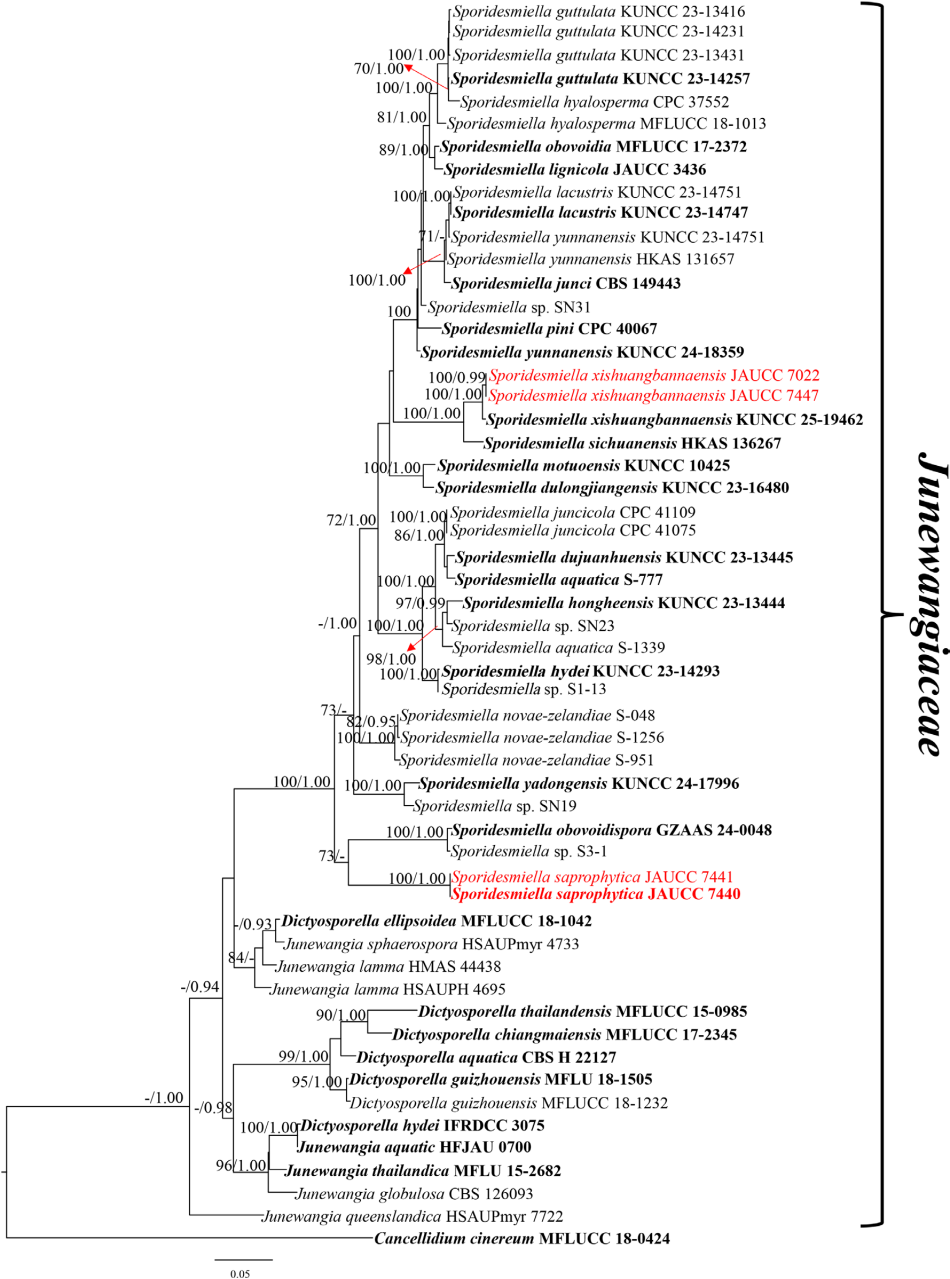
Phylogram generated from maximum likelihood analysis based on combined ITS, LSU, *RPB*2, and *TEF*1-α sequence data of 55 taxa, which comprised 3,536 characters. The best-scoring RAxML tree with a final likelihood value of –17912.553286 is presented. The matrix contained 1,174 distinct alignment patterns, with 41.62% of characters undetermined (gaps). Estimated base frequencies were as follows: A = 0.254658, C = 0.244861, G = 0.266615, T = 0.233866; substitution rates: AC = 1.011447, AG = 3.632044, AT = 2.025971, CG = 0.554291, CT = 6.972937, GT = 1.000000; gamma distribution shape parameter α = 0.177518. Bootstrap support values for ML of at least 70% and BYPP of at least 0.90 are indicated at the nodes as ML/BYPP. Type specimens are in bold, and the new species from the current study are indicated in bold red. The tree is rooted to *Cancellidium cinereum* (MFLUCC 18–0424).

**Figure 10.**
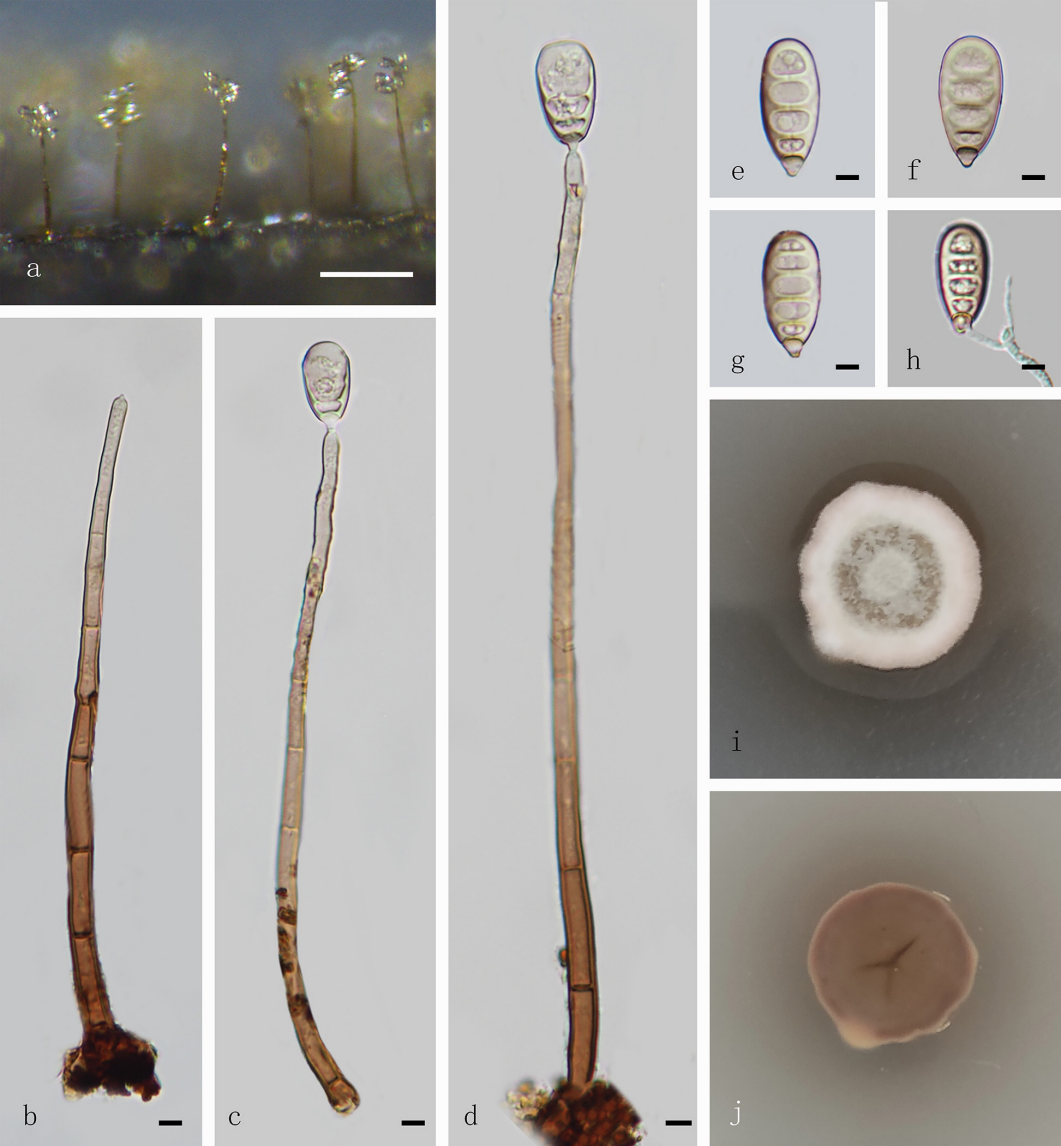
*Sporidesmiella saprophytica* (HFJAU10678, holotype). **a** Colony on the natural substrate. **b–d** Conidiophores, conidiogenous cells, and conidia. **e–g** Conidia. **h** Germinated conidium. **i, j** Colony on PDA. Scale bars: 100 μm (**a**); 10 μm (**b–h**).

In the multi-locus phylogenetic tree based on ITS, LSU, *TEF*1-α, and *RPB*2, *Sporidesmiella saprophytica* (JAUCC 7440, ex-type, and JAUCC 7441) formed a separate clade under *S. obovoidispora* (GZAAS 24-0048, holotype) and *Sporidesmiella* sp. (S3-1) with statistical support (73% ML) (Fig. 9). The sequence similarity between the two strains (JAUCC 7440 and JAUCC 7441) is above 99% for the analyzed genes, confirming their conspecificity. Based on pairwise nucleotide comparisons, *S. saprophytica* (JAUCC 7440, ex-type) diverges from *S. obovoidispora* (GZAAS 24-0048) by 59/435 bp (13%, without gaps) for ITS and 45/811 bp (5.5%, without gaps) for LSU; it also diverges from *Sporidesmiella* sp. (S3-1) by 60/475 bp (12%, without gaps) for ITS and 45/808 bp (5.5%, without gaps) for LSU, and the two species lack *TEF*1-α and *RPB*2. Morphologically, *S. saprophytica* is similar to *S. obovoidispora* (GZAAS 24-0048, holotype) and also conforms to *Sporidesmiella*, characterized by brown, smooth, septate, unbranched conidiophores; monoblastic, holoblastic, terminal conidiogenous cells; and obovoid conidia (Kirk 1982; Yanna et al. 2001; Ma et al. 2015; Fig. 10). However, *S. saprophytica* (HFJAU10678, holotype) can be distinguished from *S. obovoidispora* by having differently septate conidiophores, larger conidiogenous cells (26–38 × 2.5–4 μm vs. 3.5–8.5 × 2.5–4 µm), and differently colored conidia (obovoid, hyaline to light brown vs. clavate, pale brown to reddish brown) (Liu et al. 2024a; Fig. 10). Based on molecular data and morphological features, *S. saprophytica* is identified as a new species of *Sporidesmiella*.

#### Sporidesmiella xishuangbannaensis

Taxon classificationAnimaliaDistoseptisporalesMelanommataceae

Z.Y. Wang, K.D. Hyde & Q. Zhao (2026, in press)

29EA9EC9-42F7-5DFA-8096-123EDBC5D52B

904328

Fig. 11

##### Description.

***Saprobic*** on rotting ***Phragmites*** stems submerged in freshwater habitats. **Teleomorph**: Undetermined. **Anamorph**: Hyphomycetous. ***Colonies*** superficial on the substrate, effuse, hairy, gregarious, with yellow-brown masses aggregated on the apex of conidiophores. ***Mycelium*** mostly immersed in the substrate, partly superficial, composed of branched, septate, smooth, thick-walled, pale brown to brown hyphae. ***Conidiophores*** 64–109 × 3.2–4.8 μm (x̄ = 91.6 × 4 μm, *n* = 15), macronematous, mononematous, solitary, erect, straight or slightly flexuous, cylindrical, brown, paler towards the apex, unbranched, septate, thick-walled, smooth to slightly verrucose. ***Conidiogenous cells*** 10.7–18.8 × 3.2–4.5 μm, (x̄ = 14.8 × 3.9 μm, *n* = 15), holoblastic, polyblastic, integrated, terminal, cylindrical, subhyaline to pale brown, with percurrent or sympodial proliferations. ***Conidia*** 10.7–18.8 × 3.2–4.5 μm, (x̄ = 14.8 × 3.9 μm, *n* = 15), acrogenous, dry, smooth, thick-walled, broadly clavate to obovoid, (3)–4-distoseptate, pale brown, base truncate, apex obtuse, guttulate, thick, and smooth-walled.

##### Culture characteristics.

Conidia germinating on PDA within 24 h. Colonies grew on PDA, reaching 25 mm in 30 days at 25 °C, nearly circular, dry, rough, with gray floccose mycelium, slightly raised in the middle, reddish-brown from above and below, entire at the edge.

##### Material examined.

CHINA • Jiangxi Province: Shangrao City, Tongbo Mountain Scenic spot, 28°23'58"N, 118°28'28"E, 235.6 m, on submerged rotting *Phragmites* stems in freshwater, 18 Feb 2024, C.Y. Xu, xcy 134 (HFJAU 10425); living culture JAUCC 7022 and JAUCC 7447.

##### Known hosts.

Dead stems submerged in a freshwater stream (Qian et al. 2026, in press), on rotting *Phragmites* stems submerged in freshwater (this study).

##### Known distribution.

China, Yunnan Province (Qian et al. 2026, in press), Jiangxi Province (this study).

##### Notes.

**Figure 11.**
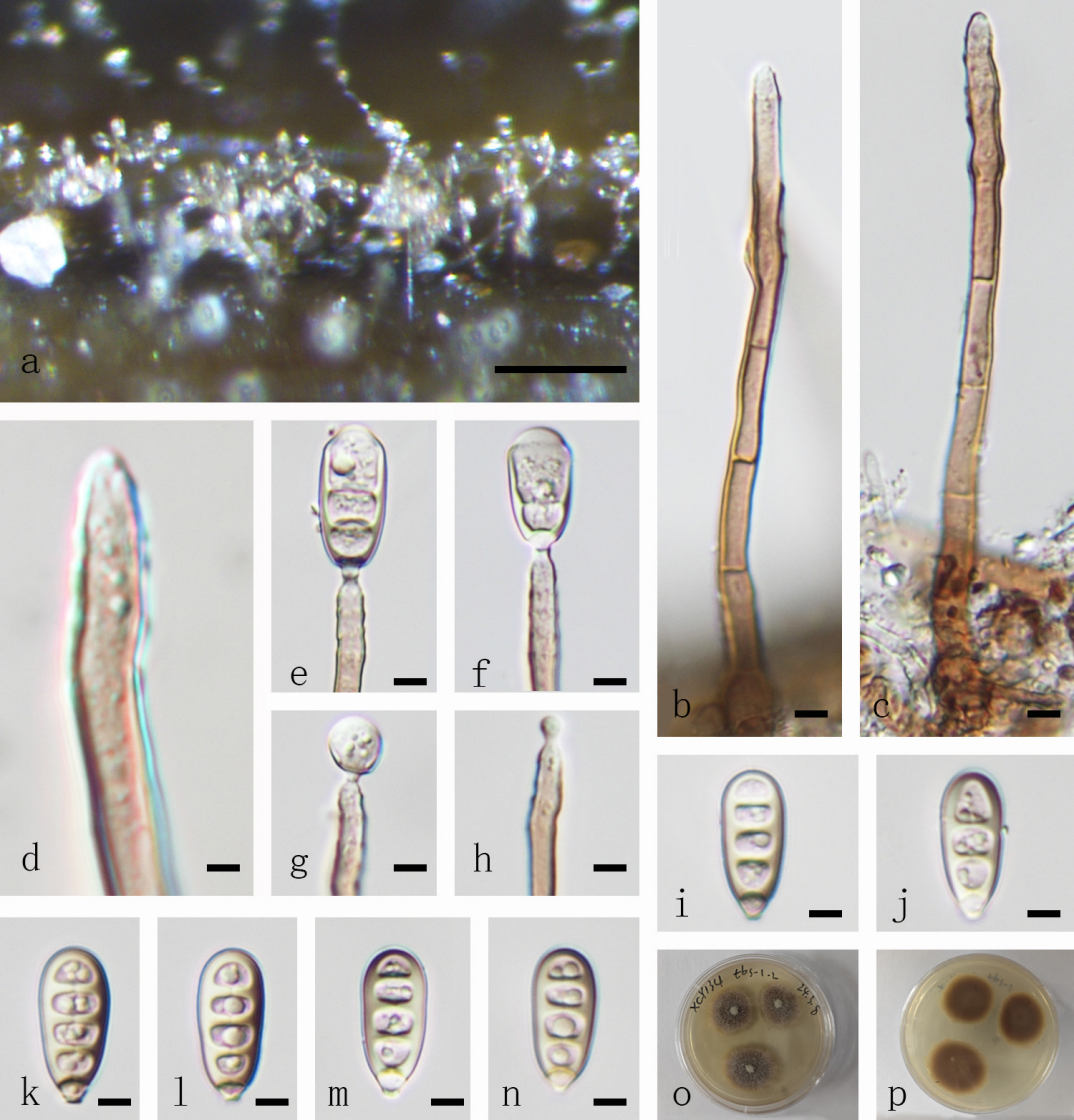
*Sporidesmiella xishuangbannaensis* (HFJAU10425, new host record). **a** Colonies on the natural substratum. **b, c** Conidiophores. **d** Conidiogenous cells. **e–h** Conidiogenous cells with conidia. **i–n** Conidia. **o, p** Colony on PDA from above and below. Scale bars: 100 μm (**a**); 5 μm (**e*–*n**); 2 μm (**d**).

Multi-locus phylogenetic analysis based on ITS, LSU, *TEF*1-α, and *RPB*2 sequences showed that the isolates (JAUCC 7022 and JAUCC 7447) formed a sister lineage to *Sporidesmiella xishuangbannaensis* (KUNCC 25–19462, ex-type) (100% ML, 1.00 BYPP; Fig. 9). Morphologically, the species is similar to the holotype *S. xishuangbannaensis* (HKAS 150447) in its clavate to obovoid, 3-distoseptate, subhyaline to pale brown conidia (Qian et al. 2026; Fig. 11). However, the conidiophores of the isolates are shorter than those of the holotype (64–109 × 3.2–4.8 μm vs. 200–285 × 2.5–3.5 µm). Based on nucleotide comparisons, the strain (JAUCC 7022) is nearly identical to *S. xishuangbannaensis* (KUNCC 25–19462, ex-type), with 6/500 bp (1.2%, without gaps) differences in ITS, and the LSU is identical. The holotype of *S. xishuangbannaensis* was collected in Yunnan Province from unidentified dead stems, whereas the new collection was obtained in Jiangxi Province from rotting *Phragmites* stems; hence, the isolate is introduced here as a new host record.

#### Papulosaceae

Taxon classificationAnimaliaDistoseptisporalesPapulosaceae

Winka & O.E. Erikss., Mycoscience 41(2): 102 (2000)

B8CDD179-CBFD-5F95-91E2-0838FC7730AF

##### Notes.

The family *Papulosaceae* was established for the monotypic marine genus *Papulosa* based on SSU sequence and morphological analyses (Winka and Eriksson 2000). Later, two freshwater genera, *Brunneosporella* and *Fluminicola*, were placed in this family by Maharachchikumbura et al. (2015). *Wongia* is the fourth genus placed in the family *Papulosaceae* based on multi-locus analyses (Khemmuk et al. 2016). There are only four genera accepted in *Papulosaceae* (Hyde et al. 2024), and most species in this family are reported from submerged wood in freshwater habitats and grow slowly in PDA culture (Ranghoo et al. 2001; Khemmuk et al. 2016).

#### Wongia

Taxon classificationAnimaliaDistoseptisporales

Khemmuk, Geering & R.G. Shivas, IMA Fungus 7(2): 249 (2016)

E1561FD5-96C5-547E-BC67-C3EAC5CE05A7

##### Notes.

*Wongia* was established to encompass two root-infecting species, with only the teleomorphs, *W. garrettii* (type) and *W. griffinii*, previously placed in *Magnaporthe* (Khemmuk et al. 2016). Based on multi-locus analyses, *Wongia* was placed in *Papulosaceae*, *Diaporthomycetidae* genera *incertae sedis*, *Sordariomycetes* (Maharachchikumbura et al. 2015; Khemmuk et al. 2016). The teleomorph of *Wongia* is morphologically distinguished by its unitunicate, cylindrical asci, which possess a refractive, non-amyloid apical ring, and cylindrical to fusiform, 3-septate ascospores that have dark brown middle cells and pale brown to subhyaline shorter distal cells (Wong et al. 2012; Khemmuk et al. 2016). The first anamorphic species, *W. aquatica*, was introduced from freshwater habitats by Luo et al. (2019) and is characterized by macronematous, mononematous, unbranched, septate, dark brown conidiophores; polyblastic, denticulate, integrated, terminal conidiogenous cells; and acrogenous, clavate-to-fusiform conidia (Luo et al. 2019). In this study, two new species with anamorphs, *W. lignicola* and *W. saprophytica*, are introduced from Jiangxi Province, China.

#### Wongia lignicola

Taxon classificationAnimaliaDistoseptisporales

C.Y. Xu, D.M. Hu & H.Y. Song sp. nov.

19939D7B-42E1-5740-96B9-0B672B59CB8C

903576

Fig. 13

##### Etymology.

The epithet “lignicola” refers to the wood-dwelling nature of the fungus.

##### Diagnosis.

Differs from *W. pallidopolaris* by having conspicuous guttules in conidia, while the conidia of *W. pallidopolaris* lack guttules, and the outer wall partly detaches from the conidium, the detached segments appear as apical or side pockets or wings.

##### Holotype.

HFJAU10529.

##### Description.

***Saprobic*** on decaying submerged wood in freshwater habitats. **Teleomorph**: Undetermined. **Anamorph**: Hyphomycetous. ***Colonies*** grow on the surface of the natural substrate, effuse, hairy, scattered, rarely in small groups, and brown. ***Mycelium*** immersed in the substrate, composed of branched, septate, smooth, and brown hyphae. ***Conidiophores*** 72–127 × 3.2–5.4 μm, (x̄ = 97 × 4.2 μm, *n* = 10), macronematous, mononematous, solitary, erect, straight or slightly flexuous, brown, gradually paler and becoming pale brown towards the apex, unbranched, septate, slightly constricted at the septa, thick-walled, smooth to slightly verrucose, apex inflated. ***Conidiogenous cells*** polyblastic, denticulate, terminal, pale brown to subhyaline. ***Conidia*** 20–30 × 3.4–6.6 μm, (x̄ = 25.3 × 4.8 μm, *n* = 35), acrogenous, solitary, ellipsoid to fusiform, or cylindrical, 1–3-septate, olive brown to brown, mostly rounded at both ends, smooth and thin-walled, guttulate.

##### Culture characteristics.

Conidia germinating on PDA within 24 h. Colonies grew on PDA, reaching 33 mm in 100 days at 25 °C, circular, rough surface, with dense mycelia, dry, umbonate in the middle from the side view, edge undulate, brown from above, medium dark brown, marginal hyphal density inconsistent, dark brown and light brown from below.

##### Material examined.

CHINA • Jiangxi Province: Shangrao City, Tongbo Mountain Scenic spot, 28°23'58"N, 118°28'28"E, 235.6 m, on submerged unidentified wood in freshwater, 18 Feb 2024, C.Y. Xu, xcy 176 (HFJAU10529, holotype); ex-type living culture JAUCC 7164, other living culture JAUCC 7445.

##### Notes.

**Figure 12.**
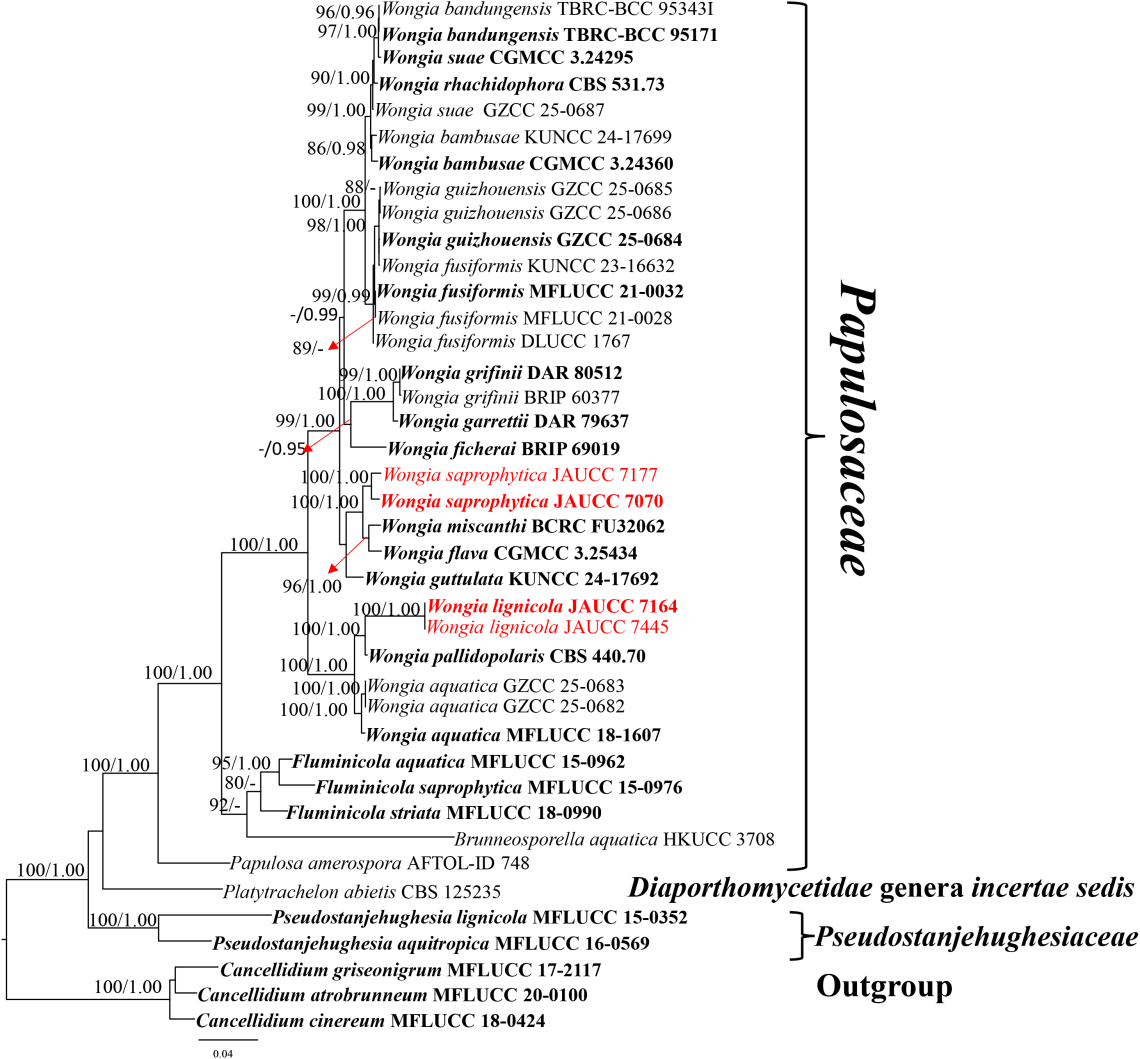
Phylogram generated from maximum likelihood analysis based on combined ITS, LSU, SSU, *RPB*2, and *TEF*1-α sequence data of 40 taxa, which comprised 3,953 characters. The best-scoring RAxML tree with a final likelihood value of –17125.130762 is presented. The matrix contained 1,062 distinct alignment patterns, with 25.59% of characters undetermined (gaps). Estimated base frequencies were as follows: A = 0.243169, C = 0.255267, G = 0.278303, T = 0.223261; substitution rates: AC = 1.030178, AG = 2.784585, AT = 1.149284, CG = 1.009131, CT = 6.869666, GT = 1.000000; gamma distribution shape parameter α = 0.176202. Bootstrap support values for ML of at least 70% and BYPP of at least 0.90 are indicated at the nodes as ML/BYPP. Type specimens are in bold, and the new species from the current study are indicated in bold red. The tree is rooted to *Cancellidium atrobrunneum* (MFLUCC 20-0100), *C. cinereum* (MFLUCC 18-0424), and *C. griseonigrum* (MFLUCC 17-2117).

**Figure 13.**
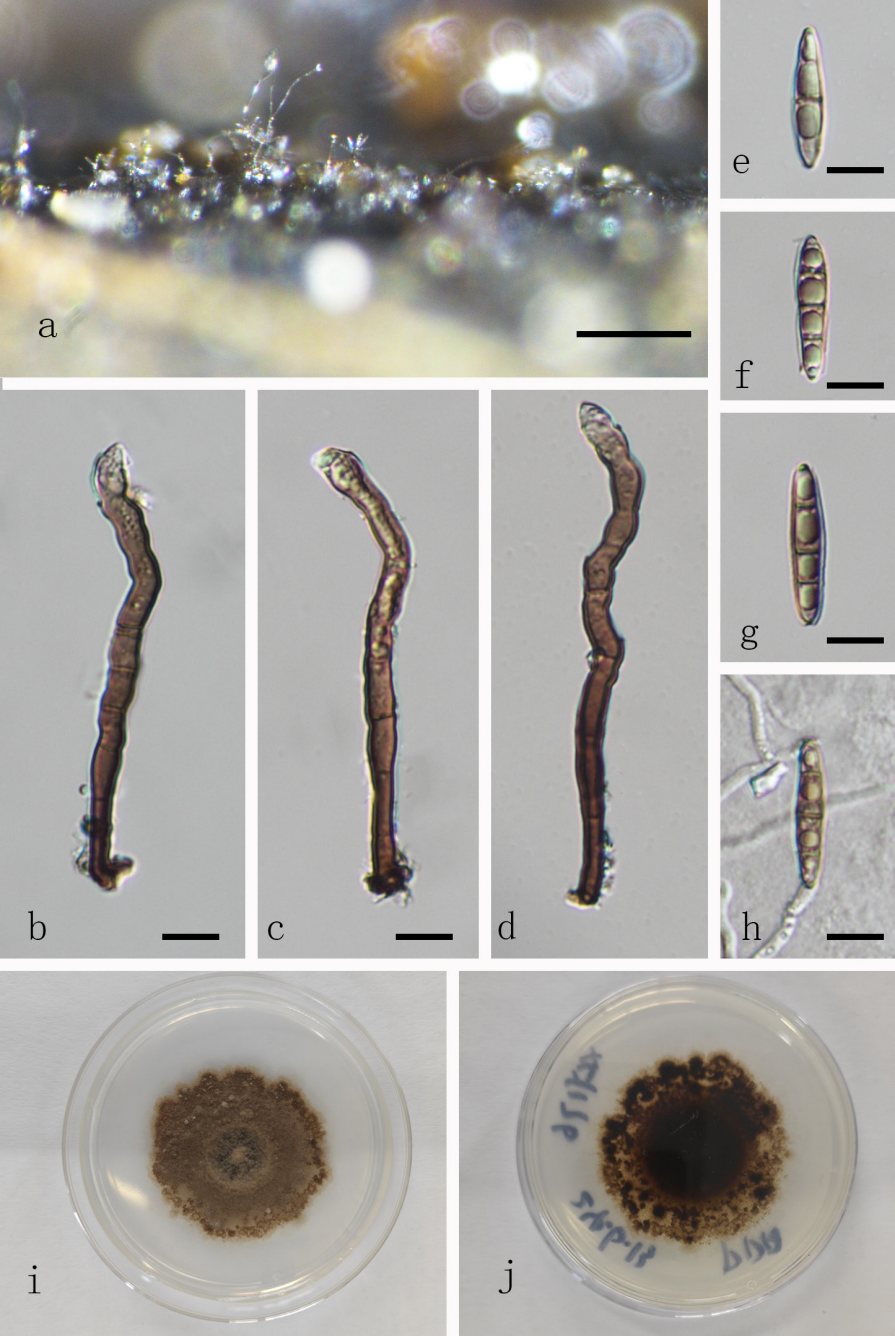
*Wongia lignicola* (HFJAU10529, holotype). **a** Colony on decaying wood. **b–d** Conidiophores. **e–g** Conidia. **h** Germinating conidium. **i–j** Culture on PDA. Scale bars: 100 μm (**a**); 50 μm (**b–h**).

Multi-locus phylogenetic analysis revealed that *Wongia lignicola* (JAUCC 7164, ex-type, and JAUCC 7445) and *W. pallidopolaris* (CBS 440.70, ex-type) formed a sister clade (100% ML, 1.00 BYPP; Fig. 12) within *Wongia*. The sequence similarity between the two strains (JAUCC 7164 and JAUCC 7445) is above 99% for the analyzed genes, confirming their conspecificity. Based on nucleotide comparisons, the strain (JAUCC 7164, ex-type) diverges from *W. pallidopolaris* (CBS 440.70, ex-type) by 27/763 bp (3.5%, without gaps) for *TEF*1-α, while ITS and LSU are nearly identical. Morphologically, *W. lignicola* (HFJAU10529, holotype) is similar to *W. pallidopolaris* (CBS H-25781, holotype) in having ellipsoid to fusiform, 3-septate conidia but differs by having conspicuous guttules in conidia, while the conidia of *W. pallidopolaris* lack guttules, and the outer wall partly detaches from the conidium, with the detached segments appearing as apical or side pockets or wings (Réblová et al. 2025a; Fig. 13). Based on morphological and multi-locus phylogenetic analysis, *W. lignicola* is identified as a new species within *Wongia*.

#### Wongia saprophytica

Taxon classificationAnimaliaDistoseptisporales

S.P. Zheng, D.M. Hu & H.Y. Song sp. nov.

80C806C7-EEA8-575A-9B12-ED838F7AE527

903577

Fig. 14

##### Etymology.

The epithet “saprophytica” refers to the saprobic life mode of the fungus.

##### Diagnosis.

Differs from *W. flava* and *W. miscanthi* by the fusoid to ellipsoidal, or obovoid conidia, subhyaline, 1-septate.

##### Holotype.

HFJAU10499.

##### Description.

***Saprobic*** on decaying submerged reed in freshwater habitats. **Teleomorph**: Undetermined. **Anamorph**: Hyphomycetous. ***Colonies*** on superficial substratum, effuse, hairy, gathered, with hyaline conidia located at the apex of the dark brown to black conidiophores. ***Mycelium*** partly superficial and partly immersed in the substrate, composed of branched, septate, smooth and brown to pale brown hyphae. ***Conidiophores*** 120.5–193 × 3.5–4.5 μm, (x̄ = 146 × 3.5 μm, *n* = 10), macronematous, mononematous, solitary, erect, cylindrical, straight or slightly flexuous, dark brown, gradually paler and becoming pale brown to subhyaline towards the apex, unbranched, septate, thick-walled, smooth to slightly verrucose. ***Conidiogenous cells*** polyblastic, denticulate, terminal, sympodial and subhyaline to pale brown. ***Conidia*** 6.5–12 × 2.5–4.5 μm, (x̄ = 8.5 × 3.3 μm, *n* = 25), acropleurogenous, solitary, fusoid to ellipsoidal, or obovoid, 1-septate, slightly constricted at septa, subhyaline, guttulate, thin and smooth-walled.

##### Culture characteristics.

Conidia germinating on PDA within 24 h. Colonies grew on PDA, reaching 32 mm in 7 days at 25 °C, rough surface, with dense mycelia, dry, umbonate in the middle from the side view, edge undulate, brown to dark brown from above, dark from below.

##### Material examined.

CHINA • Jiangxi Province: Ji’an City, Xiajiang County, 219 Provincial Road, 27°58'49"N, 115°34'31"E, 75.4 m, on submerged unidentified reed in freshwater, 5 Aug 2024, S.P. Zheng, zsp 51 (HFJAU 10499, holotype); ex-type living culture JAUCC 7070, other living culture JAUCC 7177.

##### Notes.

**Figure 14.**
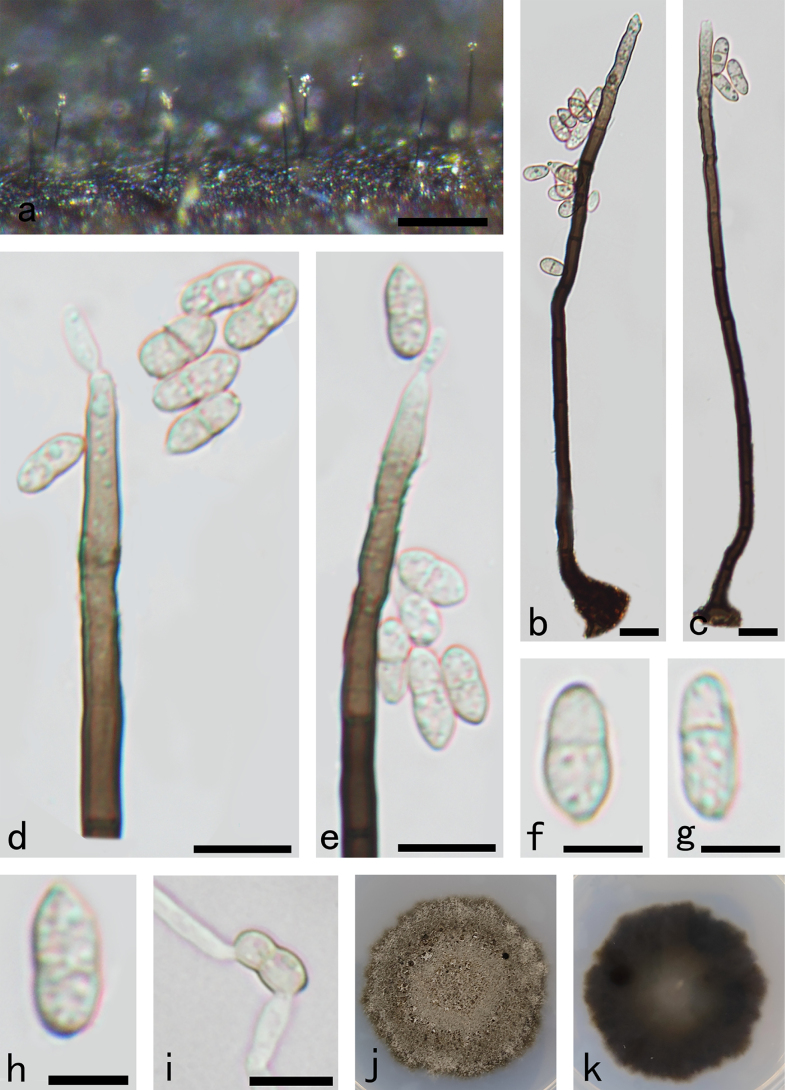
*Wongia saprophytica* (HFJAU10499, holotype). **a** Colony on decaying wood. **b–e** Conidiophores with adhering conidia. **f–h** Conidia. **i** Germinating conidium. **j–k** Culture on PDA. Scale bars: 100 μm (**a**); 10 μm (**b–e, i**); 5 μm (**f–h**).

Phylogenetically, *Wongia saprophytica* (JAUCC 7070, ex-type, and JAUCC 7177) formed a sister branch under *W. flava* (CGMCC 3.25434, ex-type) and *W. miscanthi* (BCRC-FU32062, ex-type) with 100% ML and 1.00 BYPP statistical support (Fig. 12). The sequence similarity between the two strains (JAUCC 7070 and JAUCC 7177) is above 99% for the analyzed genes, confirming their conspecificity. Morphologically, *W. saprophytica* (HFJAU 10499, holotype) can be distinguished from *W. flava* (HKAS 131375, holotype) and *W. miscanthi* (TNM F0037935, holotype) by the fusoid to ellipsoidal or obovoid, subhyaline, 1-septate conidia (Fig. 14), while *W. flava* has fusiform or clavate, pale brown to brown, 1–3 inconspicuously septate conidia (Wang et al. 2024), and *W. miscanthi* has fusiform to cylindric-fusiform, subhyaline conidia, acute or slightly rounded at the apex, obconic at the base with a darkened hilum, and (0–)1–2-septate (Kuo et al. 2024). Therefore, *W. saprophytica* is introduced here as a new species.

### *Hypocreales* Lindau, in Engler & Prantl, Nat. Pflanzenfam., Teil. I (Leipzig) 1(1): 343 (1897)

#### Nectriaceae

Taxon classificationAnimaliaHypocrealesNectriaceae

Tul. & C. Tul. [as ‘Nectriei’], Select. fung. carpol. (Paris) 3: 3 (1865)

0E9DD616-39B8-5583-879C-10E993BB7C7C

##### Notes.

*Nectriaceae* was introduced by Tulasne and Tulasne (1865) and is typified by *Nectria*. Members of *Nectriaceae* exhibit diverse ecological roles. Most are plant-associated, living as saprobes, endophytes, or pathogens that cause diseases such as galls, cankers, rots, and wilts. Some species are facultatively fungicolous or entomogenous, and a few act as human pathogens. The family has a global distribution, with the highest diversity in warm temperate and tropical regions (Rossman et al. 1999; Rossman 2000; Chaverri et al. 2011; Lombard et al. 2015; Perera et al. 2023; Lu et al. 2025).

#### Paracremonium

Taxon classificationAnimaliaHypocrealesNectriaceae

L. Lombard & Crous, in Lombard, van der Merwe, Groenewald & Crous, Stud. Mycol. 80: 233 (2015)

D7B0895D-D6DE-52DD-ABF6-898CCA69E363

##### Notes.

The *Acremonium*-like genus *Paracremonium* was introduced by Lombard et al. (2015) based on different strains from a group of fungi previously classified as *Acremonium recifei* and typified by *P. inflatum*. *Paracremonium* is known only by the anamorph and characterized by the formation of sterile coils from which conidiophores radiate with inconspicuously swollen septa in the hyphae. A new species of *Paracremonium* was collected from a freshwater habitat in Jiangxi Province, China.

#### Paracremonium jiangxiense

Taxon classificationAnimaliaHypocrealesNectriaceae

S.P. Zheng, D.M. Hu & H.Y. Song sp. nov.

4D8E0609-302A-527F-AB2C-A90B1C96FABF

903578

Fig. 16

##### Etymology.

The epithet “jiangxiense” refers to the type locality, Jiangxi Province, China.

##### Diagnosis.

Differs from *P. ellipsoideum* by longer conidiogenous cells and conidia.

##### Holotype.

HFJAU10448.

##### Description.

***Saprobic*** on decaying submerged wood in freshwater habitats. **Teleomorph**: Undetermined. **Anamorph**: Hyphomycetous. ***Colonies*** on wood substrate effuse, caespitose, white, glistening, slimy. ***Mycelium*** immersed in the substrate, hyaline, smooth and thin-walled, branched, septate hyphae. ***Conidiophores*** arising solitary or in clusters, erect, branched, septate, thick-walled, hyaline. ***Conidiogenous cells*** borne on aerial hyphae solitary, straight, acicular, tapering towards the apex, smooth, hyaline, 26–43 µm long, 2.4–3.5 µm wide at base (x̄ = 38 × 3.1 μm, *n* = 10). ***Conidia*** in slimy head, unicellular, aseptate, ellipsoidal to fusiform, with apiculate bases, smooth, thick-walled, hyaline, 12.5–16.6 × 7.4–9.9 μm (x̄ = 14.7 × 8.8 μm, *n* = 25).

##### Culture characteristics.

Conidia germinating on PDA medium within 24 h. Colonies on PDA medium reaching 36 mm diam after 44 days at 25 °C in the dark, flat, felty, margin entire, white, aerial mycelia sparse. Reverse white to cream-yellow.

##### Material examined.

CHINA • Jiangxi Province: Ruijin City, Dingpo Township, Litian Village, Dongjiazhuang, 25°88'5 6"N, 116°02'70"E, 240 m, on submerged unidentified wood in freshwater, 30 Jan 2024, L Huang, zsp 32 (HFJAU10448, holotype); ex-type living culture JAUCC 7045, other living culture JAUCC 7046.

##### Notes.

**Figure 15.**
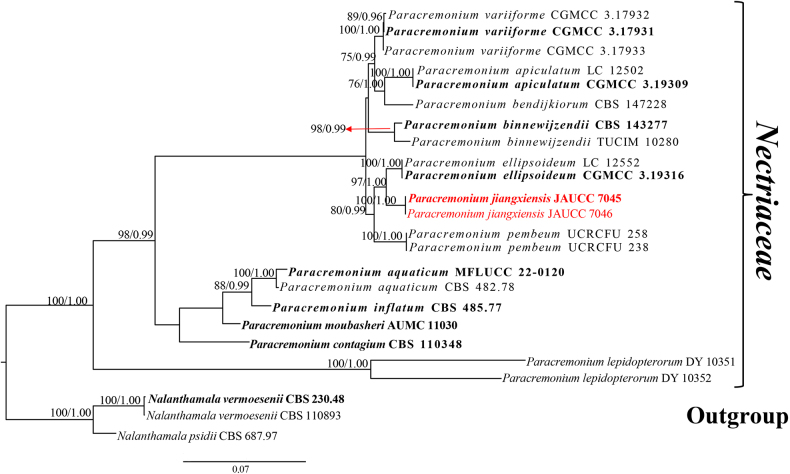
Phylogram generated from maximum likelihood analysis based on combined ITS, LSU, *RPB*2, *TEF*1-α, and *TUB*2 sequence data of 24 taxa, which comprised 3,882 characters. The best-scoring RAxML tree with a final likelihood value of –14110.091277 is presented. The matrix contained 1,135 distinct alignment patterns, with 40.07% of characters undetermined (gaps). Estimated base frequencies were as follows: A = 0.228522, C = 0.280625, G = 0.270734, T = 0.220119; substitution rates AC = 1.493932, AG = 2.825275, AT = 1.482677, CG = 1.233791, CT = 7.470968, GT = 1.0; gamma distribution shape parameter α = 0.341544. Bootstrap support values for ML of at least 70% and BYPP of at least 0.90 are indicated at the nodes as ML/BYPP. Type specimens are in bold, and the new species from the current study are indicated in bold red. The tree is rooted to *Nalanthamala vermoesenii* (CBS 230.48 and CBS 110893) and *N. psidii* (CBS 687.97).

**Figure 16.**
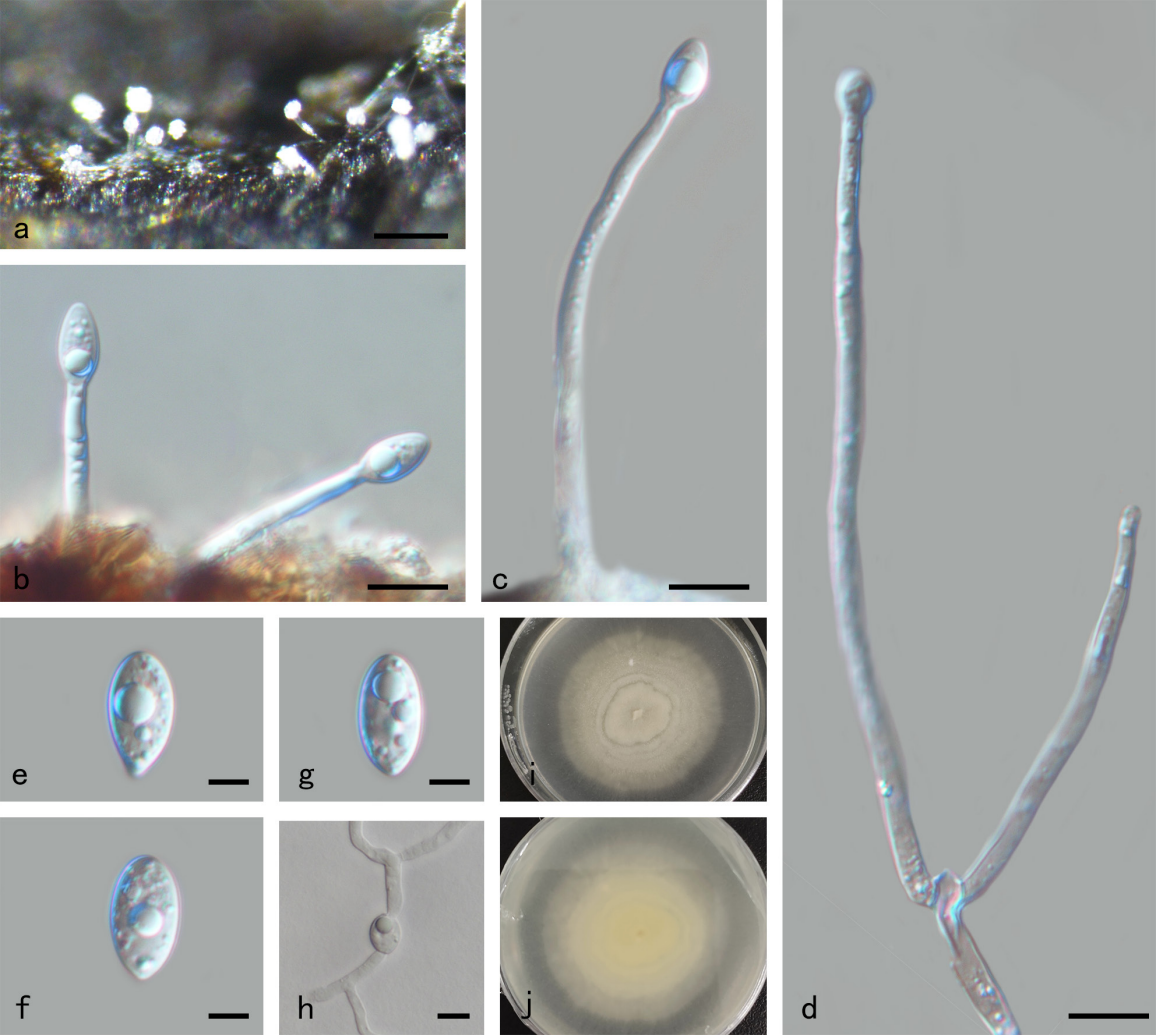
*Paracremonium jiangxiense* (HFJAU10448, holotype). **a** Colony on decaying wood. **b–d** Conidiophores and conidiogenous cells. **e–g** Conidia. **h** Germinating conidium. **i–j** Culture on PDA. Scale bars: 100 µm (**a**); 10 µm (**b–d, h**); 5 µm (**e–f**).

Multi-locus phylogenetic analysis showed that *Paracremonium jiangxiense* (JAUCC 7045, ex-type, and JAUCC 7046) is well separated from *P. ellipsoideum* (CGMCC 3.19316, ex-type, and LC 12552) with 97% ML and 1.00 BYPP statistical support (Fig. 15). The sequence similarity between the two strains (JAUCC 7045 and JAUCC 7046) is above 99% for the analyzed genes, confirming their conspecificity. Based on pairwise nucleotide comparisons, *P. jiangxiense* (JAUCC 7045, ex-type) diverges from *P. ellipsoideum* (CGMCC 3.19316, ex-type) by 7/499 bp (1.4%, without gaps) for ITS, 8/797 bp (1%, without gaps) for LSU, 24/776 bp (3%, without gaps) for *TEF*1-α, and 16/252 bp (6%, without gaps) for *TUB*2. *Paracremonium jiangxiense* differs from the holotype *P. ellipsoideum* in having longer conidiogenous cells (26–43 × 2.4–3.5 μm vs. 14–24 × 1.5–3 μm) and larger conidia (12.5–16.6 × 7.4–9.9 μm vs. 5.5–8 × 3.5–5 μm) (Zhang et al. 2021; Fig. 16). Thus, the strain is identified as a new species, *P. jiangxiense*.

### *Pleurotheciales* Réblová & Seifert, Persoonia 37: 63 (2015)

#### Pleurotheciaceae

Taxon classificationAnimaliaPleurothecialesPleurotheciaceae

Réblová & Seifert, in Réblová, Seifert, Fournier & Štěpánek, Persoonia 37: 63 (2015)

50446412-1AE9-5B4E-92D8-455905128D93

##### Notes.

*Pleurotheciaceae* was established by Réblová et al. (2016) for 10 genera, with *Pleurothecium* as the type genus; it is the only family in *Pleurotheciales*. Currently, 17 genera have been accepted in this family (Wang et al. 2024). Species of this family can be found on a wide range of hosts and substrates, both in terrestrial and freshwater habitats (Hyde et al. 2020b; Bao et al. 2022; Li et al. 2025).

#### Pleurothecium

Taxon classificationAnimaliaPleurotheciales

Höhn., Centbl. Bakt. ParasitKde, Abt. II 60: 26 (1923) [1924]

3A3FE5CE-6EB0-5DE3-81B6-235F3E581A31

##### Notes.

*Pleurothecium* was proposed by von Höhnel (1919), with *P. recurvatum* as the type species. Most species within this genus are anamorphs and are characterized by brown, macronematous conidiophores, polyblastic, sympodially extended denticulate conidiogenous cells, and cylindrical, ellipsoidal, fusiform, or clavate conidia that are hyaline or pigmented (Wang et al. 2025b; Xu et al. 2025). While the teleomorph is similar to that of *Chaetosphaeria*, the anamorph morphology differs (Lin et al. 2025). A new species of *Pleurothecium* is introduced from a freshwater habitat in Jiangxi Province, China.

#### Pleurothecium saprophyticum

Taxon classificationAnimaliaPleurotheciales

C.Y. Xu, D.M. Hu & H.Y. Song sp. nov.

68E04E40-BEE9-50F7-991F-E8654DBAF5D1

903579

Fig. 18

##### Etymology.

The epithet “saprophyticum” refers to the saprobic life mode of the fungus.

##### Diagnosis.

Differs from *P. aquaticum* and *P. guttulatum* by elliptical to obovoid, hyaline, 3-septa conidia.

##### Holotype.

HFJAU10535.

##### Description.

***Saprobic*** on decaying submerged wood in freshwater habitats. **Teleomorph**: Undetermined. **Anamorph**: Hyphomycetous. ***Colonies*** effuse, solitary, black, and hairy, with white conidial masses aggregated at the apex. ***Mycelium*** partly superficial, partly immersed in the substrate, composed of branched, septate, smooth-walled brown hyphae. ***Conidiophores*** 154–280 × 4.5–5.5 μm (x̄ = 217.5 × 5 μm, *n* = 10), macronematous, mononematous, erect, straight or slightly curved, unbranched, septate, smooth, cylindrical, dark brown, and the color becomes lighter towards the apex. ***Conidiogenous cells*** 13.5–34.5 × 3.5–5 μm (x̄ = 24 × 4 μm, *n* = 10), holoblastic, polyblastic, integrated, terminal, smooth-walled, cylindrical, nearly hyaline, sympodial, with cylindrical denticulate projections, and the size of the denticles is 2–3.5 × 1–1.5 μm (x̄ = 2.5 × 1.5 μm, *n* = 15). ***Conidia*** 14–21.5 × 4.5–6.5 μm (x̄ = 17.5 × 6 μm, *n* = 20), solitary, straight or slightly curved, elliptical to obovoid, with a rounded apex, obtuse to attenuate at the base, with 3-septa, hyaline, pointed at the base, rounded at the top, with guttulate.

##### Culture characteristics.

Conidia germinating on PDA within 24 h. Colonies grew on PDA, reaching 33 mm in 60 days at room temperature, irregular, dry, slightly raised in the center, with a radial margin, grayish-white mycelium in the middle, and dark brown to black surroundings; reverse black.

##### Material examined.

CHINA • Jiangxi Province: Yichun City, Jing’an County, Tanxia, 29°04'18"N, 115°36'58"E, 106 m, on submerged unidentified wood in freshwater, 21 Jun 2024, C.Y. Xu, xcy 182 (HFJAU10535, holotype); ex-type living culture JAUCC 7170, other living culture JAUCC 7175.

##### Notes.

**Figure 17.**
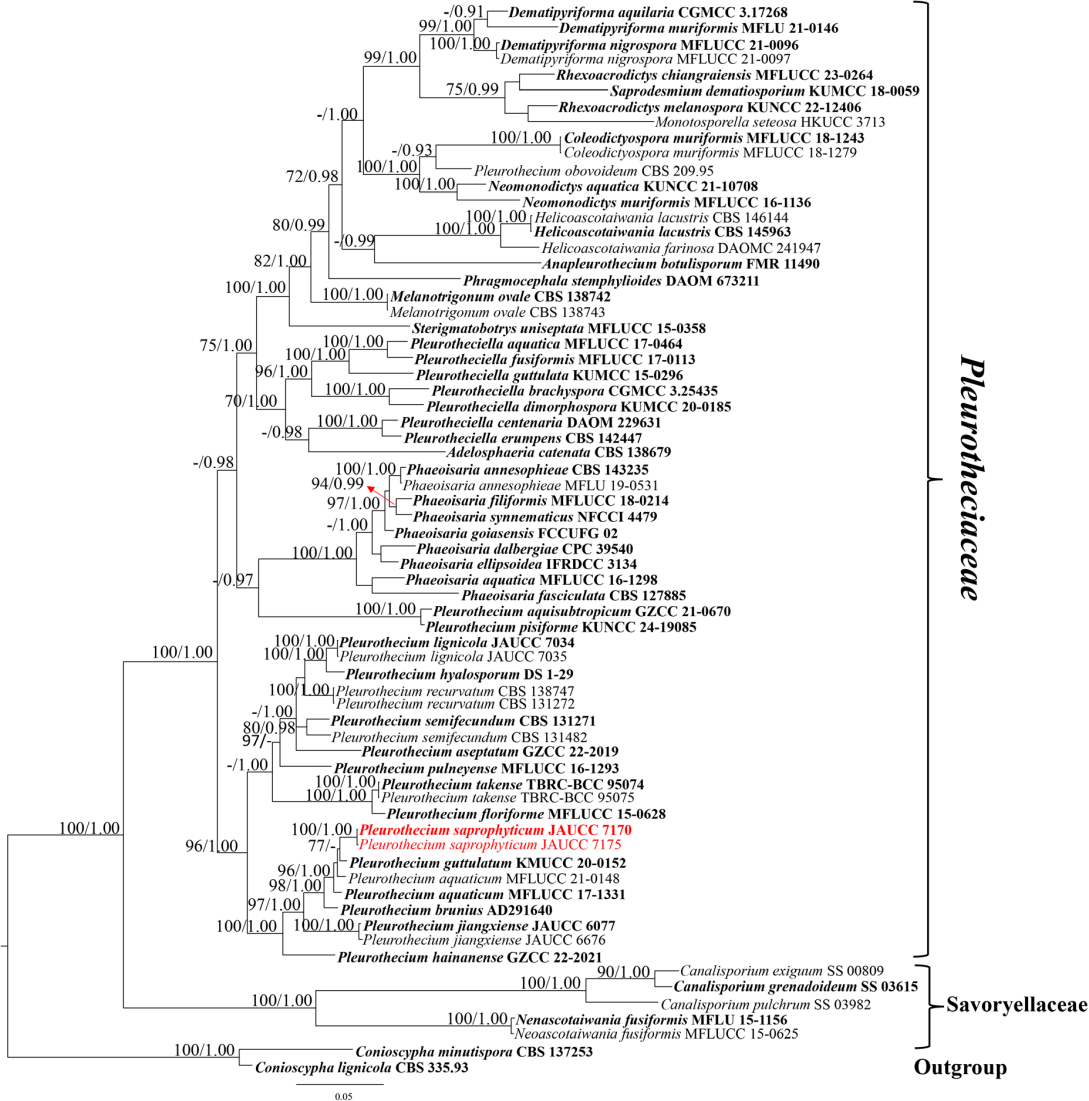
Phylogram generated from maximum likelihood analysis based on combined ITS, LSU, SSU, and *RPB*2 sequence data of 68 taxa, which comprised 4,215 characters. The best-scoring RAxML tree with a final likelihood value of –30599.740843 is presented. The matrix contained 1,586 distinct alignment patterns, with 42.37% of characters undetermined (gaps). Estimated base frequencies were as follows: A = 0.235860, C = 0.260360, G = 0.291882, T = 0.211897; substitution rates AC = 1.527901, AG = 3.070198, AT = 1.766163, CG = 1.120242, CT = 7.433541, GT = 1.0; gamma distribution shape parameter α = 0.192833. Bootstrap support values for ML of at least 70% and BYPP of at least 0.90 are indicated at the nodes as ML/BYPP. Type specimens are in bold, and the new species from the current study are indicated in bold red. The tree is rooted in *Conioscypha lignicola* (CBS 335.93) and *C. minutispora* (CBS 137253).

**Figure 18.**
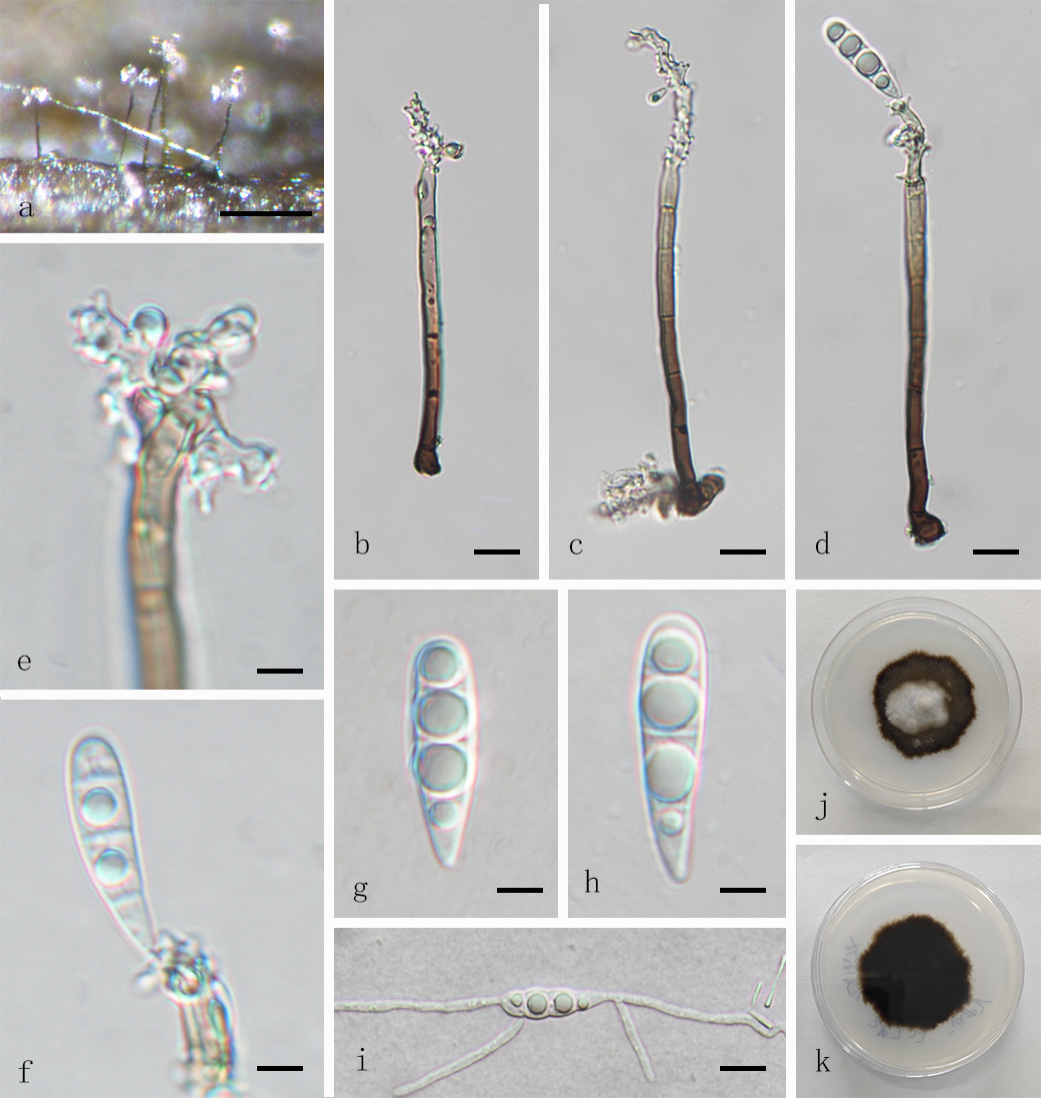
*Pleurothecium saprophyticum* (HFJAU10535, holotype). **a** Colony on decaying wood. **b–d** Conidiophores. **e** Conidiogenous cells. **f** Conidiogenous cells with attached conidium. **g, h** Conidia. **i** Germinated conidium. **j, k** Colony on PDA from above and below. Scale bars: 100 μm (**a**); 10 μm (**b–d**); 4 μm (**e–i**).

In the multi-locus phylogenetic tree, *Pleurothecium saprophyticum* (JAUCC 7170, ex-type, and JAUCC 7175) forms a sister clade with *P. aquaticum* (MFUCC 21-0148) and *P. guttulatum* (KMUCC 20-0152) (77% ML; Fig. 17). The sequence similarity between the two strains (JAUCC 7170 and JAUCC 7175) is above 99% for the analyzed genes, confirming their conspecificity. Morphologically, *P. saprophyticum* (HFJAU10535, holotype) has larger conidiophores than *P. aquaticum* (154–280 × 4.5–5.5 μm vs. 84–110 × 3–4 μm). In addition, *Pleurothecium aquaticum* produces clavate, hyaline, 3-septate, whitish to grayish conidia (18–22 × 4–5 μm) (Luo et al. 2018), and *P. guttulatum* produces clavate, 3–4-septate, hyaline conidia (22–28 × 5–6 μm) (Shi et al. 2021); however, *P. saprophyticum* has elliptical to obovoid, hyaline, 3-septate conidia (14–21.5 × 4.5–6.5 μm) (Fig. 18). Based on pairwise nucleotide comparisons, *P. saprophyticum* (JAUCC 7170, ex-type) diverges from *P. aquaticum* (MFLUCC 17-1331, ex-type) by 18/532 bp (3.3%, without gaps) for ITS and 5/813 bp (0.5%, without gaps) for LSU; it also diverges from *P. guttulatum* (KUMCC 20-0152, ex-type) by 13/524 bp (2.4%, without gaps) for ITS and 5/811 bp (0.6%, without gaps) for LSU. Therefore, *Pleurothecium saprophyticum* is considered a new species within the genus *Pleurothecium*.

### *Rhamphoriales* K.D. Hyde & Hongsanan, Fungal Diversity 107: 94 (2021)

#### Rhamphoriaceae

Taxon classificationAnimaliaRhamphorialesRhamphoriaceae

Réblová, in Réblová & Štěpánek, Mycologia 110(4): 754 (2018)

BD17A859-A835-5102-8915-0DA6DF6747E4

##### Notes.

Réblová and Štěpánek (2018) established the family *Rhamphoriaceae*, which includes *Rhamphoria*, *Rhodoveronaea*, and two newly described genera, *Rhamphoriopsis* and *Xylolentia* (Hyde et al. 2024). It is characterized by glabrous, black, often laterally collapsing ascomata with papillate, rostrate, or long cylindrical necks, cylindrical-clavate, long-stipitate asci arising from croziers, and dictyoseptate or transversely septate ascospores with the ability to produce ascoconidia (Réblová and Štěpánek 2018; Hyde et al. 2020a).

#### Rhamphoriopsis

Taxon classificationAnimaliaRhamphorialesRhamphoriaceae

Réblová & Gardiennet, Mycologia 11 (4): 755 (2018)

A1BFB82C-194D-5B92-A73E-E780823EA17A

##### Notes.

*Rhamphoriopsis* was introduced by Réblová and Štěpánek (2018) and typified by *R. muriformis*. The teleomorph of *Rhamphoriopsis* is characterized by globose ascomata with a cylindrical neck, usually fattened, and hyaline, dictyoseptate ascospores and phaeoisaria-like anamorphs (Réblová and Štěpánek 2018; Lin et al. 2025). In this study, a new species of *Rhamphoriopsis* collected from freshwater habitats is introduced.

#### Rhamphoriopsis jiangxiensis

Taxon classificationAnimaliaRhamphorialesRhamphoriaceae

S.P. Zheng, D.M. Hu & H.Y. Song sp. nov.

2B5A8703-CD40-50F2-925C-F39FD83803D6

903580

Fig. 20

##### Etymology.

The epithet “jiangxiensis” refers to the type locality, Jiangxi Province, China.

##### Diagnosis.

Differs from other *Rhamphoriopsis* species by having erect, unbranched conidiophores and not forming synnemata.

##### Holotype.

HFJAU10660.

##### Description.

***Saprobic*** on decaying submerged wood in freshwater habitats. **Teleomorph**: Undetermined. **Anamorph**: Hyphomycetous. ***Colonies*** effuse, often in small clusters, hairy, with a pale brown powdery mass of conidia. ***Mycelium*** partly superficial, partly immersed in the substrate. ***Conidiophores*** 60–118.5 × 1.5–2.5 μm (x̄ = 91.5 × 2 μm, *n* = 10), macronematous, mononematous, simple, erect, unbranched, straight or slightly flexuous, solitary, thick-walled, septate, cylindrical, with acute apex, dark brown at the base, paler towards the apex. ***Conidiogenous cells*** 8–15 μm long, 1.5–2 μm wide, polyblastic, integrated, terminal becoming intercalary, cylindrical, tapering apically, sympodial, with denticles. ***Conidia*** 2.5–3.5 × 1.5–2 μm (x̄ = 2.8 × 1.8 μm, *n* = 15), solitary, acropleurogenous, dry, aseptate, hyaline to light brown, ellipsoidal to obovoid, smooth and thin-walled.

##### Culture characteristics.

Conidia germinating on PDA within 24 h. Colonies grew on PDA, reaching 30 mm in 60 days at room temperature, subcircular, raised in the center, with an uneven surface featuring furrows, dark brown, brown at the edge; reverse black with relatively smooth but undulate margin.

##### Material examined.

CHINA • Jiangxi Province: Yichun City, Jing’an County, Tanxia, 29°04'18"N, 115°36'58"E, 106 m, on submerged unidentified wood in freshwater, 21 Jun 2024, S.P. Zheng, xcy 183 (HFJAU10660, holotype); ex-type living culture JAUCC 7442, other living culture JAUCC 7167.

##### Notes.

**Figure 19.**
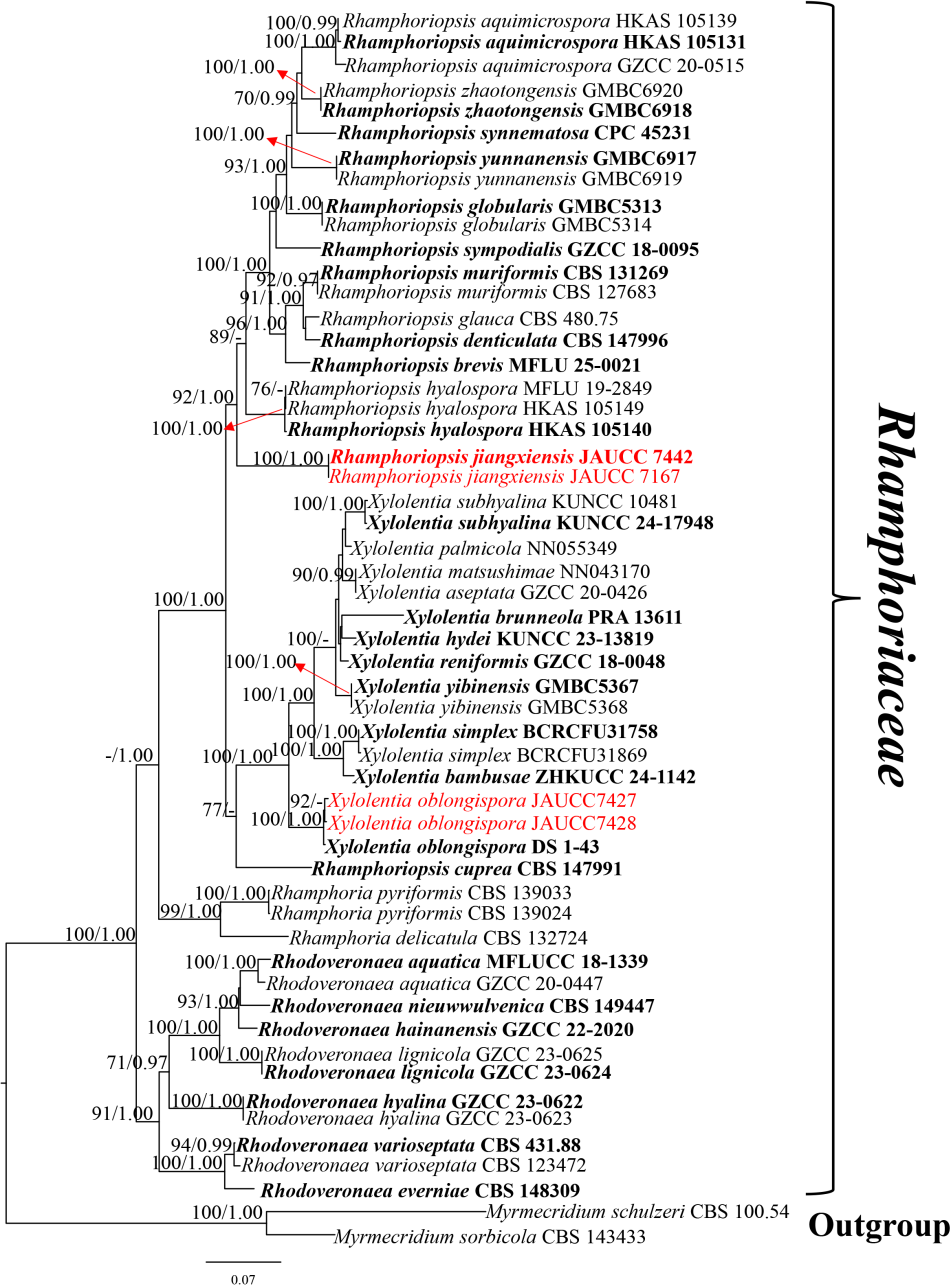
Phylogram generated from maximum likelihood analysis based on combined ITS, LSU, SSU, *RPB*2, and *TEF*1-α sequence data of 54 taxa, which comprised 3,899 characters. The best-scoring RAxML tree with a final likelihood value of –21631.841633 is presented. The matrix contained 1,142 distinct alignment patterns, with 27.67% of characters undetermined (gaps). Estimated base frequencies were as follows: A = 0.240652, C = 0.267944, G = 0.281453, T = 0.209952; substitution rates AC = 1.156181, AG = 3.067937, AT = 3.067937, CG = 3.067937, CT = 7.361090, GT = 1.000000; gamma distribution shape parameter α = 0.162454. Bootstrap support values for ML of at least 70% and BYPP of at least 0.90 are indicated at the nodes as ML/BYPP. Type specimens are in bold, and the new species from the current study are indicated in bold red. The tree is rooted to *Myrmecridium sorbicola* (CBS 143433) and *M. schulzeri* (CBS 100.54).

**Figure 20.**
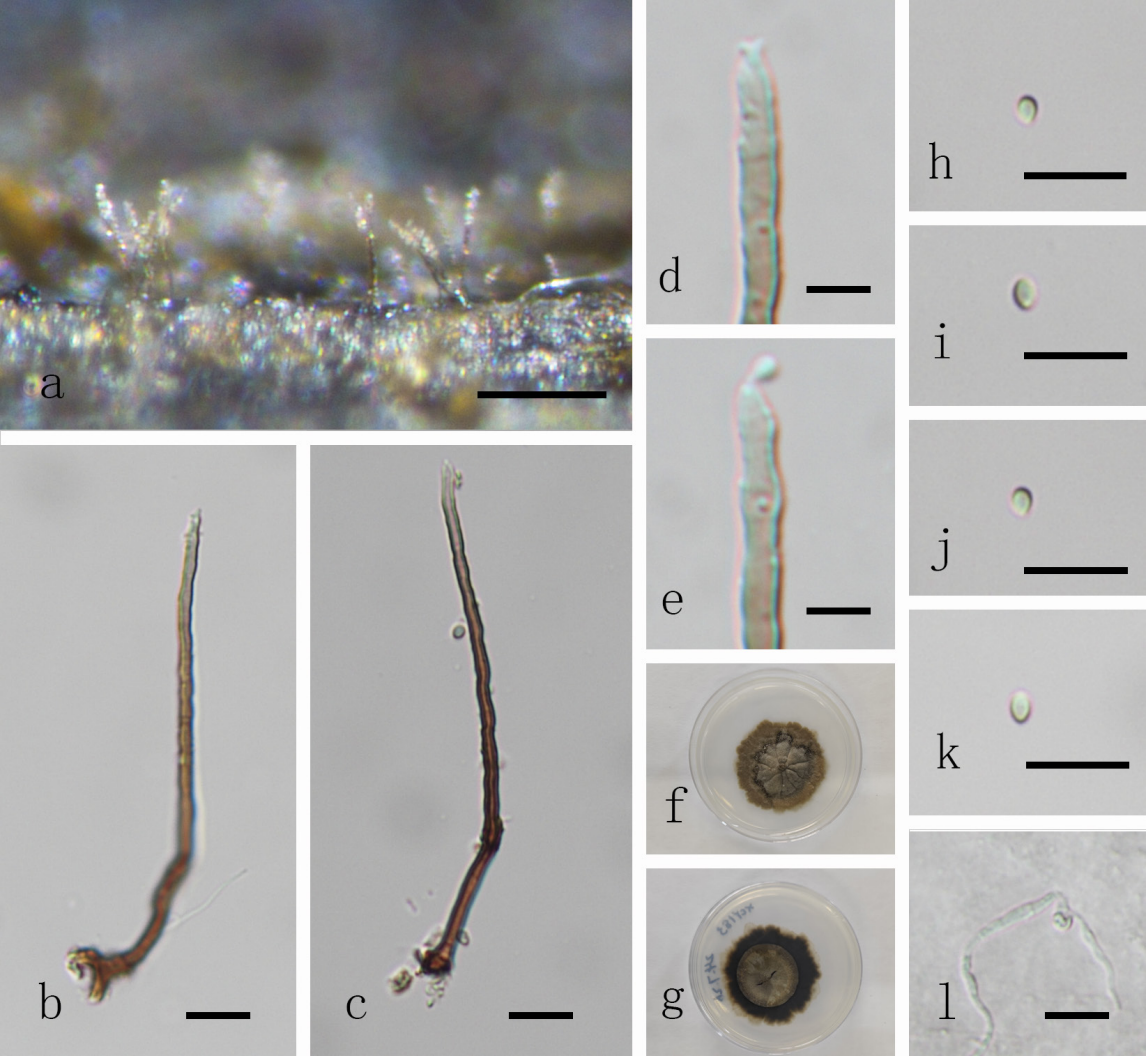
*Rhamphoriopsis jiangxiensis* (HFJAU10660, holotype). **a** Colonies on the natural substratum. **b, c** Conidiophores. **d** Conidiogenous cell. **e** Conidiogenous cell bearing conidium. **h–k** Conidia. **l** Germinated conidium. **f, g** Colony on PDA from above and below. Scale bars: 100 μm (**a**); 10 μm (**b, c, h–l**); 4 μm (**d, e**).

Phylogeny based on combined ITS, LSU, SSU, *TEF*1-α, and *RPB*2 sequence data indicates that *Rhamphoriopsis jiangxiensis* (JAUCC 7167, JAUCC 7442, ex-type) forms an independent branch with 92% ML and 1.00 BYPP statistical support within *Rhamphoriopsis* (Fig. 19). The sequence similarity between the two strains (JAUCC 7167 and JAUCC 7442) is above 99% for the analyzed genes, confirming their conspecificity. BLASTn searches of ITS showed 87% similarity to *R. hyalospora* (MN846344), and LSU, SSU, and *RPB*2 showed 98%, 96%, and 85% similarity to *Rhamphoriopsis* sp. (PZ052314, PV939456, PX373388, respectively). The morphological characteristics of *R. jiangxiensis* align with the genus characteristics, such as cylindrical conidiogenous cells tapering at the apex and polyblastic, aseptate conidia that are elliptical to obovoid (Réblová and Štěpánek 2018). However, *R. jiangxiensis* can be distinguished from other species by having erect, unbranched conidiophores and not forming synnemata (Fig. 20, Table 3). Based on morphological characteristics and phylogenetic analysis, *R. jiangxiensis* is established as a new species within *Rhamphoriopsis*.

**Table 3.** Comparison of asexual morphological characteristics of *Rhamphoriopsis* species.

Species	Conidiophores	Conidiogenous cells	Conidia	Reference
*R. aquimicrospora*	Synnematous, septate, cylindrical, reddish brown, with the terminal of conidiophores splaying out as a flared head, 100–180 × 1.2–2.4 µm	Integrated, terminal, polyblastic, hyaline to pale brown, sympodially extending with numerous denticles	Ellipsoidal, aseptate, smooth-walled, hyaline, 2–3.2 × 1.3–2 µm	Yang et al. 2023
*R. brevis*	Scattered or in small groups, unbranched, cylindrical, subulate, pale brown at the base, paler towards the apex, mostly reduced to conidiogenous cells, 19–52 × 1.7–3 μm	Integrated, polyblastic, terminal becoming intercalary, cylindrical, tapering apically, sympodial, with numerous denticles	Ellipsoidal to obovoid, solitary, acropleurogenous, aseptate, hyaline, 2.7–3.6 × 1.2–2.3 μm	Lin et al. 2025
*R. cuprea*	Solitary, cylindrical, unbranched, pale brown to red brown, subhyaline towards the apex, 30–90 × 2–3 µm	Integrated, terminal, polyblastic, sympodially extending, cylindrical, pale brown near the base, hyaline to subhyaline towards the apex, with numerous denticles, 13–22 × 2–2.5 µm,	Obovoid to dacryoid, hyaline, aseptate, secession schizolytic 4–6.5 × 1.5–2 µm	Réblová et al. 2025b
*R. glauca*	/	Arising from undifferentiated or slightly thick-walled hyphae, hyaline to pale brown, variable in shape and size, cylindrical, forming conidia by sympodial growth on conspicuous denticles	Guttuliform to ellipsoidal, hyaline, occasionally becoming inflated and giving rise to some globose to ellipsoidal secondary conidia or a short secondary conidiophore, 2.5–3.5 × 1.6–2.2 µm	Yang et al. 2023
*R. globularis*	Synnematous, subcylindrical to cylindrical, brown, aseptate to septate, with terminal cells giving rise to clusters of conidiogenous cells, 200–450 × 21–41 μm	Integrated, terminal, polyblastic, hyaline to pale brown, sympodially extending with numerous denticles	Globose to ovoid, solitary, acropleurogenous, aseptate, hyaline, 2.5–4.2 × 2–3.7 μm	Liu et al. 2025b
*R. hyalospora*	Caespitose, closely packed together but not forming synnemata, unbranched or branched, aseptate to septate, cylindrical, subulate, pale brown to dark brown, rarely reduced to conidiogenous cells, 40–145 × 2–3.5 μm	Integrated, polyblastic, terminal becoming intercalary, cylindrical, tapering apically, sympodial, with numerous indistinctive denticles, hyaline to pale brown, 23.0–115.0 × 1.5–3.0 μm	Ellipsoidal to obovoid, solitary, acropleurogenous, aseptate, hyaline, 2.0–3.7 × 1.8–2.6 μm	Lin et al. 2023
***R. jiangxiensis***	Cylindrical, solitary, unbranched, septate, with acute apex, dark brown at the base, paler towards the apex, 60–118.5 × 1.5–2.5 μm	Integrated, polyblastic, terminal becoming intercalary, cylindrical, tapering apically, sympodial, with denticles, 8–15 × 1.5–2 μm	Ellipsoidal to obovoid, solitary, acropleurogenous, aseptate, hyaline to light brown, 2.5–3.5 × 1.5–2 μm	This study
*R. muriformis*	Cylindrical, pale brown to red-brown, subhyaline towards the apex, unbranched or rarely branched, 40−66 × 3–3.5 µm	Integrated, terminal, cylindrical, hyaline to pale brown, polyblastic, sympodially extending with numerous denticles, 12–22 µm long, 2.5–3 µm	Ellipsoidal to obovoid, straight, hyaline, aseptate, (3.5−)4.5−5(–5.5) × 1.5−2 µm	Réblová and Štěpánek 2018
*R. sympodialis*	Scattered or in small groups, unbranched, cylindrical, subulate, 0–1-septate, hyaline to pale brown, mostly reduced to conidiogenous cells, 14–45 × 1.9–3.3 μm	Integrated, polyblastic, terminal becoming intercalary, cylindrical, sympodial, with numerous indistinctive denticles, 11–42 × 1.8–3.2 μm	ellipsoidal to obovoid, solitary, acropleurogenous, aseptate, hyaline, 1.9–4 × 1.4–2.2 μm	Hyde et al. 2020a
*R. synnematosa*	Conidiophores dimorphic, micronematous conidiophores in loose tufts on hyphae, subcylindrical, base pale brown, smooth, multiseptate, 15–60 μm tall; macronematous conidiophores synnematal, consisting of tufts of red-brown, smooth, cylindrical	Terminal cells giving rise to clusters of conidiogenous cells, polyblastic loci, subcylindrical, pale brown, 15–35 × 2–2.5 μm, with several terminal denticles, with terminal and lateral sympodial	Ellipsoid, solitary, hyaline, aseptate, 2.5–3 × 1.5–2 μm	Crous et al. 2023
*R. uniseptata*	Solitary or rarely aggregated in small groups, unbranched, brown, becoming slightly paler towards the apex, septate, 77.6–174 × 2.8–4.3 µm	Apical conidiogenous cells hyaline, integrated, polyblastic, restricted to terminal position, cylindrical to lageniform, swollen at the conidiogenous loci, proliferation sympodial, denticles absent, 3.4–5 × 2.3–3.8 µm; Secondary intercalary conidiogenous cells hyaline to pale brown, occasionally observed, without conidia, with apical and middle swollen, 4–5.6 × 2.6–4.7 µm	Oblong, obovoid or cylindrical, hyaline to light brown, solitary, acropleurogenous, asymmetrically shaped, with a distinct basal hilum, 1-septate, with guttules, 8.6–11.5 × 3.2–4.3 µm	Pitakpattanakul et al. 2025
*R. yunnanensis*	Synnematous, septate, cylindrical, reddish brown, with terminal of conidiophores splaying out as a fared head	Integrated, polyblastic, terminal, hyaline to pale brown, sympodially extending with numerous denticles	Ellipsoidal, aseptate, hyaline, 2–3 × 1.5–2.2 µm	Liu et al. 2025b
*R. zhaotongensis*	Synnematous, septate, cylindrical, reddish brown, with terminal of conidiophores splaying out as a fared head	Integrated, polyblastic, terminal, hyaline to pale brown, sympodially extending with numerous denticles	Ellipsoidal, aseptate, hyaline, 1.9–2.8 × 1.4–1.9 µm	Liu et al. 2025b

#### Xylolentia

Taxon classificationAnimaliaRhamphorialesRhamphoriaceae

Réblová & Gardiennet, in Réblová & Štěpánek, Mycologia 110(4): 754 (2018)

69D77748-945A-5B54-AAA9-78825283D7AD

##### Notes.

*Xylolentia* was introduced by Réblová and Štěpánek (2018), with *X. brunneola* as the type species, which is distinct from other genera in *Rhamphoriaceae* by uniseptate brown ascospores. The teleomorph of *Xylolentia* is characterized by globose, hairless, black ascomata featuring a cylindrical neck, unitunicate cylindric-clavate asci with long pedicels and a distinct inamyloid apical ring, and ellipsoidal to obovoid brown septate ascospores (Réblová and Štěpánek 2018). The anamorph is characterized by unbranched, septate conidiophores; polyblastic conidiogenous cells with sympodially extending rachides; and hyaline and brown, ellipsoidal to obovoid conidia aggregated in slimy masses (Réblová and Štěpánek 2018; Lin et al. 2025). The genus currently comprises 11 accepted species, all of which were confirmed by sequence data (Index Fungorum 2026). *Xylolentia* species are saprobes and are mostly distributed in China (Zhao et al. 2025), except for *X. brunneola* and *X. matsushimae*, which have been reported in the Czech Republic and Japan, respectively (Réblová and Štěpánek 2018; Wu and Diao 2022). This study introduces a new collection of *X. oblongispora* from a newly discovered habitat.

#### Xylolentia oblongispora

Taxon classificationAnimaliaRhamphorialesRhamphoriaceae

C.G. Lin, K.D. Hyde & Jian K. Liu, Fungal Diversity 135: 400 (2025)

89DB71B2-865B-55AA-8B4E-FC5436EC4FFF

903879

Fig. 21

##### Description.

Saprobic on decaying submerged wood in freshwater habitats. **Anamorph**: Hyphomycetous. Colonies on wood effuse, hairy, scattered or aggregated, brown, with glistening conidial masses at the apex. Mycelium partly superficial, partly immersed, composed of septate, smooth-walled, brown to hyaline hyphae. Conidiophores macronematous, mononematous, erect, straight or slightly flexuous, solitary or aggregated in small groups, cylindrical, smooth-walled, septate, unbranched, brown to dark brown, pale brown to subhyaline at the apex, 129–211 × 3.4–5 μm (x̄ = 155 × 4 μm, *n* = 10). Conidiogenous cells holoblastic, polyblastic, integrated, terminal, elongating percurrently, determinate, cylindrical to cylindric-lageniform, subhyaline to pale brown, 19–24 × 2.5–3.5 μm (x̄ = 21.4 × 3 μm, *n* = 10). Conidia acrogenous, aggregated in slimy masses, oblong to ellipsoidal, subglobose, 3.3–4.6 × 2.3–3.3 μm (x̄ = 4 × 2.8 μm, *n* = 30), aseptate, hyaline to light brown, guttulate, smooth, and thin-walled. **Teleomorph**: Undetermined.

##### Culture characteristics.

Conidia germinating on PDA within 24 h. Colonies grew on PDA, reaching 27 mm in 70 days at room temperature, circular, raised, with compact mycelium on the surface, dark brown to black, yellowish brown at the edge; reverse pale brown with an entire margin.

##### Material examined.

CHINA • Jiangxi Province: Nanchang City, Meiling Ecotourism Resort, Xinjian District, 27°58'49"N, 115°34'31"E, 75.4 m, on submerged unidentified wood in freshwater, 5 Aug 2024, S.P. Zheng, zsp 69 (HFJAU10665); living culture JAUCC 7427 and JAUCC 7428.

##### Known hosts.

Terrestrial decaying wood (Lin et al. 2025), on submerged unidentified wood (this study).

##### Known distribution.

China, Guizhou Province (Lin et al. 2025), Jiangxi Province (this study).

##### Notes.

**Figure 21.**
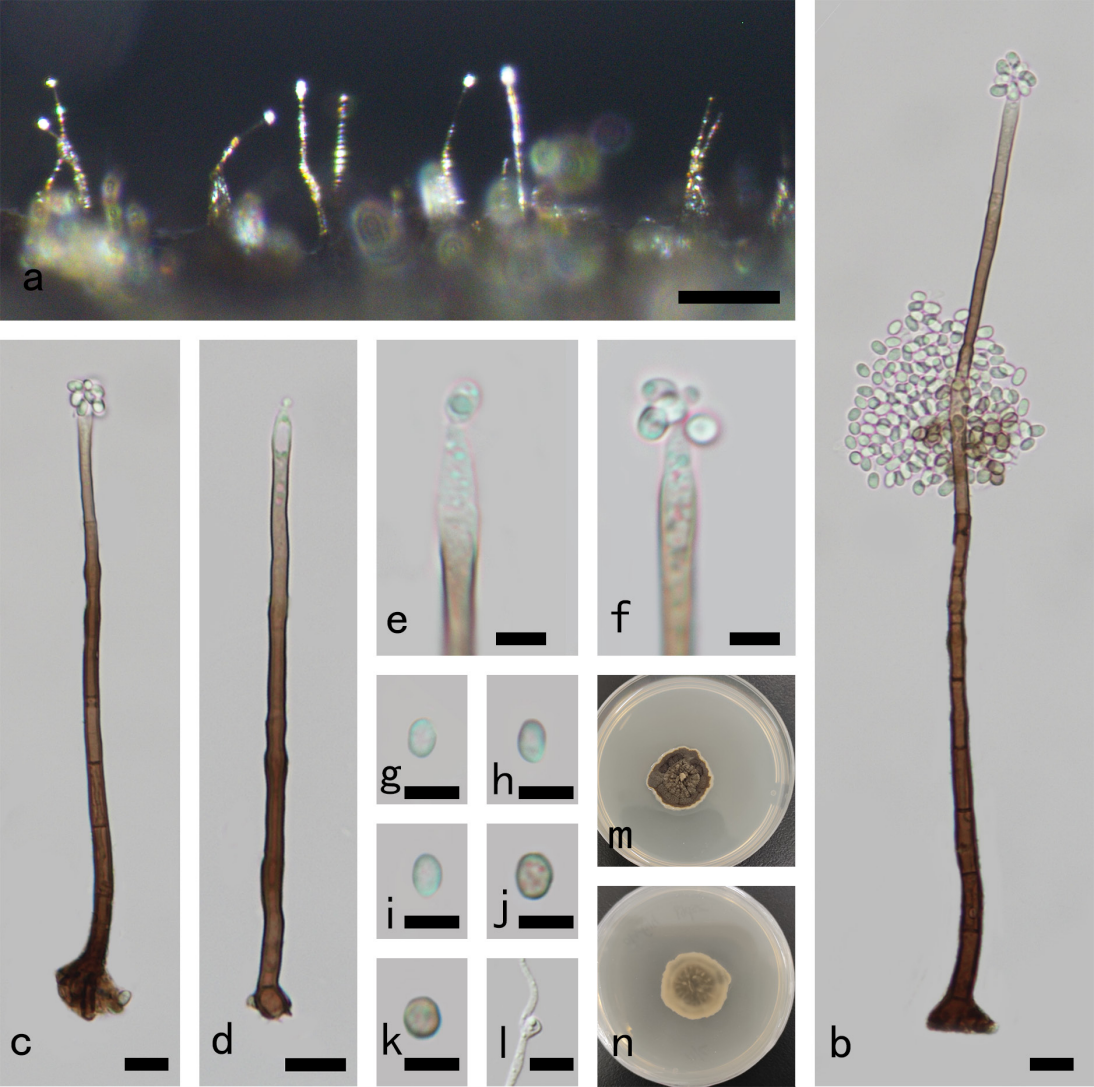
*Xylolentia oblongispora* (HFJAU10666). **a** Colony on the wood. **b–d** Conidiophores and conidia. **e–f** Conidiogenous cells and conidia. **g–k** Conidia. **l** Germinated conidium. **m, n** Colony on PDA. Scale bars: 100 μm (**a**); 50 μm (**b–d**); 10 μm (**b–h**).

*Xylolentia oblongispora* was originally described as saprobic from a terrestrial habitat in Guizhou Province, China (Lin et al. 2025). In the phylogenetic tree, the isolates (JAUCC 7427 and JAUCC 7428) clustered with *X. oblongispora* (GZCC 18-0054, ex-type) with 100% ML and 1.00 BYPP statistical support (Fig. 19). The BLAST results of ITS, LSU, SSU, and *TEF*1-α genes also showed 100% similarity with *X. oblongispora* (PQ898745, PQ898781, PQ898815, and PV040797, respectively). Morphologically, the features of the strain are almost the same as those of the holotype *X. oblongispora* but exhibit newly observed characters: ellipsoidal to subglobose conidia that are hyaline to light brown, while the holotype has oblong conidia with obtuse ends and is hyaline (Lin et al. 2025; Fig. 21). These differences are attributed to morphological plasticity induced by distinct habitat conditions. Therefore, the strain is introduced as a new collection of *X. oblongispora*.

## Discussion

The discovery of 13 novel or newly recorded freshwater species across 10 genera enriches the inventory of freshwater fungal resources in China and further clarifies the morphological diversity and taxonomic characteristics of freshwater fungi in this region. Specifically, this study reports the first records of *Hilberina* and *Neodendryphiella* species from freshwater habitats, filling gaps in the ecological distribution records of these two genera and providing critical baseline data for understanding their evolution between aquatic and terrestrial ecosystems. In contrast, the remaining seven genera have been documented in both terrestrial and freshwater environments, further supporting the ecological versatility of freshwater fungi and highlighting their ability to colonize and adapt to diverse habitats.

### Research status and challenges of *Dendryphiella*

*Dendryphiella* currently comprises 22 epithets in Index Fungorum (2026), yet only nine have associated sequence data (Du et al. 2025). It is important to note that among the numerous species described before 2000 based on morphology, only *D. vinosa* (= *D. interseminata*) has been recollected and its taxonomy confirmed through molecular phylogenetics. This lack of molecular data presents a fundamental challenge, making the recollection and sequencing of these taxa, particularly from type localities, a future task to resolve their phylogenetic placement and validity. Furthermore, ecological boundaries within the group are unclear. While all species were reported from terrestrial habitats, and some marine taxa have been segregated into *Paradendryphiella* (Woudenberg et al. 2013), only a new collection of *D. vinosa* collected from freshwater by Dong et al. (2020) and a new freshwater species are introduced in this study, suggesting a broader ecological range than previously assumed. These findings highlight a knowledge gap and the importance of expanded sampling from diverse freshwater and marginal marine habitats.

### Research status and challenges of *Neodendryphiella*

*Neodendryphiella* was established as a novel genus in 2018, with five species currently recognized, all taxonomically validated through an integrative approach combining morphological characterization and molecular phylogenetic analyses (Iturrieta-González et al. 2018; Crous et al. 2025). To date, all documented *Neodendryphiella* taxa have been isolated from terrestrial habitats, and the species reported in this study thus represents the first record of this genus in aquatic environments.

### Research status and challenges of *Hilberina*

To date, 19 *Hilberina* species are listed in Index Fungorum (2026). Most of these taxa were established through nomenclatural combinations based on morphological characteristics (Miller et al. 2014), while only six species, *H. caudata*, *H. hongheensis*, *H. sphagnorum*, *H. punctata*, *H. robusta*, and *H. munkii*, have been confirmed with molecular data from LSU and *TUB* only (Yang et al. 2023). Phylogenetically, *Hilberina* and *Helminthosphaeria* are polyphyletic, with their type species (*Hel. clavariarum* and *H. caudata*) occurring as sister species on an unsupported branch (Miller et al. 2014; Yang et al. 2023; Lu et al. 2025). Therefore, the genus currently confronts two critical taxonomic challenges that impede a robust understanding of its systematic position and species delimitation. First, the genus exhibits a polyphyletic phylogenetic topology, with its intergeneric taxonomic boundary with *Helminthosphaeria* remaining poorly defined and ambiguous. This phylogenetic non-monophyly and unclear generic demarcation have long hindered its accurate placement within the broader fungal taxonomic framework and obscured its evolutionary affinities with related genera. Second, most *Hilberina* species have been described solely on the basis of traditional morphological characteristics, and most lack molecular sequence data. Even for species with such data, additional loci remain missing (e.g., *TEF*1-α and *RPB*2). Thus, a comprehensive, systematic accumulation of molecular and morphological data from fresh collections is urgently required to re-evaluate the generic circumscription, resolve phylogenetic uncertainties, and clarify the species composition of *Hilberina*. Regarding its ecology, Luo et al. (2004) documented *H. breviseta* during their investigation of freshwater fungal diversity in Lake Dianchi, Yunnan Province. This species was recorded with two occurrences, without detailed morphological descriptions or supporting molecular data. The new species described here represents the first valid record of this genus in aquatic environments.

### Research status and challenges of *Sporidesmiella*

The number of species in *Sporidesmiella* has expanded substantially, with 63 records listed in Index Fungorum (2026). However, only half of these species have been phylogenetically validated with supporting DNA sequence data; this remains problematic (Shenoy et al. 2006). Prior to 2000, more than 20 species had been documented, all lacking corresponding molecular data, and even the eight new species (*S. archidendri*, *S. bawanglingensis*, *S. guangdongensis*, *S. jiangxiensis*, *S. jiulianshanensis*, *S. lushanensis*, *S. machili*, and *S. nanlingensis*) subsequently described by Ma et al. 2012, 2015 and Ma 2016a, 2016b were defined solely based on traditional morphological characteristics. The extensive morphological overlap among *Sporidesmiella* and related genera (*Sporidesmium* and *Endophragmia*), combined with a critical lack of molecular data, lies at the heart of the group’s taxonomic difficulties. Therefore, fresh collections and multi-locus sequencing of existing and putative novel *Sporidesmiella* taxa are required. This work is critical for validating the taxonomic status of morphologically defined species, resolving cryptic species within the genus, and providing the molecular data necessary for robust phylogenetic analyses. Second, morphological overlap across genera raises the question of whether subtle differences (e.g., conidial size and septation number) between *Sporidesmiella*, *Sporidesmium*, and *Endophragmia* represent genuine phylogenetic divergence or intraspecific variation driven by ecological or environmental factors. Resolving these concerns requires an integrative analysis of morphological traits and molecular data from conspecific isolates across different habitats. Finally, any introduction of a new species must be based on an integrative approach that combines detailed morphological characterization, multi-locus molecular sequencing, and comparative phylogenetic analysis with closely related taxa to avoid misdelimitation and ensure taxonomic stability.

### Research status and challenges of *Wongia*

There are 14 accepted species of *Wongia* in Index Fungorum (2026), all confirmed by molecular data (Bao et al. 2025). All species were reported as saprobic and were mainly isolated from freshwater habitats, and only four were collected from terrestrial habitats (Luo et al. 2019; Bao et al. 2025; Réblová et al. 2025a). *Wongia bandungensis* is synonymized under *W. suae* on the basis of minor phylogenetic differences and morphological resemblance (Bao et al. 2025). In the same study, *W. guizhouensis* was introduced as a new species, but it also shows minor sequence divergence relative to the closely related species *W. fusiformis*. In the multi-locus phylogenetic analysis, *W. guizhouensis* and *W. fusiformis* were also observed clustered in the same clade, consistent with the results of Bao et al. (2025). Given the limited molecular divergence observed, the differences in conidial septation may represent intraspecific morphological variation rather than species-level distinction (Bao et al. 2021; Bao et al. 2025).

### Research status and challenges of *Paracremonium*

There are 11 recognized species in *Paracremonium* (Index Fungorum 2026), all of which have been taxonomically validated through molecular analyses. Notably, the phylogenetic placement and interspecific relationships have been well resolved, with no ambiguities. *Paracremonium* has been isolated from various sources, such as soil, freshwater, and sewage, and is associated with human infections (Lombard et al. 2015; Lynch et al. 2016; Crous et al. 2017; Zhang et al. 2017; Al-Bedak et al. 2019; Crous et al. 2021a; Ming et al. 2021; Liu et al. 2024b). The species described here is the fourth freshwater species in this genus (Liu et al. 2024b).

### Research status and challenges of *Pleurothecium*

Currently, 22 records are accepted in *Pleurothecium* (Index Fungorum 2026). Among them, *P. leptospermi* and *P. obovoideum* were transferred to *Anapleurothecium* (*A. leptospermi*) and *Parapleurothecium* (*Pa. obovoideum*), respectively, based on morphological and molecular analyses by Monteiro et al. (2022) and Lin et al. (2025). Within the genus *Pleurothecium*, two species described prior to 2000, *P. magnum* and *P. malayense*, lack molecular data. Notably, four species documented in the molecular taxonomy era (i.e., *P. bicoloratum*, *P. clavatum*, *P. leptospermi*, and *P. yunnanense*) lack corresponding molecular validation. Additionally, phylogenetic analyses reveal that *P. pisiformis* and *P. aquisubtropicum* form a distinct clade, which is sister to *Phaeoisaria* rather than clustering with other *Pleurothecium* species; these phylogenetic findings align with the phylogenetic analyses of Wang et al. (2025b), who noted that *P. pisiformis* exhibits morphological traits consistent with the current circumscription of *Pleurothecium*. Therefore, this conflicting morphological and phylogenetic evidence collectively confirms that *Pleurothecium* is a polyphyletic genus. *Pleurothecium* species have previously been reported as saprobes living in both terrestrial and freshwater environments.

### Research status and challenges of *Rhamphoriopsis*

*Rhamphoriopsis* comprises 12 species, all confirmed by sequence data (Index Fungorum 2026), representing a monophyletic clade within the family *Rhamphoriaceae* in molecular phylogenetic analyses. Members of *Rhamphoriopsis* were reported from decaying wood as saprobes, mainly species isolated from terrestrial habitats, and only three species collected from aquatic habitats (Réblová and Štěpánek 2018; Lin et al. 2025). The species described here is the fourth freshwater species collected from *Rhamphoriopsis*.

### Research status and challenges of *Distoseptispora*

In a decade (2016–2026), 117 *Distoseptispora* species have been deposited in Index Fungorum (2026), all confirmed by both morphological and molecular characters. *Distoseptispora* has experienced an increase in newly described species, with considerable ambiguity in species delimitation. Many studies have confirmed that conspecific *Distoseptispora* isolates collected from different habitats (aquatic or terrestrial) or across different seasons and years may exhibit significant morphological differences, particularly in conidial size (Yang et al. 2018, 2021; Shen et al. 2024, 2025). Even so, numerous newly proposed *Distoseptispora* species are diagnosed based on differences in spore size. Therefore, based on nucleotide divergence and morphological similarity, *D. submersa* was synonymized with *D. tectonae* (Dong et al. 2021), *D. bambusae* was synonymized with *D. foveolata* (Delgado et al. 2025), and *D. nanchangensis* was synonymized with *D. aquatica* (Shen et al. 2025). In the present study, four synonymies are proposed for 15 *Distoseptispora* taxa, based on DNA sequence similarity, despite some differences in conidial size (Tables 1, 2). Differences in ITS may be important for identifying species in this genus.

### Challenges of studying freshwater fungi in China

China’s vast and diverse topography, encompassing plateaus, hills, karst landscapes, and plains, fosters highly heterogeneous aquatic habitats that support a unique and rich diversity of aquatic fungi (Bao et al. 2025; Xu et al. 2025). A notable geographic disparity exists in freshwater fungal records, which are mainly focused on Yunnan Province. This study helps mitigate this bias by reporting the first records of six genera and species from Jiangxi (i.e., *Dendryphiella*, *Hilberina*, *Neodendryphiella*, *Paracremonium*, *Rhamphoriopsis*, and *Wongia*).

Based on previous studies, the known distributions of these genera are as follows: species of *Dendryphiella* are currently known from *D. eucalyptorum* in Guizhou and Yunnan (Hyde et al. 2020c; Wu et al. 2024; Du et al. 2025) and *D. vinosa* in Sichuan (Hyde et al. 2020c); *Hilberina* is represented solely by *H. hongheensis*, which has only been reported from Yunnan (Yang et al. 2023); *Wongia* is documented with *W. guizhouensis* from Guizhou (Bao et al. 2025), *W. guttulata* from Guangxi (Wang et al. 2025c), *W. bambusae* from Sichuan (Yu et al. 2023), *W. miscanthi* from Taiwan (Kuo et al. 2024), and *W. aquatica*, *W. flava*, and *W. suae* from Yunnan (Luo et al. 2019; Wang et al. 2024); *Neodendryphiella* comprises *N. tarraconensis* from Guizhou (Hyde et al. 2020c) and *N. brassaiopsidis* from Yunnan (Dong et al. 2023); *Paracremonium* species have been reported from Guizhou (*P. lepidopterorum* and *P. variiforme*) (Zhang et al. 2017; Ming et al. 2021), as well as Yunnan (*P. apiculatum* and *P. ellipsoideum*) (Zhang et al. 2021); and *Rhamphoriopsis* taxa are known from Guizhou (*R. aquimicrospora*, *R. brevis*, *R. hyalospora*, and *R. sympodialis*) (Hyde et al. 2020a; Yang et al. 2023; Lin et al. 2023, 2025) and Yunnan (*R. globularis*, *R. yunnanensis*, and *R. zhaotongensis*) (Liu et al. 2025b). In contrast, three genera, *Distoseptispora*, *Pleurothecium*, and *Sporidesmiella*, exhibit broader known distributions, spanning multiple provinces such as Fujian, Guangdong, Hainan, Jiangxi, Shanxi, Sichuan, Xizang, Yunnan, and Zhejiang.

The apparent concentration of rarely recorded genera of aquatic fungi in southwestern China, primarily Yunnan and Guizhou, reflects historical research biases rather than inherent biogeographical constraints. This observation underscores the critical need for systematic, nationwide surveys of aquatic fungal diversity. Comprehensive studies on the diversity, molecular phylogeny, and ecological roles of freshwater fungi are therefore important. Such research will not only greatly enrich the global understanding of fungal biodiversity but also provide a vital scientific foundation for the conservation and sustainable use of China’s aquatic ecosystems.

## Supplementary Material

XML Treatment for Dictyosporiaceae

XML Treatment for Dendryphiella

XML Treatment for Dendryphiella loti

XML Treatment for Neodendryphiella

XML Treatment for Neodendryphiella loti

XML Treatment for Helminthosphaeriaceae

XML Treatment for Hilberina

XML Treatment for Hilberina jiangxiensis

XML Treatment for Distoseptisporaceae

XML Treatment for Distoseptispora

XML Treatment for Distoseptispora aquatica

XML Treatment for Distoseptispora clematidis

XML Treatment for Distoseptispora phangngaensis

XML Treatment for Distoseptispora tectonae

XML Treatment for Distoseptispora dinghuensis

XML Treatment for Distoseptispora quzhouensis

XML Treatment for Junewangiaceae

XML Treatment for Sporidesmiella

XML Treatment for Sporidesmiella saprophytica

XML Treatment for Sporidesmiella xishuangbannaensis

XML Treatment for Papulosaceae

XML Treatment for Wongia

XML Treatment for Wongia lignicola

XML Treatment for Wongia saprophytica

XML Treatment for Nectriaceae

XML Treatment for Paracremonium

XML Treatment for Paracremonium jiangxiense

XML Treatment for Pleurotheciaceae

XML Treatment for Pleurothecium

XML Treatment for Pleurothecium saprophyticum

XML Treatment for Rhamphoriaceae

XML Treatment for Rhamphoriopsis

XML Treatment for Rhamphoriopsis jiangxiensis

XML Treatment for Xylolentia

XML Treatment for Xylolentia oblongispora

## Additional information

### Artificial Intelligence (AI) use

The authors accept full responsibility for the content of the manuscript, including the disclosure of any use of AI.

No AI tools were used in the preparation of this manuscript.

### Adherence to national and international regulations

All the fungal strains used in this study have been legally obtained, respecting the Convention on Biological Diversity (Rio Convention).

### Author ORCIDs

L. Lu https://orcid.org/0000-0003-0977-6414

Z.-J. Zhai https://orcid.org/0009-0008-7562-9707

Y.-R. Xiong https://orcid.org/0000-0002-4673-606X

S.C. Karunarathna https://orcid.org/0000-0001-7080-0781

S. Tibpromma https://orcid.org/0000-0002-4706-6547

D.-M. Hu https://orcid.org/0000-0002-4750-2871

### Data availability

All of the data that support the findings of this study are available in the main text or Supplementary Information.

## Supplementary materials

Supplementary material 1 Tree alignment Data type zip https://binary.pensoft.net/file/1695601This dataset is made available under the Open Database License (http://opendatacommons.org/licenses/odbl/1.0/). The Open Database License (ODbL) is a license agreement intended to allow users to freely share, modify, and use this Dataset while maintaining this same freedom for others, provided that the original source and author(s) are credited. Li Lu, Si-Ping Zheng, Zhi-Jun Zhai, Yin-Ru Xiong, Samantha C. Karunarathna, Saowaluck Tibpromma, Dian-Ming Hu, Hai-Yan Song

Supplementary material 2 Additional tables Data type docx https://binary.pensoft.net/file/1695602This dataset is made available under the Open Database License (http://opendatacommons.org/licenses/odbl/1.0/). The Open Database License (ODbL) is a license agreement intended to allow users to freely share, modify, and use this Dataset while maintaining this same freedom for others, provided that the original source and author(s) are credited. Li Lu, Si-Ping Zheng, Zhi-Jun Zhai, Yin-Ru Xiong, Samantha C. Karunarathna, Saowaluck Tibpromma, Dian-Ming Hu, Hai-Yan Song
