# Supplementary material for: Multi-locus phylogenetic analyses and morphology reveal new species, new records, and taxonomic updates of saprobic Dothideomycetes and Sordariomycetes from freshwater habitats in China
Source: IMA Fungus. 2026 Jun 25;17:e188546. doi: 10.3897/imafungus.17.188546 (PMC13329429; doi:10.3897/imafungus.17.188546)
Supplement: Supplementary material 2 — Additional tables [file imafungus-17-e188546-s002.docx]

**Supplementary Table 1**. Names, voucher numbers, and corresponding GenBank numbers of the taxa used in the phylogenetic analyses of *Dendryphiella* and *Neodendryphiella*.

| Species | Voucher number | GenBank accession number | | | | References |
| --- | --- | --- | --- | --- | --- | --- |
|  |  | ITS | LSU | SSU | *TEF*1-α |  |
| *Dendryphiella eucalyptorum* | CBS 137987 | KJ869139 | KJ869196 | NA | NA | Crous et al. (2014) |
| *Dendryphiella eucalyptorum* | KUNCC:23-13926 | PQ671199 | PQ671119 | PP925657 | PQ662550 | Zhang et al. (2025) |
| *Dendryphiella fasciculata* | MFLUCC 17-1074 | MF399213 | MF399214 | NA | NA | Liu et al. (2017) |
| ***Dendryphiella loti*** | **JAUCC 8231** | **PZ136815** | **PZ136851** | **PZ136837** | **PZ150687** | **This study** |
| ***Dendryphiella loti*** | **JAUCC 8232** | **PZ136816** | **PZ136852** | **PZ136838** | **PZ150688** | **This study** |
| *Dendryphiella paravinosa* | CBS 118716 | LT963357 | LT963359 | NA | NA | Liu et al. (2017) |
| *Dendryphiella paravinosa* | CBS 121797 | LT963354 | LT963355 | NA | NA | Liu et al. (2017) |
| *Dendryphiella paravinosa* | CBS 141286 | KX228257 | KX228309 | NA | NA | Crous et al. (2016) |
| *Dendryphiella variabilis* | CBS 584.96 | LT963453 | LT963454 | NA | NA | Iturrieta-Gonzalez et al. (2018) |
| *Dendryphiella vinosa* | HUEST:23.0217 | PP925611 | PP925665 | PP925654 | PP926519 | Du et al. (2025b) |
| *Dendryphiella verrucosispora* | UESTCC 23.0504 | PQ415535 | PQ415541 | NA | PQ432236 | Du et al. (2025b) |
| *Dendryphiella verrucosispora* | UESTCC 24.0205 | PQ415539 | PQ415543 | NA | PQ432240 | Du et al. (2025b) |
| *Dendryphiella trisepta* | COAD 2388 | MK278898 | MK277357 | NA | NA | Ferreira and Barreto (2019) |
| *Dendryphiella philsamilokensis* | MFLUCC 17-2513 | MG754400 | MG754401 | MG754402 | NA | Hyde et al. (2018) |
| *Dendryphiella stromaticola* | LAMIC 90/16 | MK829079 | NA | NA | NA | Crous et al. (2019a) |
| *Jalapriya inflata* | NTOU 3855 | JQ267362 | JQ267363 | JQ267361 | NA | Kirschner et al. (2013) |
| *Jalapriya pulchra* | MFLUCC 15-0348 | KU179108 | KU179109 | KU179110 | NA | Boonmee et al. (2016) |
| *Jalapriya toruloides* | CBS 209.65 | DQ018093 | DQ018104 | DQ018081 | NA | Boonmee et al. (2016) |
| ***Neodendryphiella loti*** | **JAUCC 8233** | **PZ136817** | **PZ136853** | **PZ136839** | **NA** | **This study** |
| ***Neodendryphiella loti*** | **JAUCC 8234** | **PZ136818** | **PZ136854** | **PZ136840** | **NA** | **This study** |
| *Neodendryphiella mali* | CBS 139.95 | LT906655 | LT906657 | NA | NA | Iturrieta-Gonzalez et al. (2018) |
| *Neodendryphiella mali* | FMR 17003 | LT993734 | LT993735 | NA | NA | Iturrieta-Gonzalez et al. (2018) |
| *Neodendryphiella michoacanensis* | FMR 16098 | LT906660 | LT906658 | NA | NA | Iturrieta-Gonzalez et al. (2018) |
| *Neodendryphiella tarraconensis* | FMR 16234 | LT906659 | LT906656 | NA | NA | Iturrieta-Gonzalez et al. (2018) |
| *Neodendryphiella agapanthi* | CPC 47896 | PV664931 | PV664957 | NA | NA | Crous et al. (2025) |
| *Neodendryphiella brassaiopsidis* | ZHKUCC 23-0879 | OR365455 | OR365485 | OR365491 | NA | Dong et al. (2023) |
| *Periconia igniaria* | CBS 379.86 | NA | AB807566 | AB797276 | AB808542 | Tanaka et al. (2015) |
| *Periconia igniaria* | CBS 845.96 | NA | GU301841 | GU296171 | AB808543 | Tanaka et al. (2015) |

Newly generated sequences are in red, and “NA” indicates the sequence unavailability.

**Supplementary Table 2.** Names, voucher numbers, and corresponding GenBank numbers of the taxa used in the phylogenetic analyses of *Hilberina*.

| Species | Voucher number | GenBank accession number | | References |
| --- | --- | --- | --- | --- |
|  |  | LSU | *TUB*2 |  |
| *Chaetosphaeria ovoidea* | SMH2605 | AF064641 | NA | Fernández et al. (1999) |
| *Echinosphaeria canescens* | JHC97-006 | KF765604 | KF765622 | Miller et al. (2014) |
| *Echinosphaeria canescens* | SMH4666 | KF765605 | KF765623 | Miller et al. (2014) |
| *Helminthosphaeria carpathica* | SMH3903 | KF765606 | KF765625 | Miller et al. (2014) |
| *Helminthosphaeria odontiae* | ANM928 | KF765610 | KF765631 | Miller et al. (2014) |
| *Helminthosphaeria triseptata* | JF04015 | KF765614 | KF765636 | Miller et al. (2014) |
| *Hilberina caudata* | SMH1542 | KF765615 | KF765637 | Miller et al. (2014) |
| *Hilberina hongheensis* | HKAS 122677 | OQ379422 | OQ379003 | Yang et al. (2023b) |
| *Hilberina hongheensis* | HKAS 122678 | OQ379423 | OQ379004 | Yang et al. (2023b) |
| *Hilberina hongheensis* | MFLUCC 25-0188 | PQ608165 | NA | Yang et al. (2023b) |
| ***Hilberina jiangxiensis*** | **JAUCC 7185** | **PZ136855** | **PZ162925** | **This study** |
| ***Hilberina jiangxiensis*** | **JAUCC 7186** | **PZ136856** | **PZ162926** | **This study** |
| *Hilberina munkii* | SMH1531 | KF765616 | KF765638 | Miller et al. (2014) |
| *Hilberina punctata* | SMH4825 | NA | KF765639 | Miller et al. (2014) |
| *Hilberina robusta* | SMH3054 | NA | KF765640 | Miller et al. (2014) |
| *Hilberina sphagnorum* | Buck49156 | KF765617 | KF765641 | Miller et al. (2014) |
| *Kramasamuha sibiki* | CPC 35619 = CBS 146338 | MN794355 | NA | Hernández-Restrepo et al. (2020) |
| *Kramasamuha sibiki* | CPC 36725 = CBS 146339 | MN794356 | NA | Hernández-Restrepo et al. (2020) |
| *Kramasamuha sibiki* | CBS 146133 = CPC 36153 | MN794357 | NA | Hernández-Restrepo et al. (2020) |
| *Kramasamuha sibiki* | COAD 2632 | MN794358 | NA | Hernández-Restrepo et al. (2020) |
| *Kramasamuha* sp. | XG276a | PV578357 | NA | Xiong et al. (2026, in press) |
| *Kramasamuha* sp. | XG276b | PV578356 | NA | Xiong et al. (2026, in press) |
| *Lasiosphaeria ovina* | SMH1538 | AF064643 | NA | Fernández et al. (1999) |
| *Neurospora crassa* | MUCL 19026 | AF286411 | NA | Untereiner et al. (2001) |
| *Ruzenia spermoides* | ANM163 | KF765618 | KF765642 | Miller et al. (2014) |
| *Ruzenia spermoides* | SMH4606 | AY436422 | KF765644 | Miller and Huhndorf (2004) |
| *Ruzenia spermoides* | SMH4655 | KF765619 | KF765645 | Miller and Huhndorf (2004) |
| *Synaptospora plumbea* | ANM963 | KF765620 | KF765646 | Miller et al. (2014) |
| *Synaptospora plumbea* | SMH3962 | KF765621 | KF765647 | Miller et al. (2014) |
| *Selenosporella curvispora* | CBS 102623 | MW144405 | NA | Réblová et al. (2021) |
| *Sporoschisma hemipsila* | SMH2125 | AY346292 | NA | Huhndorf et al. (2004) |

Newly generated sequences are in red, and “NA” indicates the sequence unavailability.

**Supplementary Table 3**. Names, voucher numbers, and corresponding GenBank numbers of the taxa used in the phylogenetic analyses of *Sporidesmiella*.

| Species | Voucher number | GenBank accession number | | | | References |
| --- | --- | --- | --- | --- | --- | --- |
|  |  | ITS | LSU | *TEF*1-α | *RPB*2 |  |
| *Cancellidium cinereum* | MFLUCC 18-0424 | MT370353 | MT370363 | NA | MT370486 | Hyde et al. (2021a) |
| *Dictyosporella aquatica* | CBS H-22127 | NA | KT241022 | NA | NA | Ariyawansa et al. (2015) |
| *Dictyosporella chiangmaiensis* | MFLUCC 17-2345 | MW286491 | MW287765 | NA | NA | Dong et al. (2021a) |
| *Dictyosporella ellipsoidea* | MFLUCC 18-1042 | NA | MW287758 | NA | NA | Dong et al. (2021a) |
| *Dictyosporella guizhouensis* | MFLU 18-1505 | MK593606 | MK593605 | NA | NA | Yuan et al. (2020) |
| *Dictyosporella guizhouensis* | MFLUCC 18-1232 | MW286487 | MW287760 | MW396646 | NA | Yuan et al. (2020) |
| *Dictyosporella hydei* | IFRDCC 3075 | NA | MG813161 | NA | NA | Song et al. (2018) |
| *Dictyosporella thailandensis* | MFLUCC 15-0985 | MF374355 | MF374364 | MF370958 | MF370952 | Zhang et al. (2017a) |
| *Junewangia aquatic* | HFJAU 0700 | MG213738 | MG213737 | NA | NA | Song et al. (2018) |
| *Junewangia globulosa* | CBS 126093 | MH864078 | MH875535 | NA | NA | Vu et al. (2019) |
| *Junewangia lamma* | HSAUPH 4695 | KU999971 | KU751883 | NA | NA | Ma (2012) |
| *Junewangia lamma* | HMAS 44438 | KU999961 | KU751882 | NA | NA | Ma (2012) |
| *Junewangia queenslandica* | HSAUPH 7722 | KU999984 | KX033575 | NA | NA | Ma (2012) |
| *Junewangia sphaerospora* | HSAUPH 4733 | KU999981 | KX033572 | NA | NA | Ma (2012) |
| *Junewangia thailandica* | MFLU 15-2682 | NA | MW287762 | NA | NA | Dong et al. (2021a) |
| *Sporidesmiella aquatica* | S-1339 | NA | MK849844 | MN194035 | MN124524 | Luo et al. (2019) |
| *Sporidesmiella aquatica* | S-777 | MK828692 | MK849843 | MN194034 | NA | Luo et al. (2019) |
| *Sporidesmiella dujuanhuensis* | KUNCC 23-13445 | PQ845942 | PV536389 | PX238291 | PX233794 | Shen et al. (2025) |
| *Sporidesmiella dulongjiangensis* | KUNCC 23-16480 | PQ415506 | PQ415505 | PQ587329 | PQ587328 | Shen et al. (2025) |
| *Sporidesmiella guttulata* | KUNCC 23-14231 | PQ845945 | PV536392 | PX238294 | PX233797 | Shen et al. (2025) |
| *Sporidesmiella guttulata* | KUNCC 23-14257 | PQ845946 | PV536393 | PX238295 | PX233798 | Shen et al. (2025) |
| *Sporidesmiella guttulata* | KUNCC 23-13416 | PQ845943 | PV536390 | PX238292 | PX233795 | Shen et al. (2025) |
| *Sporidesmiella guttulata* | KUNCC 23-13431 | PQ845944 | PV536391 | PX238293 | PX233796 | Shen et al. (2025) |
| *Sporidesmiella hongheensis* | KUNCC 23-13444 | PQ845947 | PV536394 | PX238296 | PX233799 | Shen et al. (2025) |
| *Sporidesmiella hyalosperma* | MFLUCC 18-1013 | MW286499 | MW287773 | MW396654 | MW504070 | Crous et al. (2020) |
| *Sporidesmiella hyalosperma* | CPC 37552 | MT223845 | MT223934 | NA | MT223705 | Crous et al. (2020) |
| *Sporidesmiella hydei* | KUNCC 23-14293 | PQ845949 | PV536396 | PX238298 | PX233801 | Shen et al. (2025) |
| *Sporidesmiella junci* | CBS 149443 | OP675893 | OP681182 | NA | OP676106 | Tan et al. (2022) |
| *Sporidesmiella juncicola* | CPC 41075 | OK664718 | OK663757 | NA | OK651165 | Crous et al. (2021a) |
| *Sporidesmiella juncicola* | CPC 41109 | OK664719 | OK663758 | NA | OK651166 | Crous et al. (2021a) |
| *Sporidesmiella lacustris* | KUNCC 23-14747 | PQ845951 | PV536398 | PX238300 | PX233803 | Shen et al. (2025) |
| *Sporidesmiella lacustris* | KUNCC 23-14751 | PQ845952 | PV536399 | PX238301 | PX233804 | Shen et al. (2025) |
| *Sporidesmiella lignicola* | JAUCC 3436 | MZ613187 | OK091615 | OK323223 | OK323222 | Li et al. (2021) |
| *Sporidesmiella motuoensis* | KUNCC 10425 | OP626348 | OR229720 | NA | NA | Xiong et al. (2024) |
| *Sporidesmiella novae-zelandiae* | S-951 | MK828695 | MK849847 | MN194037 | MN124526 | Luo et al. (2019) |
| *Sporidesmiella novae-zelandiae* | S-048 | MK828694 | MK849846 | NA | NA | Luo et al. (2019) |
| *Sporidesmiella novae-zelandiae* | S-1256 | MK828693 | MK849845 | NA | NA | Luo et al. (2019) |
| *Sporidesmiella obovoidia* | MFLUCC 17-2372 | MW286492 | MW287766 | NA | NA | Dong et al. (2021a) |
| *Sporidesmiella obovoidispora* | GZAAS 24-0048 T | PP657290 | PP657332 | NA | NA | Liu et al. (2024a) |
| *Sporidesmiella pini* | CPC 40067 | OK664747 | OK663786 | NA | OK651177 | Crous et al. (2023a) |
| *Sporidesmiella sichuanensis* | HKAS 136267 | PQ038263 | PQ038270 | PQ050356 | PQ050359 | Tian et al. (2024) |
| *Sporidesmiella* sp. | S3-1 | OL435559 | ON124909 | NA | NA | NCBI |
| *Sporidesmiella* sp. | SN31 | OL435557 | ON124907 | NA | NA | NCBI |
| *Sporidesmiella* sp. | SN23 | OL435556 | ON124906 | NA | NA | NCBI |
| *Sporidesmiella* sp. | S1-13 | OL435558 | ON124908 | NA | NA | NCBI |
| *Sporidesmiella* sp. | SN19 | OL435555 | ON124905 | NA | NA | NCBI |
| ***Sporidesmiella saprophytica*** | **JAUCC7440** | **PZ136821** | **PZ136857** | **NA** | **PZ137506** | **This study** |
| ***Sporidesmiella saprophytica*** | **JAUCC7441** | **PZ136822** | **PZ136858** | **NA** | **PZ137507** | **This study** |
| *Sporidesmiella xishuangbannaensis* | KUNCC 25-19462 | PV899933 | PV899934 | NA | NA | Qian et al. (2026, in press) |
| ***Sporidesmiella xishuangbannaensis*** | **JAUCC 7022** | **PZ136823** | **PZ136859** | **PZ150689** | **PZ137508** | **This study** |
| ***Sporidesmiella xishuangbannaensis*** | **JAUCC 7447** | **PZ136824** | **PZ136860** | **PZ150690** | **PZ137509** | **This study** |
| *Sporidesmiella yadongensis* | KUNCC 24-17996 | PQ168239 | PQ152625 | NA | NA | Shen et al. (2025) |
| *Sporidesmiella yunnanensis* | KUNCC 24-18359 | PQ346794 | PQ346791 | NA | NA | Shen et al. (2025) |
| *Sporidesmiella yunnanensis* | KUNCC 23-14751 | PQ845952 | PV536399 | PX238301 | PX233804 | Shen et al. (2025) |
| *Sporidesmiella yunnanensis* | HKAS 131657 | PQ845741 | PQ819689 | NA | NA | Shen et al. (2025) |

Newly generated sequences are in red, and “NA” indicates the sequence unavailability.

**Supplementary Table 4**. Names, voucher numbers, and corresponding GenBank numbers of the taxa used in the phylogenetic analyses of *Wongia*.

| Species | Voucher number | GenBank accession number | | | | | References |
| --- | --- | --- | --- | --- | --- | --- | --- |
|  |  | ITS | LSU | SSU | *RPB*2 | *TEF*1-α |  |
| *Brunneosporella aquatica* | HKUCC 3708 | [AF177154](http://www.ncbi.nlm.nih.gov/nuccore/AF177154) | [AF132326](http://www.ncbi.nlm.nih.gov/nuccore/AF132326) | NA | NA | NA | Ranghoo et al. (2000) |
| *Cancellidium atrobrunneum* | MFLUCC 20-0100 | [MT422724](http://www.ncbi.nlm.nih.gov/nuccore/MT422724) | [MT422740](http://www.ncbi.nlm.nih.gov/nuccore/MT422740) | [MT422726](http://www.ncbi.nlm.nih.gov/nuccore/MT422726) | NA | [MT436438](http://www.ncbi.nlm.nih.gov/nuccore/MT436438) | Hyde et al. (2021a) |
| *Cancellidium cinereum* | MFLUCC 18-0424 | [MT370353](http://www.ncbi.nlm.nih.gov/nuccore/MT370353) | [MT370363](http://www.ncbi.nlm.nih.gov/nuccore/MT370363) | [MT370351](http://www.ncbi.nlm.nih.gov/nuccore/MT370351) | [MT370486](http://www.ncbi.nlm.nih.gov/nuccore/MT370486) | [MT370488](http://www.ncbi.nlm.nih.gov/nuccore/MT370488) | Hyde et al. (2021a) |
| *Cancellidium griseonigrum* | MFLUCC 17-2117 | [MT370354](http://www.ncbi.nlm.nih.gov/nuccore/MT370354) | [MT370364](http://www.ncbi.nlm.nih.gov/nuccore/MT370364) | [MT370352](http://www.ncbi.nlm.nih.gov/nuccore/MT370352) | [MT370487](http://www.ncbi.nlm.nih.gov/nuccore/MT370487) | NA | Hyde et al. (2021a) |
| *Fluminicola aquatica* | MFLUCC 15-0962 | MF374357 | MF374366 | MF374374 | NA | MF370960 | Hyde et al. (2021a) |
| *Fluminicola saprophytica* | MFLUCC 15-0976 | NR_153493 | MF374367 | MF374375 | MF370954 | MF370956 | Hyde et al. (2021a) |
| *Fluminicola striata* | MFLUCC 18-0990 | MW286496 | MW287770 | NA | NA | NA | Dong et al. (2021a) |
| *Papulosa amerospora* | AFTOL-ID 748 | NA | [DQ470950](http://www.ncbi.nlm.nih.gov/nuccore/DQ470950) | [DQ470998](http://www.ncbi.nlm.nih.gov/nuccore/DQ470998) | [DQ470901](http://www.ncbi.nlm.nih.gov/nuccore/DQ470901) | [DQ471069](http://www.ncbi.nlm.nih.gov/nuccore/DQ471069) | Spatafora et al. (2006) |
| *Pseudostanjehughesia aquitropica* | MFLUCC 16-0569 | [MF077548](http://www.ncbi.nlm.nih.gov/nuccore/MF077548) | [MF077559](http://www.ncbi.nlm.nih.gov/nuccore/MF077559) | [MF077537](http://www.ncbi.nlm.nih.gov/nuccore/MF077537) | NA | [MF135655](http://www.ncbi.nlm.nih.gov/nuccore/MF135655) | Yang et al. (2018) |
| *Pseudostanjehughesia lignicola* | MFLUCC 15-0352 | [MK828643](http://www.ncbi.nlm.nih.gov/nuccore/MK828643) | [MK849787](http://www.ncbi.nlm.nih.gov/nuccore/MK849787) | NA | [MN124534](http://www.ncbi.nlm.nih.gov/nuccore/MN124534) | [MN194047](http://www.ncbi.nlm.nih.gov/nuccore/MN194047) | Luo et al. (2019) |
| *Platytrachelon abietis* | CBS 125235 | NA | [JX066703](http://www.ncbi.nlm.nih.gov/nuccore/JX066703) | [JX066707](http://www.ncbi.nlm.nih.gov/nuccore/JX066707) | [JX066698](http://www.ncbi.nlm.nih.gov/nuccore/JX066698) | NA | Réblová (2013) |
| *Wongia pallidopolaris* | CBS 440.70 | PX283740 | PX283750 | PX283744 | PX310216 | PX310208 | Réblová et al. (2025a) |
| *Wongia rhachidophora* | CBS 531.73 | PX283741 | PX283751 | PX283745 | PX310217 | PX310209 | Réblová et al. (2025a) |
| *Wongia aquatica* | MFLUCC 18-1607 | MK828645 | MK849788 | MK828312 | MN124536 | MN194048 | Luo et al. (2019) |
| *Wongia aquatica* | GZCC 25–0682 | PV982851 | PV982831 | PV982865 | PV987743 | PX312464 | Bao et al. (2025) |
| *Wongia aquatica* | GZCC 25–0683 | PV982853 | NA | NA | PV987745 | NA | Bao et al. (2025) |
| *Wongia bambusae* | CGMCC 3.24360 | OR822001 | OR822017 | OR822010 | OR862129 | OR873427 | Yu et al. (2024a) |
| *Wongia bambusae* | KUNCC 24-17699 | PQ571131 | PQ573785 | PQ571125 | PQ591896 | PQ591892 | Wang et al. (2025) |
| *Wongia bandungensis* | TBRC-BCC 95171 | OQ121929 | OQ121947 | OQ121938 | OQ116752 | OQ116761 | Manawasinghe et al. (2025) |
| *Wongia bandungensis* | TBRC-BCC 95343 | OQ121930 | OQ121948 | OQ121939 | OQ116753 | OQ116762 | Manawasinghe et al. (2025) |
| *Wongia ficherai* | BRIP 69019 | OM230139 | OM230140 | NA | OM162025 | NA | Crous et al. (2022) |
| *Wongia flava* | CGMCC 3.25434 | OR589341 | OR769700 | OR743229 | OR820915 | OR739187 | Wang et al. (2024) |
| *Wongia fusiformis* | DLUCC 1767 | MZ420746 | MZ420761 | MZ420750 | NA | NA | Bao et al. (2021) |
| *Wongia fusiformis* | KUNCC 23-16632 | PQ571134 | PQ573788 | PQ571128 | PQ591898 | PQ591895 | Wang et al. (2024) |
| *Wongia fusiformis* | MFLUCC 21-0028 | MZ412517 | MZ412529 | MZ413273 | NA | MZ442690 | Bao et al. (2021) |
| *Wongia fusiformis* | MFLUCC 21-0032 | MZ412515 | MZ412527 | MZ413271 | NA | MZ442689 | Bao et al. (2021) |
| *Wongia garrettii* | DAR 79637 | KU850474 | NA | NA | NA | KU850467 | Khemmuk et al. (2016) |
| *Wongia grifinii* | BRIP 60377 | KU850472 | KU850470 | NA | NA | KU850466 | Khemmuk et al. (2016) |
| *Wongia grifinii* | DAR 80512 | KU850473 | KU850471 | NA | NA | NA | Khemmuk et al. (2016) |
| *Wongia guttulata* | KUNCC 24-17692 | PQ571135 | PQ573789 | NA | PQ591899 | NA | Wang et al. (2024) |
| ***Wongia lignicola*** | **JAUCC7164** | **PZ136827** | **PZ136863** | **PZ136843** | **NA** | **PZ150693** | **This study** |
| ***Wongia lignicola*** | **JAUCC7445** | **PZ136828** | **PZ136864** | **PZ136844** | **NA** | **PZ150694** | **This study** |
| *Wongia miscanthi* | BCRC FU32062 | LC822730 | LC822732 | LC822736 | LC822734 | LC822738 | Kuo et al. (2024) |
| ***Wongia saprophytica*** | **JAUCC7070** | **PZ136825** | **PZ136861** | **PZ136841** | **NA** | **PZ150691** | **This study** |
| ***Wongia saprophytica*** | **JAUCC7177** | **PZ136826** | **PZ136862** | **PZ136842** | **NA** | **PZ150692** | **This study** |
| *Wongia suae* | CGMCC 3.24295 | OQ911478 | OQ911483 | OQ998925 | OR039047 | OR039046 | Zhang et al. (2023) |
| *Wongia suae* | GZCC 25–0687 | PV982854 | PV982833 | PV982866 | PV987746 | NA | Bao et al. (2025) |
| *Wongia guizhouensis* | GZCC 25–0684 | PV982850 | PV982830 | PV982864 | PV987742 | PX312463 | Bao et al. (2025) |
| *Wongia guizhouensis* | GZCC 25–0685 | PV982849 | PV982829 | NA | PV987741 | NA | Bao et al. (2025) |
| *Wongia guizhouensis* | GZCC 25–0686 | PV982852 | PV982832 | NA | PV987744 | PX312465 | Bao et al. (2025) |

Newly generated sequences are in red, “–” indicates the sequence unavailability.

**Supplementary Table 5**. Names, voucher numbers, and corresponding GenBank numbers of the taxa used in the phylogenetic analyses of *Paracremonium.*

| Species | Voucher number | GenBank accession number | | | | | Reference |
| --- | --- | --- | --- | --- | --- | --- | --- |
|  |  | ITS | LSU | *TEF*1-α | *RPB*2 | *TUB*2 |  |
| *Nalanthamala vermoesenii* | CBS 230.48 | NR_145023 | NG_057727 | KM231970 | KM232399 | AY554231 | Schroers et al. (2005) |
| *Nalanthamala vermoesenii* | CBS 110893 | AY554214 | AY554246 | KM231971 | KM232400 | AY554233 | Schroers et al. (2005) |
| *Nalanthamala psidii* | CBS 687.97 | AY554208 | AY554255 | NA | NA | AY554227 | Schroers et al. (2005) |
| *Paracremonium apiculatum* | LC 12502 | MK329124 | MK329029 | MK336059 | NA | MK336137 | Zhang et al. (2021) |
| *Paracremonium apiculatum* | CGMCC 3.19309 | MK329123 | MK329028 | MK336058 | NA | MK336136 | Zhang et al. (2021) |
| *Paracremonium aquaticum* | MFLUCC 22-0120 | OP216410 | OP216405 | OP251195 | OP251199 | OP251200 | Liu et al. (2024b) |
| *Paracremonium aquaticum* | CBS 482.78 | KM231830 | KM231711 | KM231965 | KM232395 | KM232102 | Lombard et al. (2015) |
| *Paracremonium bendijkiorum* | CBS 147228 | MW883436 | MW883828 | MW890111 | MW890068 | MW890139 | Crous et al. (2021b) |
| *Paracremonium binnewijzendii* | CBS 143277 | NR_157491 | NG_063950 | NA | NA | NA | Crous et al. (2017) |
| *Paracremonium binnewijzendii* | TUCIM 10280 | MT217127 | NA | NA | NA | NA | Ding et al. (2021) |
| *Paracremonium contagium* | CBS 110348 | KM231831 | HQ232118 | KM231966 | KM232396 | KM232103 | Lombard et al. (2015) |
| *Paracremonium ellipsoideum* | CGMCC 3.19316 | MK329125 | MK329030 | MK336060 | NA | MK336138 | Zhang et al. (2021) |
| *Paracremonium ellipsoideum* | LC 12552 | MK329126 | MK329031 | MK336061 | NA | MK336139 | Zhang et al. (2021) |
| *Paracremonium inflatum* | CBS 485.77 | KM231829 | HQ232113 | KM231964 | KM232394 | KM232101 | Lombard et al. (2015) |
| ***Paracremonium jiangxiense*** | **JAUCC7045** | **PZ136829** | **PZ136865** | **PZ150695** | **PZ137512** | **PZ162927** | **This study** |
| ***Paracremonium jiangxiense*** | **JAUCC7046** | **PZ136830** | **PZ136866** | **PZ150696** | **PZ137513** | **PZ162928** | **This study** |
| *Paracremonium lepidopterorum* | DY 10351 | MW000352 | MW000470 | NA | NA | MW015086 | Ding et al. (2021) |
| *Paracremonium lepidopterorum* | DY 10352 | MW000462 | MW000667 | NA | NA | MW015087 | Ding et al. (2021) |
| *Paracremonium moubasheri* | AUMC 11030 | KX384655 | NA | NA | NA | NA | Al-Bedak et al. (2019) |
| *Paracremonium pembeum* | UCRCFU 238 | KP012601 | KP012621 | KP012641 | NA | NA | Lynch et al. (2016) |
| *Paracremonium pembeum* | UCRCFU 258 | KP012605 | KP012625 | KP012645 | NA | NA | Lynch et al. (2016) |
| *Paracremonium variiforme* | CGMCC 3.17931 | KU746691 | KU746737 | KX855237 | NA | KU746783 | Zhang et al. (2017b) |
| *Paracremonium variiforme* | CGMCC 3.17932 | KU746692 | KU746738 | KX855238 | NA | KU746784 | Zhang et al. (2017b) |
| *Paracremonium variiforme* | CGMCC 3.17933 | KU746693 | KU746739 | KX855239 | NA | KU746785 | Zhang et al. (2017b) |

Newly generated sequences are in red, “NA” indicates the sequence unavailability.

**Supplementary Table 6**. Names, voucher numbers, and corresponding GenBank numbers of the taxa used in the phylogenetic analyses of *Pleurothecium*.

| Species | Voucher number | GenBank accession number | | | | References |
| --- | --- | --- | --- | --- | --- | --- |
|  |  | ITS | LSU | *SSU* | *RPB*2 |  |
| *Adelosphaeria catenata* | CBS 138679 | KT278721 | KT278707 | KT278692 | KT278743 | Réblová et al. (2016) |
| *Anapleurothecium botulisporum* | FMR 11490 | KY853423 | KY853483 | NA | NA | Hernández-Restrepo et al. (2017) |
| *Canalisporium exiguum* | SS 00809 | GQ390296 | GQ390281 | GQ390266 | HQ446436 | Boonyuen et al. (2011) |
| *Canalisporium grenadoideum* | SS 03615 | NA | GQ390267 | GQ390252 | HQ446420 | Sri-indrasutdhi et al. (2010) |
| *Canalisporium pulchrum* | SS 03982 | GQ390292 | GQ390277 | GQ390262 | HQ446432 | Boonyuen et al. (2011) |
| *Coleodictyospora muriformis* | MFLUCC 18-1243 | MW981642 | MW981648 | MW981704 | NA | Dong et al. (2021a) |
| *Coleodictyospora muriformis* | MFLUCC 18-1279 | MW981643 | MW981649 | MW981705 | NA | Dong et al. (2021a) |
| *Conioscypha lignicola* | CBS 335.93 | NA | AY484513 | JQ437439 | JQ429260 | Réblová et al. (2012) |
| *Conioscypha minutispora* | CBS 137253 | KF924559 | MH878131 | HF937347 | NA | Vu et al. (2019) |
| *Dematipyriforma aquilaria* | CGMCC 3.17268 | KJ138621 | KJ138623 | KJ138622 | NA | Sun et al. (2017) |
| *Dematipyriforma muriformis* | MFLU 21-0146 | OM654773 | OM654770 | NA | NA | Bao et al. (2022) |
| *Dematipyriforma nigrospora* | MFLUCC 21-0096 | MZ538524 | MZ538558 | NA | NA | Bao et al. (2022) |
| *Dematipyriforma nigrospora* | MFLUCC 21-0097 | MZ538525 | MZ538559 | MZ538574 | MZ567113 | Bao et al. (2022) |
| *Helicoascotaiwania farinosa* | DAOMC 241947 | JQ429145 | JQ429230 | NA | NA | Réblová et al. (2020) |
| *Helicoascotaiwania lacustris* | CBS 145963 | NA | MN699430 | MN699382 | MN704304 | Réblová et al. (2020) |
| *Helicoascotaiwania lacustris* | CBS 146144 | MN699401 | MN699432 | MN699384 | MN704306 | Réblová et al. (2020) |
| *Melanotrigonum ovale* | CBS 138742 | KT278723 | KT278708 | KT278695 | KT278744 | Réblová et al. (2016) |
| *Melanotrigonum ovale* | CBS 138743 | KT278724 | KT278709 | KT278696 | KT278745 | Réblová et al. (2016) |
| *Monotosporella seteosa* | HKUCC 3713 | NA | AF132334 | NA | NA | Réblová et al. (2020) |
| *Nenascotaiwania fusiformis* | MFLU 15–1156 | MG388215 | NG_057114 | NA | NA | Réblová et al. (2020) |
| *Neoascotaiwania fusiformis* | MFLUCC 15–0625 | NA | KX550894 | KX550898 | NA | Réblová et al. (2020) |
| *Neomonodictys aquatica* | KUNCC 21–10708 | MZ686200 | OK245417 | NA | NA | Huang et al. (2022) |
| *Neomonodictys muriformis* | MFLUCC 16-1136 | MN644509 | MN644485 | NA | NA | Hyde et al. (2020b) |
| *Phaeoisaria annesophieae* | CBS 143235 | MG022180 | MG022159 | NA | NA | Crous et al. (2017) |
| *Phaeoisaria annesophieae* | MFLU 19-0531 | MT559109 | MT559084 | NA | NA | Crous et al. (2017) |
| *Phaeoisaria aquatica* | MFLUCC 16-1298 | MF399237 | MF399254 | NA | MF401406 | Luo et al. (2018) |
| *Phaeoisaria dalbergiae* | CPC 39540 | OK664703 | OK663742 | OK663796 | OK651159 | Crous et al. (2021a) |
| *Phaeoisaria ellipsoidea* | IFRDCC 3134 | ON533383 | ON533387 | NA | NA | Yang et al. (2023c) |
| *Phaeoisaria fasciculata* | CBS 127885 | KT278719 | KT278705 | KT278693 | KT278741 | Réblová et al. (2016) |
| *Phaeoisaria filiformis* | MFLUCC 18-0214 | MK878381 | MK835852 | MK834785 | NA | Luo et al. (2019) |
| *Phaeoisaria goiasensis* | FCCUFG 02 | MT210320 | MT375865 | NA | NA | Jayawardena et al. (2022) |
| *Phaeoisaria synnematicus* | NFCCI 4479 | MK391494 | MK391492 | NA | NA | Boonmee et al. (2021) |
| *Phragmocephala stemphylioides* | DAOM 673211 | KT278730 | KT278717 | NA | NA | Réblová et al. (2016) |
| *Pleurotheciella aquatica* | MFLUCC 17–0464 | MF399236 | MF399253 | MF399220 | MF401405 | Luo et al. (2018) |
| *Pleurotheciella brachyspora* | CGMCC 3.25435 | OR589321 | OR600969 | PP049532 | PP068773 | Wang et al. (2024) |
| *Pleurotheciella centenaria* | DAOM 229631 | JQ429151 | JQ429234 | JQ429246 | JQ429265 | Réblová et al. (2012) |
| *Pleurotheciella dimorphospora* | KUMCC 20-0185 | MW981446 | MW981444 | MW981454 | MZ509665 | Boonmee et al. (2021) |
| *Pleurotheciella erumpens* | CBS 142447 | MN699406 | MN699435 | MN699387 | MN704311 | Réblová et al. (2020) |
| *Pleurotheciella fusiformis* | MFLUCC 17–0113 | MF399233 | MF399250 | MF399218 | MF401403 | Luo et al. (2018) |
| *Pleurotheciella guttulata* | KUMCC 15-0296 | MF399240 | MF399257 | MF399223 | MF401409 | Luo et al. (2018) |
| *Pleurothecium aquaticum* | MFLUCC 17-1331 | MF399245 | MF399263 | NA | NA | Luo et al. (2018) |
| *Pleurothecium aquaticum* | MFLUCC 21–0148 | OM654775 | OM654772 | OM654807 | NA | Luo et al. (2018) |
| *Pleurothecium aquisubtropicum* | GZCC 21-0670 | OM339436 | OM339433 | NA | NA | Jayawardena et al. (2022) |
| *Pleurothecium aseptatum* | GZCC 22–2019 | OQ002375 | OQ002372 | NA | NA | Liu et al. (2024b) |
| *Pleurothecium brunius* | AD291640 | OQ799373 | OQ799347 | OQ799346 | NA | Fryar and Catcheside (2023) |
| *Pleurothecium floriforme* | MFLUCC 15-0628 | KY697281 | KY697277 | KY697279 | NA | Hyde et al. (2017) |
| *Pleurothecium guttulatum* | KMUCC 20-0152 | MT555415 | MT559115 | MT559089 | NA | Shi et al. (2021) |
| *Pleurothecium hainanense* | GZCC 22-2021 | OP748934 | OP748931 | NA | NA | Hyde et al. (2023) |
| *Pleurothecium jiangxiense* | JAUCC 6077 | OR853415 | OR853420 | OR853425 | PP078757 | He et al. (2024) |
| *Pleurothecium jiangxiense* | JAUCC6676 | PP800190 | PP800212 | PP801260 | PP816288 | He et al. (2024) |
| *Pleurothecium lignicola* | JAUCC 7034 | PQ443962 | PQ443974 | PQ444007 | PQ483194 | Xu et al. (2025) |
| *Pleurothecium lignicola* | JAUCC 7035 | PQ443963 | PQ443975 | PQ444008 | NA | Xu et al. (2025) |
| *Pleurothecium obovoideum* | CBS 209.95 | EU041784 | EU041841 | NA | NA | Arzanlou et al. (2007) |
| *Pleurothecium pulneyense* | MFLUCC 16-1293 | NA | MF399262 | MF399228 | MF401414 | Arzanlou et al. (2007) |
| *Pleurothecium recurvatum* | CBS 138747 | KT278728 | KT278714 | KT278703 | NA | Réblová et al. (2016) |
| *Pleurothecium recurvatum* | CBS 131272 | JQ429149 | JQ429237 | JQ429251 | JQ429268 | Réblová et al. (2012) |
| ***Pleurothecium saprophyticum*** | **JAUCC7170** | **PZ136831** | **PZ136867** | **PZ136845** | **PZ137514** | **This study** |
| ***Pleurothecium saprophyticum*** | **JAUCC7175** | **PZ136832** | **PZ136868** | **PZ136846** | **PZ137515** | **This study** |
| *Pleurothecium semifecundum* | CBS 131271 | JQ429159 | JQ429240 | JQ429254 | JQ429270 | Réblová et al. (2012) |
| *Pleurothecium semifecundum* | CBS 131482 | JQ429158 | JQ429239 | JQ429253 | NA | Réblová et al. (2012) |
| *Pleurothecium takense* | TBRC-BCC 95074 | OQ121931 | OQ121949 | OQ121940 | OQ116754 | Manawasinghe et al. (2025) |
| *Pleurothecium takense* | TBRC-BCC 95075 | OQ121932 | OQ121950 | OQ121941 | OQ116755 | Manawasinghe et al. (2025) |
| *Pleurothecium hyalosporum* | DS 1-29 | PQ898744 | PQ898780 | PQ898814 | NA | Lin et al. (2025) |
| *Pleurothecium pisiforme* | KUNCC 24-19085 | PV264837 | PV264846 | PV335238 | NA | Wang et al. (2025) |
| *Rhexoacrodictys chiangraiensis* | MFLUCC 23-0264 | OR912106 | OR912107 | OR912108 | OR991238 | Win et al. (2025) |
| *Rhexoacrodictys melanospora* | KUNCC 22-12406 | OP168085 | OP168087 | OP168088 | OP208807 | Bao et al. (2023) |
| *Saprodesmium dematiosporium* | KUMCC 18-0059 | MW981646 | MW981647 | MW981707 | NA | Dong et al. (2021a) |
| *Sterigmatobotrys uniseptata* | MFLUCC 15-0358 | MK878379 | MK835850 | MK834784 | NA | Luo et al. (2019) |

Newly generated sequences are in red, and “NA” indicates the sequence unavailability.

**Supplementary Table 7**. Names, voucher numbers, and corresponding GenBank numbers of the taxa used in the phylogenetic analyses of *Rhamphoriopsis* and *Xylolentia*.

| Species | Voucher number | GenBank accession number | | | | | References |
| --- | --- | --- | --- | --- | --- | --- | --- |
|  |  | ITS | LSU | SSU | *TEF*1-α | *RPB2* |  |
| *Myrmecridium sorbicola* | CBS 143433 | MH107901 | MH107948 | NA | NA | NA | Crous et al. (2018) |
| *Myrmecridium schulzeri* | CBS 100.54 | EU041769 | EU041826 | NA | NA | NA | Arzanlou et al. (2007) |
| *Rhamphoriopsis aquimicrospora* | GZCC 20-0515 | OP377812 | OP377911 | OP377996 | OP472992 | OP473085 | Yang et al. (2023a) |
| *Rhamphoriopsis brevis* | MFLU 25-0021 | PQ898747 | PQ898783 | PQ898817 | PV040799 | NA | Lin et al. (2025) |
| *Rhamphoriopsis glauca* | CBS 480.75 | NA | MH872702 | NA | NA | NA | Yang et al. (2023a) |
| *Rhamphoriopsis hyalospora* | MFLU 19-2849 | MN846344 | MN846342 | NA | NA | NA | Lin et al. (2023) |
| *Rhamphoriopsis muriformis* | CBS 131269 | NA | MG600396 | MG600404 | NA | MG600400 | Réblová and Štěpánek (2018) |
| *Rhamphoriopsis muriformis* | CBS 127683 | MG600389 | MG600395 | MG600403 | NA | MG600399 | Réblová and Štěpánek (2018) |
| *Rhamphoriopsis sympodialis* | GZCC 18-0095 | MT079187 | MT079191 | NA | NA | NA | Hyde et al. (2020b) |
| *Rhamphoriopsis synnematosa* | CPC 45231 | OR680773 | OR717029 | NA | NA | OR683730 | Crous et al. (2023b) |
| *Rhamphoriopsis aquimicrospora* | HKAS 105131 | PQ898763 | PQ898799 | PQ898831 | PV040814 | NA | Lin et al. (2025) |
| *Rhamphoriopsis aquimicrospora* | HKAS 105139 | PQ898766 | PQ898802 | PQ898833 | PV040816 | NA | Lin et al. (2025) |
| *Rhamphoriopsis hyalospora* | HKAS 105140 | NA | PQ898803 | NA | PV040817 | NA | Lin et al. (2025) |
| *Rhamphoriopsis hyalospora* | HKAS 105149 | PQ898769 | PQ898806 | PQ898836 | PV040819 | NA | Lin et al. (2025) |
| *Rhamphoriopsis cuprea* | CBS 147991 | PV455942 | PV455956 | PV455969 | PV483440 | PV483456 | Réblová et al. (2025b) |
| *Rhamphoriopsis denticulata* | CBS 147996 | PV455943 | PV455957 | PV455970 | PV483441 | PV483457 | Réblová et al. (2025b) |
| *Rhamphoriopsis globularis* | GMBC5313 | PV933623 | PV933641 | PV933658 | PX392348 | PX373386 | Du et al. (2025b) |
| *Rhamphoriopsis globularis* | GMBC5314 | PV933624 | PV933642 | PV933659 | PX392349 | PX373387 | Du et al. (2025b) |
| *Rhamphoriopsis yunnanensis* | GMBC6917 | PV939404 | PV939432 | PV939456 | PX392366 | PX373388 | Habib et al. (2025) |
| *Rhamphoriopsis yunnanensis* | GMBC6919 | PV939405 | PV939433 | PV939457 | PX392367 | PX373389 | Habib et al. (2025) |
| *Rhamphoriopsis zhaotongensis* | GMBC6918 | PV939406 | PV939434 | PV939458 | PX392368 | PX373390 | Habib et al. (2025) |
| *Rhamphoriopsis zhaotongensis* | GMBC6920 | PV939407 | PV939435 | PV939459 | PX392369 | PX373391 | Habib et al. (2025) |
| *Rhodoveronaea aquatica* | MFLUCC 18-1339 | MK828641 | MK849785 | MK828310 | MN194046 | NA | Luo et al. (2019b) |
| *Rhodoveronaea aquatica* | GZCC 20-0447 | OP377862 | OP377947 | OP378027 | OP473041 | OP473107 | Yang et al. (2023a) |
| *Rhodoveronaea everniae* | CBS 148309 | OK664737 | OK663776 | NA | NA | OK651172 | Crous et al. (2021a) |
| *Rhodoveronaea hainanensis* | GZCC 22-2020 | OP748935 | OP748932 | NA | NA | NA | Hyde et al. (2023) |
| *Rhodoveronaea hyalina* | GZCC 23-0622 | PP102206 | PP102207 | PP102214 | PP259403 | PP259399 | Chen et al. (2024) |
| *Rhodoveronaea hyalina* | GZCC 23-0623 | PP102211 | PP102208 | PP102215 | PP259404 | PP259400 | Chen et al. (2024) |
| ***Rhamphoriopsis jiangxiensis*** | **JAUCC7167** | **PZ136833** | **PZ136869** | **PZ136847** | **NA** | **PZ137516** | **This study** |
| ***Rhamphoriopsis jiangxiensis*** | **JAUCC7442** | **PZ136834** | **PZ136870** | **PZ136848** | **NA** | **PZ137517** | **This study** |
| *Rhodoveronaea lignicola* | GZCC 23-0624 | PP102212 | PP102209 | PP102216 | PP259405 | PP259401 | Chen et al. (2024) |
| *Rhodoveronaea lignicola* | GZCC 23-0625 | PP102213 | PP102210 | PP102217 | PP259406 | PP259402 | Chen et al. (2024) |
| *Rhodoveronaea nieuwwulvenica* | CBS 149447 | OQ628466 | OQ629048 | NA | OQ627955 | OQ627935 | Crous et al. (2023b) |
| *Rhodoveronaea varioseptata* | CBS 431.88 | EU041813 | EU041870 | NA | NA | NA | Vu et al. (2019) |
| *Rhodoveronaea varioseptata* | CBS 123472 | MG600393 | FJ617559 | MG600408 | NA | JX066701 | Réblová and Štěpánek (2018) |
| *Rhamphoria delicatula* | CBS 132724 | MG600391 | FJ617561 | JX066711 | NA | JX066702 | Vu et al. (2019) |
| *Rhamphoria pyriformis* | CBS 139024 | MG600392 | MG600397 | MG600405 | NA | MG600401 | Réblová et al. (2025b) |
| *Rhamphoria pyriformis* | CBS 139033 | KT991677 | KT991665 | MG600406 | NA | KT991656 | Réblová et al. (2025b) |
| *Xylolentia aseptata* | GZCC 20-0426 | OP377860 | OP377945 | OP378025 | OP473039 | OP473105 | Yang et al. (2023a) |
| *Xylolentia bambusae* | ZHKUCC 24-1142 | PQ376583 | PQ376584 | PQ380133 | PQ383292 | PQ383291 | Zhao et al. (2025) |
| *Xylolentia brunneola* | PRA-13611 | MG600394 | MG600398 | MG600407 | NA | MG600402 | Zhao et al. (2025) |
| *Xylolentia matsushimae* | NN043170 | OL627569 | NA | NA | NA | NA | Wu and Diao (2022) |
| *Xylolentia palmicola* | NN055349 | OL627827 | NA | NA | NA | NA | Wu and Diao (2022) |
| *Xylolentia reniformis* | GZCC 18-0048 | MK547646 | MK547648 | NA | NA | NA | Yuan et al. (2020) |
| *Xylolentia simplex* | BCRCFU31869 | OQ146962 | NA | NA | NA | NA | Kirschner and Hsieh (2023) |
| *Xylolentia simplex* | BCRCFU31758 | OQ146961 | OQ146974 | NA | NA | LC745948 | Kirschner and Hsieh (2023) |
| *Xylolentia hydei* | KUNCC:23-13819 | PQ845758 | PV536235 | PX218386 | PX238324 | PX233777 | Shen et al. (2025) |
| *Xylolentia oblongispora* | DS 1-43 | PQ898745 | PQ898781 | PQ898815 | PV040797 | NA | Lin et al. (2025) |
| ***Xylolentia oblongispora*** | **JAUCC7427** | **PZ136835** | **PZ136871** | **PZ136849** | **PZ150697** | **PZ137518** | **This study** |
| ***Xylolentia oblongispora*** | **JAUCC7428** | **PZ136836** | **PZ136872** | **PZ136850** | **PZ150698** | **PZ137519** | **This study** |
| *Xylolentia subhyalina* | KUNCC 24-17948 | PQ168259 | PQ152651 | PQ218171 | PV443845 | PV443853 | Xu et al. (2025b) |
| *Xylolentia subhyalina* | KUNCC 10481 | PQ168260 | PQ152652 | PQ218172 | PV443844 | PV443851 | Xu et al. (2025b) |
| *Xylolentia yibinensis* | GMBC5367 | PV951777 | PV951771 | PV961435 | PX392374 | PX373392 | Liu et al. (2025) |
| *Xylolentia yibinensis* | GMBC5368 | PV951778 | PV951772 | PV961436 | PX392375 | PX373393 | Liu et al. (2025) |

Newly generated sequences are in red, “NA” indicates the sequence unavailability.

**Supplementary Table 8**. Names, voucher numbers, and corresponding GenBank numbers of the taxa used in the phylogenetic analyses of *Pararoussoella*.

| Species | Voucher number | GenBank accession number | | | | References |
| --- | --- | --- | --- | --- | --- | --- |
|  |  | ITS | LSU | *RPB*2 | *TEF*1-α |  |
| *Aquapteridospora lignicola* | MFLUCC 15–0377 | MZ868774 | KU221018 | MZ892986 | MZ892980 | Yang et al. (2021) |
| *Distoseptispora adscendens* | HKUCC 10820 | NA | DQ408561 | DQ435092 | NA | Zhang et al. (2022) |
| *Distoseptispora amniculi* | MFLUCC 17–2129 | MZ868770 | MZ868761 | MZ892982 | NA | Yang et al. (2021) |
| *Distoseptispora appendiculata* | MFLUCC 18–0259 | MN163009 | MN163023 | NA | MN174866 | Luo et al. (2019) |
| *Distoseptispora aqualignicola* | KUNCC 21–10729 | OK341186 | ON400845 | OP413474 | OP413480 | Zhang et al. (2022) |
| *Distoseptispora aquamyces* | KUNCC 21–10731 | OK341187 | OK341199 | OP413476 | OP413482 | Zhang et al. (2022) |
| *Distoseptispora aquamyces* | UESTCC 24.0165 | PQ067927 | PQ067758 | NA | PQ278579 | Yu et al. (2024b) |
| *Distoseptispora aquatica* | MFLUCC 18–0646 | MK828648 | MK849793 | NA | MN194052 | Yang et al. (2018) |
| *Distoseptispora aquatica* | MFLUCC 15-0374 | MF077552 | KU376268 | NA | NA | Su et al. (2016) |
| *Distoseptispora aquatica* | KUNCC:23-14295 | PQ845847 | PV536295 | PX233766 | PX238244 | Shen et al. (2025) |
| *Distoseptispora aquatica* | KUNCC:23-13801 | PQ845845 | PV536293 | NA | PX238243 | Shen et al. (2025) |
| *Distoseptispora aquatica* | KUNCC:23-13771 | PQ845844 | PV536292 | PX233764 | PX238242 | Shen et al. (2025) |
| *Distoseptispora aquatica* | KUNCC:23-13770 | PQ845843 | PV536291 | PX233763 | PX238241 | Shen et al. (2025) |
| *Distoseptispora aquatica* | KUNCC:23-13769B | PQ845842 | PV536290 | PX233762 | PX238240 | Shen et al. (2025) |
| *Distoseptispora aquatica* | KUNCC:23-13769A | PQ845841 | PV536289 | PX233761 | PX238239 | Shen et al. (2025) |
| *Distoseptispora aquatica=D. nanchangensis* | HJAUP C1074 | OQ942889 | OQ942895 | OQ944460 | OQ944454 | Hu et al. (2023) |
| *Distoseptispora aquatica=D. nanchangensis* | XG431a | PV578166 | PV578332 | PV595318 | PV608838 | Xiong et al. (2026, in press) |
| *Distoseptispora aquatica=D. nanchangensis* | XG431b | PV578167 | PV578333 | PV595319 | PV608839 | Xiong et al. (2026, in press) |
| *Distoseptispora aquatica=D. nanchangensis* | XG412 | PV609768 | NA | PV614734 | PV614738 | Xiong et al. (2026, in press) |
| *Distoseptispora aquatica=D. zhejiangensis* | HJAUP C2588 | PV448668 | PV450539 | NA | PV469765 | Liao et al. (2025) |
| *Distoseptispora aquatica=D. longispora* | HFJAU 0705 | MH555359 | MH555357 | NA | NA | Liao et al. (2025) |
| *Distoseptispora aquisubtropica* | GZCC 22–0075 | ON527933 | ON527941 | ON533685 | ON533677 | Ma et al. (2022) |
| *Distoseptispora arecacearum* | MFLUCC 23–0212 | OR354399 | OR510860 | OR481048 | OR481045 | Karimi et al. (2024) |
| *Distoseptispora atroviridis* | GZCC 20–0511 | MZ868772 | MZ868763 | MZ892984 | MZ892978 | Yang et al. (2021) |
| *Distoseptispora atroviridis* | GZCC 19–0531 | MW133915 | MZ227223 | NA | MZ206155 | Yang et al. (2021) |
| *Distoseptispora bambusae* | MFLUCC 20–0091 | MT232713 | MT232718 | MT232881 | MT232880 | Sun et al. (2020) |
| *Distoseptispora bambusicola* | GZCC 21–0667 | MZ474873 | MZ474872 | NA | OM272845 | Yu et al. (2024b) |
| *Distoseptispora bangkokensis* | MFLUCC 18–0262 | MZ518205 | MZ518206 | NA | OK067246 | Shen et al. (2021) |
| *Distoseptispora cangshanensis* | MFLUCC 16–0970 | MG979754 | MG979761 | NA | MG988419 | Luo et al. (2018a) |
| *Distoseptispora caricis* | CPC 36498 | MN562124 | MN567632 | MN556805 | NA | Crous et al. (2019b) |
| *Distoseptispora changjiangensis* | SAUCC WZS14‐1 | PQ799297 | PQ804723 | PQ849359 | PQ849366 | Liu et al. (2025b) |
| *Distoseptispora changjiangensis* | SAUCC WZS14‐1 | PQ799298 | PQ804724 | PQ849360 | PQ849365 | Liu et al. (2025b) |
| *Distoseptispora chengduensis* | KUNCC:24-17806 | PV918490 | PV918476 | NA | PX048431 | Wang et al. (2025) |
| *Distoseptispora chengduensis* | CGMCC 3.27439 | PQ067913 | PQ067744 | NA | PQ278565 | Yu et al. (2024b) |
| *Distoseptispora chiangraiensis* | MFLU 21-0105 | MZ890145 | MZ890139 | NA | MZ892970 | Manawasinghe et al. (2025) |
| *Distoseptispora chinensis* | GZCC 21–0665 | MZ474871 | MZ474867 | NA | MZ501609 | Hyde et al.(2021b) |
| *Distoseptispora chinensis* | UESTCC 01.0296 | PV383384 | PV368385 | NA | PV752145 | NCBI |
| *Distoseptispora chishuiensis* | GZCC 23‐0729 | PP584670 | PP584767 | NA | PP663310 | Dissanayake et al. (2024) |
| *Distoseptispora clematidis* | MFLUCC 17–2145 | MT310661 | MT214617 | MT394721 | NA | Phukhamsakda et al. (2020) |
| *Distoseptispora clematidis* | HJAUP C1319 | PQ211102 | PQ211110 | PQ303676 | PQ303681 | (Liao et al. 2025a) |
| *Distoseptispora clematidis=D. nabanheensis* | HJAUP C2003 | OP787873 | OP787877 | NA | OP961935 | Liu et al. (2023) |
| *Distoseptispora crassispora* | KUMCC 21–10726 | OK310698 | OK341196 | OP413473 | OP413479 | Zhang et al. (2022) |
| *Distoseptispora crassispora* | ZHKUCC 24-0967 | PQ608227 | PQ608057 | NA | PQ602110 | Lu et al. (2025) |
| *Distoseptispora crassispora* | ZHKUCC 24-0968 | PQ608228 | PQ608058 | NA | PQ602111 | Lu et al. (2025) |
| *Distoseptispora crassispora* | GZCC 23-0606 | OR514699 | OR514708 | OR513897 | OR513900 | Ma et al. (2022) |
| *Distoseptispora curvularia* | KUMCC 21–10725 | OK310697 | OK341195 | OP413472 | OP413478 | Zhang et al. (2022) |
| *Distoseptispora combreticola* | HKAS:132460 | PQ189782 | PQ184737 | NA | NA | Du et al. (2025a) |
| *Distoseptispora cylindricospora* | DLUCC 1906 | OK491122 | OK513523 | NA | OK524220 | Phukhamsakda et al. (2022) |
| *Distoseptispora elongata* | XG345a | PV578164 | PV578330 | PV595316 | PV608836 | Xiong et al. (2026, in press) |
| *Distoseptispora elongata* | XG345b | PV578165 | PV578331 | PV595317 | PV608837 | Xiong et al. (2026, in press) |
| *Distoseptispora daanyuanensis* | SAUCC 12326‐1 | PV670056 | PV670405 | NA | PV708057 | Liu et al. (2025b) |
| *Distoseptispora daanyuanensis* | SAUCC 12326‐2 | PV670057 | PV670406 | NA | PV708058 | Liu et al. (2025b) |
| *Distoseptispora davidalangii* | UESTCC 24.0236 | PQ189781 | PQ184736 | PQ380000 | PQ346519 | Du et al. (2025a) |
| *Distoseptispora davidalangii* | UESTCC 23.0473 | PQ191048 | PQ184723 | PQ379953 | PQ346499 | Du et al. (2025a) |
| *Distoseptispora dehongensis* | KUMCC 18–0090 | MK085061 | MK079662 | NA | MK087659 | Hyde et al. (2019) |
| *Distoseptispora dehongensis* | UESTCC 24.0167 | PQ067921 | PQ067752 | NA | PQ278573 | Yu et al. (2024b) |
| *Distoseptispora dinghuensis* | ZHKUCC:23-0958 | PQ037957 | PQ037956 | PQ035180 | PQ035181 | Dong et al. (2025) |
| ***Distoseptispora dinghuensis*** | **JAUCC 5509** | **OR297670** | **OR336335** | **OR344409** | **OR344394** | **This study** |
| *Distoseptispora dipterocarpi* | MFLUCC 22–0104 | OP600053 | OP600052 | OP595140 | NA | Afshari et al. (2023) |
| *Distoseptispora dujuanhuensis* | KUNCC:23-13772 | PQ845849 | PV536297 | PX233767 | PX238245 | Shen et al. (2025) |
| *Distoseptispora effusa* | GZCC 19–0532 | MW133916 | MZ227224 | NA | MZ206156 | Yang et al. (2021) |
| *Distoseptispora eleiodoxae* | MFLUCC 23–0214 | OR354398 | OR510859 | OR481047 | OR481044 | Karimi et al. (2024) |
| *Distoseptispora euseptata* | MFLUCC 20–0154 | MW081539 | MW081544 | MW151860 | NA | Li et al. (2021) |
| *Distoseptispora euseptata* | DLUCC S2024 | MW081540 | MW081545 | MW084996 | MW084994 | Li et al. (2021) |
| *Distoseptispora fasciculata* | KUMCC 19–0081 | MW286501 | MW287775 | NA | MW396656 | Dong et al. (2021a) |
| *Distoseptispora fluminicola* | DLUCC 0391 | MG979755 | MG979762 | NA | MG988420 | Luo et al. (2018a) |
| *Distoseptispora fluminicola* | DLUCC 0999 | MG979756 | MG979763 | NA | MG988421 | Luo et al. (2018a) |
| *Distoseptispora fluminicola* | MFLUCC 15–0417 | MF077553 | KU376270 | NA | NA | Su et al. (2016) |
| *Distoseptispora fujianensis* | HJAUP C2509 | PQ211095 | PQ211103 | PQ303679 | PQ303682 | Liao et al. (2025a) |
| *Distoseptispora fujianensis* | HJAUP C2513 | PQ211098 | PQ211106 | PQ303680 | PQ303683 | Liao et al. (2025a) |
| *Distoseptispora fusiformis* | GZCC 20–0512 | MZ868773 | MZ868764 | MZ892985 | MZ892979 | Yang et al. (2021) |
| *Distoseptispora gasaensis* | HJAUP C2034 | OQ942896 | OQ942891 | NA | OQ944455 | Hu et al. (2023) |
| *Distoseptispora gelatinosa* | MFLU 24-0292 | PQ570855 | PQ570872 | NA | NA | Sun et al. (2025) |
| *Distoseptispora guanshanensis* | HJAUP C1063 | OQ942894 | OQ942898 | OQ944458 | OQ944452 | Hu et al. (2023) |
| *Distoseptispora guizhouensis* | GZCC 21–0666 | MZ474868 | MZ474869 | MZ501611 | MZ501610 | Hyde et al. (2021) |
| *Distoseptispora guttulata* | MFLU 17–0852 | MF077543 | MF077554 | NA | MF135651 | Yang et al. (2018) |
| *Distoseptispora hainanensis* | GZCC 22‐2047 | OR427328 | OR438894 | OR449119 | OR449122 | Chen et al. (2024b) |
| *Distoseptispora heptapleuricola* | CGMCC 3.27740 | PQ189783 | PQ184738 | PQ380001 | PQ346520 | Du et al. (2025a) |
| *Distoseptispora hongheensis* | KUNCC:23-14299 | PQ845851 | PV536299 | PX233769 | PX238247 | Shen et al. (2025) |
| *Distoseptispora hyalina* | MFLUCC 17–2128 | MZ868769 | MZ868760 | MZ892981 | MZ892976 | Yang et al. (2021) |
| *Distoseptispora hydei* | MFLUCC 20‐0481 | MT734661 | MT742830 | NA | NA | Monkai et al. (2020) |
| *Distoseptispora jianfenglingensis* | SAUCC WZS65‐3 | PQ799299 | PQ804725 | PQ849361 | PQ849367 | Liu et al. (2025b) |
| *Distoseptispora jianfenglingensis* | SAUCC WZS65‐4 | PQ799300 | PQ804726 | PQ849362 | PQ849367 | Liu et al. (2025b) |
| *Distoseptispora jinghongensis* | HJAUP C2120 | OQ942897 | OQ942893 | NA | OQ944456 | Hu et al. (2023) |
| *Distoseptispora jingdongensis* | KUNCC:23-13382 | PQ845852 | PX233770 | PX233770 | PX238248 | Shen et al. (2025) |
| *Distoseptispora lancangjiangensis* | DLUCC 1864 | MW723055 | MW879522 | MW882260 | NA | Shen et al. (2021) |
| *Distoseptispora keviniligustrina* | CGMCC 3.27722 | PQ191052 | PQ184727 | PQ379955 | PQ346501 | Du et al. (2025a) |
| *Distoseptispora lanceolatispora* | GZCC 22‐2045 | OR427329 | OR438895 | OR449120 | OR449123 | Chen et al. (2024b) |
| *Distoseptispora leonensis* | HKUCC 10822 | NA | DQ408566 | DQ435089 | NA | Zhang et al. (2022) |
| *Distoseptispora licualae* | MFLUCC 14–1163A | ON650686 | ON650675 | NA | ON734007 | Konta et al. (2023) |
| *Distoseptispora licualae* | MFLUCC 14–1163B | ON650687 | ON650676 | NA | ON734008 | Konta et al. (2023) |
| *Distoseptispora lignicola* | GZCC 19‐0529 | MW133911 | MZ227219 | NA | MZ206152 | Luo et al. (2019) |
| *Distoseptispora lignicola* | HFJAU 0705 | MK828651 | MK849797 | NA | NA | Luo et al. (2019) |
| *Distoseptispora liupanshuiensis* | GZCC 23‐0730 | PP584669 | PP584766 | NA | PP663309 | Dissanayake et al. (2024) |
| *Distoseptispora longissima* | GMB5340 | PV932969 | PV932988 | PX373357 | PX392331 | Liu et al. (2025a) |
| *Distoseptispora longnanensis* | HJAUP C1040 | OQ942887 | OQ942886 | NA | OQ944451 | Hu et al. (2023) |
| *Distoseptispora martinii* | CGMCC 3.18651 | KU999975 | KX033566 | NA | NA | Xia et al. (2017) |
| *Distoseptispora meilingensis* | JAUCC 4727 | OK562390 | OK562396 | NA | OK562408 | Zhai et al. (2022) |
| *Distoseptispora meilingensis* | JAUCC 4728 | OK562391 | OK562397 | NA | OK562409 | Zhai et al. (2022) |
| *Distoseptispora meilingensis* | UESTCC 24.0169 | PQ067926 | PQ067757 | NA | PQ278578 | Yu et al. (2024b) |
| *Distoseptispora meilingensis* | UESTCC 24.0170 | PQ067928 | PQ067759 | NA | PQ278580 | Yu et al. (2024b) |
| *Distoseptispora menghaiensis* | HJAUP C2045 | OQ942890 | OQ942900 | NA | NA | Hu et al. (2023) |
| *Distoseptispora menglunensis* | HJAUP C2170 | OQ942899 | OQ942888 | OQ944461 | OQ944457 | Hu et al. (2023) |
| *Distoseptispora mengsongensis* | HJAUP C2126 | OP787876 | OP787874 | NA | OP961937 | Liu et al. (2023) |
| *Distoseptispora monospora* | HKAS:145690 | PQ898696 | PQ898699 | PV001672 | PV001669 | Hongsanan et al. (2025) |
| *Distoseptispora motuoensis* | KUNCC24-17628 | PP600327 | PP621731 | NA | NA | Li et al. (2024) |
| *Distoseptispora muchuanensis* | CGMCC 3.27444 | PQ067919 | PQ067750 | NA | PQ278571 | Yu et al. (2024b) |
| *Distoseptispora multiseptata* | MFLUCC 15–0609 | KX710145 | KX710140 | NA | MF135659 | Yang et al. (2018) |
| *Distoseptispora multiseptata* | MFLU 17–0856 | MF077544 | MF077555 | MF135644 | MF135652 | Hyde et al. (2016) |
| *Distoseptispora nanpingensis* | HJAUP C2517 | PQ211096 | PQ211104 | PQ303678 | NA | Liao et al. (2025a) |
| *Distoseptispora narathiwatensis* | MFLUCC 23–0216 | OR354400 | OR510861 | OR481049 | OR481046 | Karimi et al. (2024) |
| *Distoseptispora neorostrata* | MFLUCC 18–0376 | MN163008 | MN163017 | NA | NA | Luo et al. (2019) |
| *Distoseptispora nonrostrata* | KUNCC 21–10730 | OK310699 | OK341198 | OP413475 | OP413481 | Zhang et al. (2022) |
| *Distoseptispora obclavata* | MFLUCC 18–0329 | MN163012 | MN163010 | NA | NA | Luo et al. (2019) |
| *Distoseptispora obpyriformis* | MFLUCC 17–1694 | NA | MG979764 | MG988415 | MG988422 | Luo et al. (2018a) |
| *Distoseptispora obpyriformis* | DLUCC 0867 | MG979757 | MG979765 | MG988416 | MG988423 | Luo et al. (2018a) |
| *Distoseptispora olivaceoviridis* | MFLU 24‐0290 | PQ568144 | PQ569325 | NA | NA | Sun et al. (2025) |
| *Distoseptispora pachyconidia* | KUMCC 21–10724 | OK310696 | OK341194 | OP413471 | OP413477 | Zhang et al. (2022) |
| *Distoseptispora pachyconidia* | KUNCC:23-13048 | PP068489 | PP068866 | PP066112 | NA | Shen et al. (2024) |
| *Distoseptispora palmarum* | MFLUCC 18–1446 | MK085062 | MK079663 | MK087670 | MK087660 | Hyde et al. (2019) |
| *Distoseptispora phangngaensis* | MFLUCC 16–0857 | MF077545 | MF077556 | NA | MF135653 | Yang et al. (2018) |
| *Distoseptispora phangngaensis* | MFLU:17-0855 | MF077545 | MF077556 | NA | NA | Yang et al. (2018) |
| *Distoseptispora phangngaensis=D. thailandica* | MFLUCC 16–0270 | MH275060 | MH260292 | NA | MH412767 | Tibpromma et al. (2018) |
| *Distoseptispora phragmiticola* | GUCC 220201 | OP749887 | OP749880 | OP752699 | OP749891 | Hyde et al. (2023) |
| *Distoseptispora phragmiticola* | GUCC 220202 | OP749888 | OP749881 | OP752700 | OP749892 | Hyde et al. (2023) |
| *Distoseptispora pulchra* | KUNCC:23-14623 | PQ427204 | PQ431179 | PX233824 | PX238335 | Ma et al. (2025) |
| *Distoseptispora quinqueseptata* | GMB5342 | PV932967 | PV932986 | PX373359 | PX392329 | Liu et al. (2025a) |
| ***Distoseptispora quzhouensis*** | **JAUCC5508** | **OR297671** | **OR336336** | **OR344410** | **OR344395** | **This study** |
| ***Distoseptispora quzhouensis*** | **JAUCC5511** | **OR297672** | **OR336337** | **OR344411** | **OR344396** | **This study** |
| *Distoseptispora rayongensis* | MFLUCC 18–0415 | MH457172 | MH457137 | MH463255 | MH463253 | Hyde et al. (2020a) |
| *Distoseptispora rayongensis* | MFLUCC 18–0417 | MH457173 | MH457138 | MH463256 | MH463254 | Hyde et al. (2020a) |
| *Distoseptispora rostrata* | MFLUCC 16–0969 | MG979758 | MG979766 | MG988417 | MG988424 | Luo et al. (2018a) |
| *Distoseptispora rostrata* | DLUCC 0885 | MG979759 | MG979767 | NA | MG988425 | Luo et al. (2018a) |
| *Distoseptispora saprophytica* | MFLUCC 18–1238 | MW286506 | MW287780 | MW504069 | MW396651 | Dong et al. (2021a) |
| *Distoseptispora septata* | GZCC 22–0078 | ON527939 | ON527947 | ON533690 | ON533683 | Ma et al. (2022) |
| *Distoseptispora songkhlaensis* | MFLUCC 18–1234 | MW286482 | MW287755 | NA | MW396642 | Dong et al. (2021a) |
| *Distoseptispora suae* | CGMCC 3.24262 | OQ874968 | OQ732679 | OQ870341 | OR367670 | Shen et al. (2024) |
| *Distoseptispora subtropica* | HJAUP C2528 | PQ211099 | PQ211107 | PQ303677 | PQ303684 | Liao et al. (2025a) |
| *Distoseptispora subtropica* | HJAUP C2535 | PQ211097 | PQ211105 | NA | PQ303685 | Liao et al. (2025a) |
| *Distoseptispora suoluoensis* | MFLUCC 17–0224 | MF077546 | MF077557 | NA | MF135654 | Yang et al. (2018) |
| *Distoseptispora suoluoensis* | MFLUCC 17–0854 | MF077547 | MF077558 | MZ945510 | NA | Yang et al. (2018) |
| *Distoseptispora suoluoensis* | UESTCC 24.0172 | PQ067916 | PQ067747 | PQ186979 | PQ278568 | Yu et al. (2024b) |
| *Distoseptispora tectonae* | MFLUCC 12–0291 | KX751711 | KX751713 | KX751708 | KX751710 | Hyde et al. (2016) |
| *Distoseptispora tectonae* | MFLU 20–0262 | MT232714 | MT232719 | NA | NA | Sun et al. (2020) |
| *Distoseptispora tectonae=D. ganzhouensis* | HJAUP C1090 | PQ211100 | PQ211108 | NA | PQ303687 | Liao et al. (2025a) |
| *Distoseptispora tectonae=D. sinensis* | HJAUP C2044 | OP787878 | OP787875 | NA | OP961936 | Liu et al. (2023) |
| *Distoseptispora tectonae=D. submersa* | MFLUCC 16‐0946 | MG979760 | MG979768 | MG988418 | MG988426 | Luo et al. (2018a) |
| *Distoseptispora tectonae=D. bawanglingensis* | SAUCC WZS13‐1 | PQ799295 | PQ804721 | PQ849357 | PQ849363 | Liu et al. (2025b) |
| *Distoseptispora tectonae=D. bawanglingensis* | SAUCC WZS13‐2 | PQ799296 | PQ804722 | PQ849358 | PQ849364 | Liao et al. (2025b) |
| *Distoseptispora tectonae=D. yichunensis* | HJAUP C1065 | OQ942885 | OQ942892 | OQ944459 | OQ944453 | Hu et al. (2023) |
| *Distoseptispora tectonae=D. yichunensis* | UESTCC:23.0472 | PQ191057 | PQ184732 | NA | NA | Du et al. (2025b) |
| *Distoseptispora tectonae=D. yichunensis* | XG347a | PV578170 | PV578336 | PV595322 | PV608842 | Xiong et al. (2026, in press) |
| *Distoseptispora tectonae=D. yichunensis* | XG347b | PV578171 | PV578337 | PV595323 | PV608843 | Xiong et al. (2026, in press) |
| *Distoseptispora tectonae=D. sichuanensis* | KUNCC 23-15518 | PP584671 | PP584768 | NA | PP663311 | Dissanayake et al. (2024) |
| *Distoseptispora tectonae=D. sichuanensis* | KUNCC 23‐15519 | PP584672 | PP584769 | NA | PP663312 | Dissanayake et al. (2024) |
| *Distoseptispora tectonigena* | MFLUCC 12–0292 | KX751712 | KX751714 | KX751709 | NA | Hyde et al. (2016) |
| *Distoseptispora tectonigena* | MFLU 24-0291 | PQ568145 | PQ569326 | NA | NA | Sun et al. (2024) |
| *Distoseptispora terrestris* | HJAUP C2539 | PV448667 | PV450538 | NA | PV469764 | Liao et al. (2025b) |
| *Distoseptispora thysanolaenae* | KUN–HKAS 112710 | MW723057 | MW879524 | NA | MW729783 | Shen et al. (2021) |
| *Distoseptispora thysanolaenae* | KUN–HKAS 102247 | MK045851 | MK064091 | NA | MK086031 | Phookamsak et al. (2019) |
| *Distoseptispora tongrensis* | GMB5344 | PV932971 | PV932990 | PX373361 | NA | Liu et al. (2025a) |
| *Distoseptispora tropica* | GZCC 22–0076 | ON527935 | ON527943 | ON533687 | ON533679 | Ma et al. (2022) |
| *Distoseptispora uncariicola* | UESTCC 24.0229 | PQ189784 | PQ184739 | PQ380002 | PQ346521 | Du et al. (2025a) |
| *Distoseptispora verrucosa* | GZCC 20–0434 | MZ868771 | MZ868762 | MZ892983 | MZ892977 | Yang et al. (2021) |
| *Distoseptispora wuzhishanensis* | GZCC 22–0077 | ON527938 | ON527946 | NA | ON533682 | Ma et al. (2022) |
| *Distoseptispora wuyishanensis* | HJAUP C2515 | PV448666 | PV450537 | PV469759 | PV469763 | Liao et al. (2025b) |
| *Distoseptispora xinpingensis* | KUNCC 22–12667 | OQ874970 | OQ732681 | OQ870340 | OR367671 | Shen et al. (2024) |
| *Distoseptispora xishuangbannaensis* | KUMCC 17–0290 | MH275061 | MH260293 | MH412754 | MH412768 | Tibpromma et al. (2018) |
| *Distoseptispora yongxiuensis* | JAUCC 4725 | OK562388 | OK562394 | NA | OK562406 | Zhai et al. (2022) |
| *Distoseptispora yongxiuensis* | UESTCC 24.0176 | PQ067922 | PQ067753 | NA | PQ278574 | Yu et al. (2024b) |
| *Distoseptispora yongxiuensis* | UESTCC 24.0177 | PQ067923 | PQ067754 | NA | PQ278575 | Yu et al. (2024b) |
| *Distoseptispora yongxiuensis* | UESTCC 24.0178 | PQ067924 | PQ067755 | NA | PQ278576 | Yu et al. (2024b) |
| *Distoseptispora yongxiuensis* | UESTCC 24.0179 | PQ067925 | PQ067756 | NA | PQ278577 | Yu et al. (2024b) |
| *Distoseptispora yunjushanensis* | JAUCC 4724 | OK562392 | OK562398 | NA | OK562410 | Zhai et al. (2022) |
| *Distoseptispora yunjushanensis* | HJAUP C1307 | PQ211101 | PQ211109 | PQ303675 | PQ303686 | Liao et al. (2025a) |
| *Distoseptispora yunnanensis* | MFLUCC 20–0153 | MW081541 | MW081546 | MW151861 | MW084995 | Li et al. (2021) |
| *Distoseptispora zunyiensis* | KUNCC:24-18628 | PV918496 | PV918482 | NA | PX048436 | Dong et al. (2025) |

Newly generated sequences are in red, “NA” indicates the sequence unavailability.

**References**

Ariyawansa HA, Hyde KD, Jayasiri SC, et al. (2015) Fungal diversity notes 111–252 – taxonomic and phylogenetic contributions to fungal taxa. Fungal Diversity 75: 27–274.

Al-Bedak OA, Ismail MA, Mohamed RA (2019) *Paracremonium moubasheri*, a new species from an alkaline sediment of Lake Hamra in Wadi-El-Natron, Egypt with a key to the accepted species. Studies in Fungi 4: 216–222.

Afshari N, Gomes De Farias A, Bhunjun C, et al. (2023) *Distoseptispora dipterocarpi* sp. nov. (*Distoseptisporaceae*), a lignicolous fungus on decaying wood of *Dipterocarpus* in Thailand. CREAM 13: 68–78. https://doi.org/10.5943/cream/13/1/5

Arzanlou M, Groenewald JZ, Gams W, et al (2007) Phylogenetic and morphotaxonomic revision of *Ramichloridium* and allied genera. Studies in Mycology 58: 57–93. https://doi.org/10.3114/sim.2007.58.03

Boonmee S, D’souza MJ, Luo Z, et al. (2016) *Dictyosporiaceae* fam. nov. Fungal Diversity 80: 457–482.

Bao DF, Tian XG, Samarakoon MC, et al. (2025) Biodiversity of lignicolous freshwater fungi from the Nanpan River Basin in Guizhou and Guangxi Provinces, China, with descriptions of fifteen species. Mycosphere 16 (1): 3695–3752.

Bao DF, Hyde KD, McKenzie EH, et al. (2021) Biodiversity of lignicolous freshwater hyphomycetes from China and Thailand and description of sixteen species. Journal of Fungi 7(8): 669.

Bao DF, Bhat DJ, Boonmee S, et al (2022) Lignicolous freshwater ascomycetes from Thailand: Introducing *Dematipyriforma muriformis* sp. nov., one new combination and two new records in *Pleurotheciaceae*. MC 93: 57–79. https://doi.org/10.3897/mycokeys.93.87797

Bao SX, Xu RJ, Zhu YA, et al. (2023) *Rhexoacrodictys melanospora* sp. nov. (*Rhexoacrodictys*, *Pleurotheciales*) from Yunnan, China. Phytotaxa 594: 213–222. https://doi.org/10.11646/phytotaxa.594.3.5

Boonmee S, Wanasinghe DN, Calabon MS, et al (2021) Fungal diversity notes 1387–1511: taxonomic and phylogenetic contributions on genera and species of fungal taxa. Fungal Diversity 111: 1–335. https://doi.org/10.1007/s13225-021-00489-3

Boonyuen N, Chuaseeharonnachai C, Suetrong S, et al (2011) *Savoryellales* (*Hypocreomycetidae*, *Sordariomycetes*): a novel lineage of aquatic ascomycetes inferred from multiple-gene phylogenies of the genera *Ascotaiwania, Ascothailandia* , and *Savoryella*. Mycologia 103: 1351–1371. https://doi.org/10.3852/11-102

Chen XM, Tang X, Du TY, et al. (2024a) Two new species of *Rhodoveronaea* (*Rhamphoriales*, Ascomycota) from terrestrial habitats in China. Phytotaxa 638: 49–60. https://doi.org/10.11646/phytotaxa.638.1.4

Chen XM, Tang X, Ma J, et al (2024b) Identification of two new species and a new host record of *Distoseptispora* (*Distoseptisporaceae*, *Distoseptisporales*, *Sordariomycetes*) from terrestrial and freshwater habitats in Southern China. MC 102: 83–105. https://doi.org/10.3897/mycokeys.102.115452

Crous PW, Shivas RG, Quaedvlieg WV, et al. (2014) Fungal Planet description sheets: 214–280. Persoonia-Molecular Phylogeny and Evolution of Fungi 30: 184-306.

Crous PW, Wingfield MJ, Richardson DM, et al. (2016) Fungal Planet description sheets: 400–468. Persoonia-Molecular Phylogeny and Evolution of Fungi 36(1): 316–458.

Crous PW, Wingfield MJ, Burgess TI, et al. (2017) Fungal Planet description sheets: 625–715. Persoonia: Molecular Phylogeny and Evolution of Fungi 39: 270.

Crous PW, Schumacher RK, Wingfield MJ, et al (2018) New and Interesting Fungi. 1. Fungal Systematics and Evolution 1: 169–215. https://doi.org/10.3114/fuse.2018.01.08

Crous PW, Carnegie AJ, Wingfield MJ, et al. (2019a) Fungal Planet description sheets: 868–950. Persoonia-Molecular Phylogeny and Evolution of Fungi 42: 291-473.

Crous PW, Wingfield MJ, Lombard L, et al (2019b) Fungal Planet description sheets: 951–1041. persoonia 43: 223–425. https://doi.org/10.3767/persoonia.2019.43.06

Crous PW, Wingfield MJ, Schumacher RK, et al. (2020). New and interesting fungi. 3. Fungal Systematics and Evolution 6: 157.

Crous PW, Osieck ER, Jurjević Ž, (2021a) Fungal Planet description sheets: 1284–1382. Persoonia: Molecular Phylogeny and Evolution of Fungi 47: 178.

Crous PW, Hernández-Restrepo M, Schumacher RK, et al. (2021b) New and interesting fungi. 4. Fungal Systematics and Evolution 7: 255.

Crous PW, Boers J, Holdom D, et al. (2022) Fungal Planet description sheets: 1383–1435. Persoonia-Molecular Phylogeny and Evolution of Fungi 48(1): 261–371.

Crous PW, Akulov A, Balashov S, et al. (2023a) New and Interesting Fungi. 6. Fungal Systematics and Evolution 11: 109–156

Crous PW, Osieck ER, Shivas RG, et al (2023b) Fungal Planet description sheets: 1478–1549. persoonia 50: 158–310. https://doi.org/10.3767/persoonia.2023.50.05

Crous PW, Catcheside DE, Catcheside PS et al. (2025) Fungal Planet description sheets: 1781–1866. Persoonia: Molecular Phylogeny and Evolution of Fungi 54: 327.

Du H, Chi M, Wu N, et al (2025a) Taxonomic and phylogenetic insights to *Dothideomycetes* and *Sordariomycetes* associated with medicinal plants in Southwestern China. mycosphere 16:179–343. https://doi.org/10.5943/mycosphere/16/2/2

Du H-Z, Liu N-G, Wu N, et al (2025b) Morpho-phylogenetic evidence reveals novel hyphomycetous fungi on medicinal plants in Southwestern China. Mycology 16: 1023–1082. https://doi.org/10.1080/21501203.2024.2444436

Dong W, Hyde KD, Jeewon, et al. 2023. Mycosphere notes 449–468: saprobic and endophytic fungi in China, Thailand, and Uzbekistan. Mycosphere 14(1): 2208-2262.

Dissanayake LS, Samarakoon MC, Maharachchikumbura SS, et al (2024) Exploring the taxonomy and phylogeny of *Sordariomycetes* taxa emphasizing *Xylariomycetidae* in Southwestern China. Mycosphere 15: 1675–1793. https://doi.org/10.5943/mycosphere/15/1/15

Dong W, Hyde K, Jeewon R, et al (2021a) Towards a natural classification of annulatascaceae-like taxa Ⅱ: introducing five new genera and eighteen new species from freshwater. Mycosphere 12:1–88. <https://doi.org/10.5943/mycosphere/12/1/1>

Dong W, Jeewon R, Hyde KD, et al (2021b) Five Novel Taxa from Freshwater Habitats and New Taxonomic Insights of *Pleurotheciales* and *Savoryellomycetidae*. JoF 7:711. https://doi.org/10.3390/jof7090711

Dong W, Hyde KD, Jeewon R, et al (2025) Fungal diversity notes 2017–2122: taxonomic and phylogenetic contributions to freshwater fungi and other fungal taxa. Fungal Diversity 134:185–459. https://doi.org/10.1007/s13225-025-00560-3

Ding MY, Chen W, Ma XC, et al. (2021) Emerging salt marshes as a source of *Trichoderma arenarium* sp. nov. and other fungal bio effectors for bio saline agriculture. Journal of applied microbiology 130(1): 179–195.

Fernández FA, Lutzoni FM and Huhndorf SM. (1999) Teleomorph-anamorph connections: the new pyrenomycetous genus *Carpoligna* and its *Pleurothecium* anamorph. Mycologia 91(2): 251–262.

Ferreira BW, Barreto RW. (2019) Debunking *Acroconidiella*. Mycological Progress. 18(11): 1303–1315

Fryar SC, Catcheside DEA (2023) Freshwater ascomycetes from southern Australia *: Melanascomaceae* fam. nov., *Melanascoma panespora* gen. et. sp. nov., and *Pleurothecium* *brunius* sp. nov. Fungal Systematics and Evolution 11: 85–93. https://doi.org/10.3114/fuse.2023.11.07

Habib K, Wh L, Yl R, et al (2025) Exploration of ascomycetous fungi revealing novel taxa in Southwestern China. mycosphere 16: 1412–1529. https://doi.org/10.5943/mycosphere/16/1/9

He W-M, Zhang J-B, Zhai Z-J, et al (2024) Four novel species of *Pleurotheciaceae* collected from freshwater habitats in Jiangxi Province, China. Front Microbiol 15: 1452499. https://doi.org/10.3389/fmicb.2024.1452499

Hernández-Restrepo M, Gené J, Castañeda-Ruiz RF, et al (2017) Phylogeny of saprobic microfungi from Southern Europe. Studies in Mycology 86: 53–97. https://doi.org/10.1016/j.simyco.2017.05.002

Hongsanan S, Khuna S, Manawasinghe I, et al (2025) Mycosphere Notes 521–571: A special edition of fungal biodiversity to celebrate Kevin D. Hyde’s 70th birthday and his exceptional contributions to Mycology. mycosphere 16:1–178. https://doi.org/10.5943/mycosphere/16/2/1

Hu Y-F, Liu J-W, Luo X-X, et al (2023) Multi-locus phylogenetic analyses reveal eight novel species of *Distoseptispora* from southern China. Microbiol Spectr 11: e02468–23. https://doi.org/10.1128/spectrum.02468-23

Huang S-P, Bao D-F, Shen H-W, et al (2022) *Neomonodictys aquatica* sp. nov. (*Pleurotheciaceae*) from a plateau lake in Yunnan Province, China. BDJ 10: e76842. https://doi.org/10.3897/BDJ.10.e76842

Hernández-Restrepo M, Giraldo A, Van Doorn R, et al. (2020) The Genera of Fungi–G6: *Arthrographis, Kramasamuha, Melnikomyces, Thysanorea*, and *Verruconis*. Fungal Systematics and Evolution 6(1): 1–24.

Huhndorf SM, Miller AN, Fernández FA. (2004) Molecular systematics of the *Sordariales*: the order and the family *Lasiosphaeriaceae* redefined. Mycologia, 96(2): 368–387.

Hyde KD, Norphanphoun C, Ma J, et al. (2023) Mycosphere notes 387–412 – novel species of fungal taxa from around the world. Mycosphere 14: 663–744. https://doi.org/10.5943/mycosphere/14/1/8

Hyde KD, Norphanphoun C, Maharachchikumbura S, et al. (2020a) Refined families of *Sordariomycetes*. Mycosphere 11: 305–1059. https://doi.org/10.5943/mycosphere/11/1/7

Hyde KD, Dong Y, Phookamsak R, et al. (2020b) Fungal diversity notes 1151–1276: taxonomic and phylogenetic contributions on genera and species of fungal taxa. Fungal Diversity 100: 5–277. https://doi.org/10.1007/s13225-020-00439-5

Hyde KD, Hongsanan S, Jeewon R, et al. (2016) Fungal diversity notes 367–490: taxonomic and phylogenetic contributions to fungal taxa. Fungal Diversity 80: 1–270. https://doi.org/10.1007/s13225-016-0373-x

Hyde KD, Norphanphoun C, Abreu VP, et al. (2017) Fungal diversity notes 603–708: taxonomic and phylogenetic notes on genera and species. Fungal Diversity 87: 1–235. https://doi.org/10.1007/s13225-017-0391-3

Hyde KD, Tennakoon DS, Jeewon R, et al. (2019) Fungal diversity notes 1036–1150: taxonomic and phylogenetic contributions on genera and species of fungal taxa. Fungal Diversity 96: 1–242. https://doi.org/10.1007/s13225-019-00429-2

Hyde KD, Chaiwan N, Norphanphoun C, et al. (2018) Mycosphere notes 169–224. Mycosphere 9(2): 271–430

Hyde KD, Bao DF, Hongsanan S, et al. (2021a) Evolution of freshwater *Diaporthomycetidae* (*Sordariomycetes*) provides evidence for five new orders and six new families. Fungal Diversity 107: 71–105

Hyde KD, Suwannarach N, Jayawardena RS, et al. (2021b) Mycosphere notes 325-344 – Novel species and records of fungal taxa from around the world. Mycosphere 12(1): 1101–1156

Iturrieta-Gonzalez I, Gené J, Guarro J, et al. (2018) *Neodendryphiella*, a novel genus in the family *Dictyosporiaceae*. MycoKeys 37: 19–38

Jayawardena RS, Hyde KD, Wang S, et al (2022) Fungal diversity notes 1512–1610: taxonomic and phylogenetic contributions on genera and species of fungal taxa. Fungal Diversity 117: 1–272. https://doi.org/10.1007/s13225-022-00513-0

Kirschner R, Pang KL, Jones EG. (2013) Two cheirosporous hyphomycetes reassessed based on morphological and molecular examination. Mycological Progress 12(1): 29–36.

Khemmuk W, Geering AD, Shivas RG. (2016) *Wongia* gen. nov. (*Papulosaceae, Sordariomycetes*), a new generic name for two root-infecting fungi from Australia. IMA fungus 7(2): 247–252.

Kuo CH, Hsieh SY, Goh TK. (2024) A new species of *Wongia* (*Papulosaceae, Sordariomycetes*) from Taiwan. Phytotaxa 662(2): 177–186.

Kirschner R, Hsieh SY. (2023) *Xylolentia* *simplex*, a hyphomycete from dead petioles of the fern *Angiopteris lygodiifolia* in Taiwan. Fungal Science 38: 13–21.

Karimi O, Chethana KWT, De Farias ARG, et al (2024) Morphology and multigene phylogeny reveal three new species of *Distoseptispora* (*Distoseptisporales*, *Distoseptisporaceae*) on palms (*Arecaceae*) from peatswamp areas in southern Thailand. MC 102: 55–81. https://doi.org/10.3897/mycokeys.102.112815

Konta S, Tibpromma S, Karunarathna S, et al (2023) Morphology and multigene phylogeny reveal ten novel taxa in Ascomycota from terrestrial palm substrates (*Arecaceae*) in Thailand. Mycosphere 14: 107–152. https://doi.org/10.5943/mycosphere/14/1/2

Li XH, Liu YL, Song HY, et al. (2021) *Sporidesmiella lignicola* sp. nov., a new hyphomycetous fungus from freshwater habitats in China. Biodiversity Data Journal 9(e77414): 1–14.

Li J-N, Xu R-J, Xu K, et al. (2024) *Distoseptispora motuoensis* (*Distoseptisporaceae*), a new freshwater hyphomycetous species from Xizang Autonomous Prefecture, China. Phytotaxa 675: 122–134. https://doi.org/10.11646/phytotaxa.675.2.3

Li W-L, Liu Z-P, Zhang T, et al. (2021) Additions to *Distoseptispora* (*Distoseptisporaceae*) associated with submerged decaying wood in China. Phytotaxa 520: 75–86. https://doi.org/10.11646/phytotaxa.520.1.5

Liao M-G, Luo X-X, Hu Y-F, et al. (2025a) Morphological and phylogenetic analyses reveal four novel species of *Distoseptispora* (*Distoseptisporaceae, Distoseptisporales*) from southern China. MC 113: 31–55. https://doi.org/10.3897/mycokeys.113.137082

Liao M-G, Luo X-X, Xia J-W, et al. (2025b) Six Novel Species of *Distoseptispora* (*Distoseptisporaceae, Distoseptisporales*) and *Helminthosporium* (*Massarinaceae, Pleosporales*) Isolated from Terrestrial Habitats in Southern China. JoF 11: 494. https://doi.org/10.3390/jof11070494

Lin C-G, Hyde KD, Feng Y, et al. (2025) Notes, outline, systematics and phylogeny of hyaline-spored hyphomycetes. Fungal Diversity 135: 57–467. https://doi.org/10.1007/s13225-025-00561-2

Lin C-G, Liu J-K, Chukeatirote E, Hyde KD, et al. (2023) *Rhamphoriopsis hyalospora* sp. nov. associated with decaying wood from China. Phytotaxa 598: 245–253. https://doi.org/10.11646/phytotaxa.598.3.6

Liu J, Hu Y, Luo X, et al (2023) Morphological and Phylogenetic Analyses Reveal Three New Species of *Distoseptispora* (*Distoseptisporaceae, Distoseptisporales*) from Yunnan, China. JoF 9: 470. https://doi.org/10.3390/jof9040470

Liu L, Zhang Q, Li W, et al (2025a) Diversity and taxonomy of hyphomycetous Fungi in Southwestern China. mycosphere 16: 3951–4062. https://doi.org/10.5943/mycosphere/16/1/31

Liu W, Yin C, Jiang Y, et al (2025b) Discovery and Identification of Four Novel Species of *Distoseptispora* (*Distoseptisporaceae, Distoseptisporales*) on Decaying Wood from Hainan and Fujian Provinces, China. JoF 11: 667. https://doi.org/10.3390/jof11090667

Lu L, Karunarathna SC, Xiong Y-R, et al. (2025) Taxonomy and systematics of micro-fungi associated with Coffea in southern China and northern Thailand. Fungal Diversity, 135(1): 469–743.

Luo Z, Hyde K, Liu J, et al. (2018a) Lignicolous freshwater fungi from China II: Novel *Distoseptispora* (*Distoseptisporaceae*) species from northwestern Yunnan Province and a suggested unified method for studying lignicolous freshwater fungi. Mycosphere 9: 444–461. https://doi.org/10.5943/mycosphere/9/3/2

Luo Z-L, Hyde KD, Bhat DJ, et al. (2018b) Morphological and molecular taxonomy of novel species *Pleurotheciaceae* from freshwater habitats in Yunnan, China. Mycol Progress 17: 511–530. https://doi.org/10.1007/s11557-018-1377-6

Liu NG, Hongsanan S, Yang J, et al. (2017) *Dendryphiella fasciculata* sp. nov. and notes on other *Dendryphiella* species. Mycosphere 8(9): 1575–86.

Luo ZL, Hyde KD, Liu JK, et al. (2019) Freshwater sordariomycetes. Fungal diversity, 99(1): 451–660.

Liu NG, Hyde KD, Sun YR, et al. (2024a) Notes, outline, taxonomy and phylogeny of brown-spored hyphomycetes. Fungal Diversity 129(1): 1–281.

Liu SL, Wang XW, Li GJ, et al. (2024b) Fungal diversity notes 1717–1817: taxonomic and phylogenetic contributions on genera and species of fungal taxa. Fungal Diversity 124: 1–216

Lombard L, Van der Merwe NA, Groenewald JZ, et al. (2015) Generic concepts in Nectriaceae. Studies in Mycology 80(1): 189–245.

Lynch SC, Twizeyimana M, Mayorquin JS, et al. (2016) Identification, pathogenicity and abundance of *Paracremonium pembeum* sp. nov. and *Graphium euwallaceae* sp. nov.—two newly discovered mycangial associates of the polyphagous shot hole borer (*Euwallacea* sp.) in California. Mycologia 108(2): 313–329.

Miller AN, Huhndorf SM, Fournier J (2014) Phylogenetic relationships of five uncommon species of *Lasiosphaeria* and three new species in the *Helminthosphaeriaceae* (*Sordariomycetes*). Mycologia 106:505–524.

Miller AN, Huhndorf SM (2004) A natural classification of *Lasiosphaeria* based on nuclear LSU rDNA sequences. Mycological Research 108:26–34.

Ma YR (2016) Morphological and molecular systematics classification of *Dysporum tarda* fungi in five southern provinces of China (Master's thesis, Shandong Agricultural University).

Ma J, Zhang JY, Xiao XJ, et al. (2022) Multi-Gene Phylogenetic Analyses Revealed Five New Species and Two New Records of *Distoseptisporales* from China. Journal of Fungi 8:1202. <https://doi.org/10.3390/jof8111202>

Ma Q, Wang Y, Shen HW, et al. (2025) *Distoseptispora pulchra* sp. nov. and *D. liupanshuiensis* from freshwater habitats in Yunnan Province, China. Phytotaxa 725:24–40. <https://doi.org/10.11646/phytotaxa.725.1.3>

Monkai J, Boonmee S, Ren GC, et al. (2020) *Distoseptispora hydei* sp. nov. (*Distoseptisporaceae*), a novel lignicolous fungus on decaying bamboo in Thailand. Phytotaxa 459: 93–107.  <https://doi.org/10.11646/phytotaxa.459.2.1>

Manawasinghe IS, Hyde KD, Wanasinghe DN, et al. (2025) Fungal diversity notes 1818–1918: taxonomic and phylogenetic contributions on genera and species of fungi. Fungal Diversity 130:1–261.

Phookamsak R, Hyde KD, Jeewon R, et al. (2019) Fungal diversity notes 929–1035: taxonomic and phylogenetic contributions on genera and species of fungi. Fungal Diversity 95:1–273. <https://doi.org/10.1007/s13225-019-00421-w>

Phukhamsakda C, McKenzie EHC, Phillips AJL, et al. (2020) Microfungi associated with *Clematis* (*Ranunculaceae*) with an integrated approach to delimiting species boundaries. Fungal Diversity 102:1–203. <https://doi.org/10.1007/s13225-020-00448-4>

Phukhamsakda C, Nilsson RH, Bhunjun CS, et al. (2022) The numbers of fungi: contributions from traditional taxonomic studies and challenges of metabarcoding. Fungal Diversity 114:327–386. <https://doi.org/10.1007/s13225-022-00502-3>

Réblová M, Kolařík M, Nekvindová J, et al. (2021) Phylogeny, global biogeography and pleomorphism of *Zanclospora*. Microorganisms 9:706.

Réblová M (2013) Two taxonomic novelties in the *Sordariomycetidae*: *Ceratolenta caudata* gen. et sp. nov. and *Platytrachelon abietis* gen. et comb. nov. for *Ceratosphaeria abietis*. Mycologia 105:462–475.

Ranghoo VM, Hyde KD, Wong SW, et al. (2000) *Vertexicola caudatus* gen. et sp. nov., and a new species of *Rivulicola* from submerged wood in freshwater habitats. Mycologia 92:1019–1026.

Réblová M, Nekvindová J, Bauchová L, et al. (2025a) *Pleurophragmium parvisporum* (Ascomycota): One name, seven stories–a case highlighting the need for verification of strains from public culture collections. IMA Fungus 16:e173033.

Réblová M, Hernández-Restrepo M, Fournier J, et al. (2020) New insights into the systematics of *Bactrodesmium* and its allies and introducing new genera, species and morphological patterns in the *Pleurotheciales* and *Savoryellales* (*Sordariomycetes*). Studies in Mycology 95:415–466. <https://doi.org/10.1016/j.simyco.2020.02.002>

Réblová M, Nekvindová J, Hernández-Restrepo M, et al. (2025b) Phylogeny, taxonomy and geographic distribution of novel and known fungi with holoblastic-denticulate conidiogenesis in *Rhamphoriales* and *Pleurotheciales* (*Sordariomycetes*). Persoonia 55:277–312. <https://doi.org/10.3114/persoonia.2025.55.08>

Réblová M, Seifert KA, Fournier J, et al. (2016) Newly recognized lineages of perithecial ascomycetes: the new orders *Conioscyphales* and *Pleurotheciales*. Persoonia 37:57–81. <https://doi.org/10.3767/003158516X689819>

Réblová M, Seifert KA, Fournier J, et al. (2012) Phylogenetic classification of *Pleurothecium* and *Pleurotheciella* gen. nov. and its dactylaria-like anamorph (*Sordariomycetes*) based on nuclear ribosomal and protein-coding genes. Mycologia 104:1299–1314. <https://doi.org/10.3852/12-035>

Réblová M, Štěpánek V (2018) Introducing the *Rhamphoriaceae*, fam. nov. (*Sordariomycetes*), two new genera, and new life histories for taxa with phaeoisaria - and idriella -like anamorphs. Mycologia 110:750–770. <https://doi.org/10.1080/00275514.2018.1475164>

Song HY, Huo GH, Hu DM (2018) *Dictyosporella hydei* sp. nov., an asexual species from freshwater habitats in China. Phytotaxa 358:181–188.

Shen HW, Bao DF, Luan S, et al. (2025) Taxonomy and phylogeny of lignicolous freshwater fungi from plateau lakes in Yunnan Province, China. Fungal Diversity 134:635–899.

Shen HW, Bao DF, Boonmee S, et al. (2024) Diversity of *Distoseptispora* (*Distoseptisporaceae*) taxa on submerged decaying wood from the Red River in Yunnan, China. MycoKeys 102:1–28. <https://doi.org/10.3897/mycokeys.102.116096>

Shen HW, Bao DF, Hyde KD, et al. (2021) Two novel species and two new records of *Distoseptispora* from freshwater habitats in China and Thailand. MycoKeys 84:79–101. <https://doi.org/10.3897/mycokeys.84.71905>

Spatafora JW, Sung GH, Johnson D, et al. (2006) A five-gene phylogeny of *Pezizomycotina*. Mycologia 98:1018–1028.

Schroers HJ, Geldenhuis MM, Wingfield MJ, et al. (2005) Classification of the guava wilt fungus *Myxosporium psidii*, the palm pathogen *Gliocladium vermoesenii* and the persimmon wilt fungus *Acremonium diospyri* in Nalanthamala. Mycologia 97:375–395.

Shi L, Yang H, Hyde KD, et al. (2021) Freshwater *Sordariomycetes*: new species and new records in *Pleurotheciaceae, Pleurotheciales*. Phytotaxa 518:143–166. <https://doi.org/10.11646/phytotaxa.518.2.4>

Sri-indrasutdhi V, Boonyuen N, Suetrong S, et al. (2010) Wood-inhabiting freshwater fungi from Thailand: *Ascothailandia grenadoidia* gen. et sp. nov., *Canalisporium grenadoidia* sp. nov. with a key to *Canalisporium* species (*Sordariomycetes*, Ascomycota). Mycoscience 51:411–420. <https://doi.org/10.1007/S10267-010-0055-6>

Su H, Hyde KD, Maharachchikumbura SSN, et al. (2016) The families *Distoseptisporaceae* fam. nov., *Kirschsteiniotheliaceae*, *Sporormiaceae* and *Torulaceae*, with new species from freshwater in Yunnan Province, China. Fungal Diversity 80:375–409. <https://doi.org/10.1007/s13225-016-0362-0>

Sun LY, Li HY, Sun X, et al. (2017) *Dematipyriforma aquilaria* gen. et sp. nov., a New Hyphomycetous Taxon from *Aquilaria crassna*. Cryptogamie, Mycologie 38:341–351. <https://doi.org/10.7872/crym/v38.iss3.2017.341>

Sun Y, Goonasekara I, Thambugala K, et al. (2020) *Distoseptispora bambusae* sp. nov. (*Distoseptisporaceae*) on bamboo from China and Thailand. Biodiversity Data Journal 8:e53678. <https://doi.org/10.3897/BDJ.8.e53678>

Sun YR, Hyde KD, Liu NG, et al. (2024) Micro-fungi on medicinal plants in southern China and northern Thailand.

Sun YR, Hyde KD, Liu NG, et al. (2025) Micro-fungi in southern China and northern Thailand: emphasis on medicinal plants. Fungal Diversity 131:99–299. <https://doi.org/10.1007/s13225-024-00549-4>

Tanaka K, Hirayama K, Yonezawa H, et al. (2015) Revision of the *Massarineae* (*Pleosporales, Dothideomycetes*). Studies in Mycology 82:75–136.

Tan YP, Bishop-Hurley SL, Shivas RG, et al. (2022) Fungal Planet description sheets: 1436–1477. Persoonia 49:261–350.

Tian WH, Jin Y, Liao YC, et al. (2024) New and Interesting Pine-Associated Hyphomycetes from China. Journal of Fungi 10(8, no. 546):1–20.

Tibpromma S, Hyde KD, McKenzie EHC, et al. (2018) Fungal diversity notes 840–928: micro-fungi associated with *Pandanaceae*. Fungal Diversity 93:1–160. <https://doi.org/10.1007/s13225-018-0408-6>

Untereiner WA, Débois V, Naveau FA (2001) Molecular systematics of the ascomycete genus *Farrowia* (*Chaetomiaceae*). Canadian Journal of Botany 79:321–333.

Vu D, Groenewald M, De Vries M, et al. (2019) Large-scale generation and analysis of filamentous fungal DNA barcodes boosts coverage for kingdom fungi and reveals thresholds for fungal species and higher taxon delimitation. Studies in Mycology 92:135–154. <https://doi.org/10.1016/j.simyco.2018.05.001>

Wang WP, Shen HW, Su XJ, et al. (2025) Lignicolous freshwater fungi from karst landforms in China: Introducing *Wongia guttulata* sp. nov. associated with bamboo. Mycological Progress 24:58.

Wang WP, Hyde KD, Bao DF, et al. (2024) Lignicolous freshwater fungi from karst landscapes in Yunnan Province, China. Mycosphere 15:6525–6640.

Win H, Bhunjun CS, Maharachchikumbura SSN (2025) A new lignicolous fungus, *Rhexoacrodictys chiangraiensis* (*Pleurotheciales*, Ascomycota ), from Northern Thailand. New Zealand Journal of Botany 63:597–608. <https://doi.org/10.1080/0028825X.2024.2344800>

Wu W, Diao Y (2022) Anamorphic chaetosphaeriaceous fungi from China. Fungal Diversity 116:1–546. <https://doi.org/10.1007/s13225-022-00509-w>

Xia JW, Ma YR, Li Z, et al. (2017) Acrodictys-like wood decay fungi from southern China, with two new families *Acrodictyaceae* and *Junewangiaceae*. Scientific Reports 7:7888. <https://doi.org/10.1038/s41598-017-08318-x>

Xu CY, Song HY, Zhou JP, et al. (2025a) Four New or Newly Recorded Species from Freshwater Habitats in Jiangxi Province, China. Journal of Fungi 11:79. <https://doi.org/10.3390/jof11010079>

Xu RJ, Hyde KD, Li JN, et al. (2025b) Lignicolous freshwater fungi of the pan Qinghai-Xizang Plateau, China. Fungal Diversity 133:23–234. <https://doi.org/10.1007/s13225-025-00555-0>

Xiong YC, Xu RJ, Luo ZL, et al. (2024) *Sporidesmiella motuoensis*, a new freshwater fungus from Tibetan Plateau, China. Phytotaxa 635:105–112.

Yang J, Liu LL, Jones EBG, et al. (2023a) Freshwater fungi from karst landscapes in China and Thailand. Fungal Diversity 119:1–212. <https://doi.org/10.1007/s13225-023-00514-7>

Yang EF, Dai DQ, Bhat JD, et al. (2023b) Taxonomic and phylogenetic studies of saprobic fungi associated with *Mangifera indica* in Yunnan China. Journal of Fungi 9:680. <https://doi.org/10.3390/jof9060680>

Yang J, Maharachchikumbura SSN, Liu JK, et al. (2018) *Pseudostanjehughesia aquitropica* gen. et sp. nov. and *Sporidesmium sensu* lato species from freshwater habitats. Mycological Progress 17:591–616.

Yang J, Liu LL, Jones EBG, et al. (2021) Morphological Variety in *Distoseptispora* and Introduction of Six Novel Species. Journal of Fungi 7:945. <https://doi.org/10.3390/jof7110945>

Yang JX, Zhang H, Wang L, et al. (2023c) *Phaeoisaria ellipsoidea* (*Pleurotheciaceae, Pleurotheciales, Sordariomycetes*), a new lignicolous freshwater fungus from Xishuangbanna, Yunnan Province, China. Phytotaxa 591:19–30. <https://doi.org/10.11646/phytotaxa.591.1.2>

Yuan HS, Lu X, Dai YC, et al. (2020) Fungal diversity notes 1277–1386: taxonomic and phylogenetic contributions to fungal taxa. Fungal Diversity 104:1–266.

Yu XD, Zhang SN, Liu JK (2024a) Novel hyphomycetous fungi associated with bamboo from Sichuan, China. Phytotaxa 634:235–254.

Yu X, Zhang S, Liang X, et al. (2024b) Bambusicolous Fungi from Southwestern China. Mycosphere 15:5038–5145. <https://doi.org/10.5943/mycosphere/15/1/24>

Zhang JY, Hyde KD, Bao DF, et al. (2025) A worldwide checklist and morpho-molecular systematics of fungi associated with pteridophytes. Fungal Diversity: 1–273

Zhang H, Dong W, Hyde KD, et al. (2017a) Towards a natural classification of Annulatascaceae-like taxa: introducing *Atractosporales* ord. nov. and six new families. Fungal Diversity 85:75–110.

Zhang ZF, Liu F, Zhou X, et al. (2017b) Culturable mycobiota from Karst caves in China, with descriptions of 20 new species. Persoonia 39:1–31.

Zhang L, Shen HW, Bao DF, et al. (2023) *Wongia suae* sp. nov., a lignicolous freshwater fungus from Yuanjiang (Red River) Basin, China. Phytotaxa 616:258–268. <https://doi.org/10.11646/phytotaxa.616.3.5>

Zhang ZF, Zhou SY, Eurwilaichitr L, et al. (2021) Culturable mycobiota from Karst caves in China II, with descriptions of 33 new species. Fungal Diversity 106:29–136.

Zhai ZJ, Yan JQ, Li WW, et al. (2022) Three novel species of *Distoseptispora* (*Distoseptisporaceae*) isolated from bamboo in Jiangxi Province, China. MycoKeys 88:35–54. <https://doi.org/10.3897/mycokeys.88.79346>

Zhang H, Zhu R, Qing Y, et al. (2022) Polyphasic Identification of *Distoseptispora* with Six New Species from Fresh Water. Journal of Fungi 8:1063. <https://doi.org/10.3390/jof8101063>

Zhao H, Mapook A, Hyde KD, et al. (2025) *Xylolentia bambusae* (*Rhamphoriaceae, Rhamphoriales*), a novel species associated with dead bamboo in Guangdong Province, China. Phytotaxa 681:211–222. <https://doi.org/10.11646/phytotaxa.681.2.6>
